# *Pseudomonas* Phage Lydia and the Evolution of the *Mesyanzhinovviridae* Family

**DOI:** 10.3390/v17030369

**Published:** 2025-03-04

**Authors:** Konstantin Troshin, Nina Sykilinda, Sofia Shuraleva, Anna Tokmakova, Nikolay Tkachenko, Lidia Kurochkina, Konstantin Miroshnikov, Natalia Suzina, Ekaterina Brzhozovskaya, Kristina Petrova, Stepan Toshchakov, Peter Evseev

**Affiliations:** 1 Laboratory of Molecular Microbiology, Pirogov Russian National Research Medical University, Ostrovityanova 1, 117997 Moscow, Russia; konstantinetr@gmail.com (K.T.); shuralyova_sa@rsmu.ru (S.S.); ntkachenko037@gmail.com (N.T.); emmbf@yandex.ru (E.B.); 2 Shemyakin-Ovchinnikov Institute of Bioorganic Chemistry, Russian Academy of Sciences, Miklukho-Maklaya Str. 16/10, 117997 Moscow, Russia; sykilinda@mail.ru (N.S.); anna.zem@mail.ru (A.T.); kmi@bk.ru (K.M.); 3 Moscow Center for Advanced Studies, Kulakova Str. 20, 123592 Moscow, Russia; 4 Belozersky Institute of Physico-Chemical Biology, Lomonosov Moscow State University, Leninskie Gory 1 Bld 40, 119991 Moscow, Russia; lpk@belozersky.msu.ru; 5 Skryabin Institute of Biochemistry and Physiology of Microorganisms, Federal Research Center “Pushchino Center for Biological Research of the Russian Academy of Sciences”, Prosp. Nauki 5, 142290 Pushchino, Russia; suzina_nataliya@rambler.ru; 6 Center for Genome Research, National Research Center “Kurchatov Institute”, Kurchatov Sq. 1, 123098 Moscow, Russiastepan.toshchakov@gmail.com (S.T.)

**Keywords:** phage, *Mesyanzhinovviridae*, phage evolution, *Pseudomonas aeruginosa*, phage genomics, phage therapy

## Abstract

Phage Lydia, a newly isolated siphovirus infecting *Pseudomonas aeruginosa*, was characterized with respect to its basic kinetic properties and subjected to comparative bioinformatic analysis with related phages. The phage exhibited a restricted host range, with lytic activity observed against 7 of 30 tested isolates. The genome of phage Lydia consists of a 61,986 bp dsDNA molecule and contains 89 predicted genes. Bioinformatic analysis suggests the presence of a DNA modification system, but no apparent genes associated with lysogeny or antibiotic resistance were identified. Taxonomic classification places Lydia within the *Mesyanzhinovviridae* family, *Rabinowitzvirinae* subfamily, and Yuavirus genus, with the closest relation to *Pseudomonas* virus M6. Comprehensive bioinformatic studies, including structural modelling and analysis of phage proteins, as well as comparative taxonomic, phylogenomic, and pangenomic analyses of the *Mesyanzhinovviridae* family, revealed relationships between proteins of *Mesyanzhinovviridae* phages, proteins from other phage groups, encapsulins, and a gene transfer agent (GTA) particle from *Rhodobacter capsulatus*. These analyses uncovered patterns of evolutionary history within the family, characterized by genetic exchange events alongside the maintenance of a common genomic architecture, leading to the emergence of new groups within the family.

## 1. Introduction

Bacteriophages, or phages, are viruses that specifically infect bacteria. They are the most abundant biological entities on Earth, playing a significant role in shaping microbial communities across diverse environments, ranging from terrestrial and aquatic ecosystems to the human microbiome [1]. Phages are also versatile tools in biotechnology, offering potential applications in diagnostics, biocontrol, and therapeutics [2]. In particular, the increasing prevalence of antibiotic resistance has renewed interest in phage therapy, which utilizes phages as highly specific, self-replicating antibacterial agents [3]. Furthermore, phages are valuable objects in evolutionary biology because their diverse proteins and genetic architectures provide insights into the mechanisms underlying viral evolution [4,5]. The taxonomy of bacteriophages is also rapidly evolving, reflecting ongoing exploration of their diversity and functionality [6]. Understanding this expanding knowledge base enables advancements in fundamental virology and drives the development of phage-based solutions for tackling bacterial infections with increasing resistance to conventional antibiotics.

*Pseudomonas aeruginosa*, a Gram-negative bacterium, is a prominent cause of opportunistic infections. Widely distributed in diverse environments, including water, soil, plants, and healthcare facilities, this bacterium readily colonizes human mucous membranes [7]. *P. aeruginosa* may cause severe infections, such as ventilator-associated pneumonia [8], otitis media [9], urinary tract infections [10], bloodstream infections [11], and burn wound infections [12], especially in immunocompromised patients. The associated mortality, which can reach 40% [13], underscores the challenges presented by this pathogen. *P. aeruginosa* possesses an extensive repertoire of virulence factors, including extracellular polysaccharides that facilitate biofilm formation, increase the viscosity of bronchial secretions, impair bronchial drainage, and exacerbate inflammation [8]. A major concern is the escalating resistance of *P. aeruginosa* to antibiotics, which has contributed to its classification as a multidrug-resistant ESKAPE pathogen [14]. The growing shortage of effective antibiotics highlights the need to explore alternative approaches. Phage therapy, which utilizes viruses to specifically target and lyse bacterial pathogens, has demonstrated considerable promise as a potential treatment, particularly for infections caused by multidrug-resistant strains of *P. aeruginosa* [15]. Preclinical studies in animal models and clinical trials have indicated that phages can effectively target *P. aeruginosa*, suggesting a potential strategy for addressing this problematic pathogen [13,16].

As of December 2024, the GenBank PHG database contained 49,289 records, with 1783 specifically attributed to *Pseudomonas* phages. NCBI taxonomy annotations indicate that 1030 of these are tailed phages (class *Caudoviricetes*). The classified *Pseudomonas* tailed phages are attributed to eleven families (*Arenbergviridae*, *Autographiviridae*, *Casjensviridae*, *Chimalliviridae*, *Drexlerviridae*, *Fredfastierviridae*, *Mesyanzhinovviridae*, *Peduoviridae*, *Schitoviridae*, *Straboviridae*, and *Zobellviridae*), five subfamilies not assigned to families, and thirty-seven genera not assigned to families or subfamilies. The *Mesyanzhinovviridae* family, which includes the newly isolated phage Lydia, is well represented, with 41 GenBank PHG genomes of phages infecting *Pseudomonas* bacteria. These 41 phages represent the majority of the phages within the *Mesyanzhinovviridae* family that are recorded in the GenBank PHG database (75 phages; two sequences were found to be misclassified). In addition, *Mesyanzhinovviridae* phages infect alpha-, beta-, and gamma-proteobacteria, including an alpha-proteobacterium isolated from the marine sponge *Ircinia strobilina* [17], *Stenotrophomonas maltophilia* [18], *Xanthomonas campestris* [19] and other *Xanthomonas* bacteria [20,21], and *Bordetella bronchiseptica* [22], which can be associated with human infections [23,24] or plant diseases [25]. A significant portion of the *Mesyanzhinovviridae* phage sequences in GenBank PHG (50 records) include an “isolation source” annotation, with the majority from various water sources, followed by several from soil, and a few from other diverse sources.

A notable feature reported for several *Mesyanzhinovviridae* phages is the presence of DNA modifications, either experimentally observed or bioinformatically predicted [17,18,26,27,28,29,30], as well as the presence of unusual enzymes involved in nucleotide metabolism and DNA processing [31,32]. The lifestyle of *Mesyanzhinovviridae* phages is also a topic of interest, particularly considering their potential use in phage therapy. Several studies have provided evidence of experimentally detected or bioinformatically predicted temperate infection cycles [17,18,22,28,29,33,34,35]; in contrast, other studies emphasize a lytic lifestyle, indicating potential for use of these phages in phage therapy or biocontrol [19,20,30,36,37,38,39,40]. The lytic activity is sometimes described as moderate, but genome-encoded DNA modification systems can be a notable advantage for using *Mesyanzhinovviridae* phages.

The present study aims to identify patterns in the genomics of *Mesyanzhinovviridae* phages and to elucidate common characteristics of phages within this family, focusing on the newly characterized phage Lydia, a *P. aeruginosa*-infecting phage that belongs to the *Yuavirus* genus. First, the biological characteristics of phage Lydia will be described. This will be followed by a bioinformatic analysis of Lydia’s genome and proteins. Subsequently, a comparative taxonomic, phylogenomic, and pangenomic analysis of the *Mesyanzhinovviridae* family will be conducted. Finally, the results of these analyses will be discussed, and relevant evolutionary suggestions and hypotheses will be proposed.

## 2. Materials and Methods

### 2.1. Phage Isolation and Purification

*Pseudomonas aeruginosa* PAO1 from the collection of Professor V.N. Krylov (I.I. Mechnikov Research Institute of Vaccines and Serums, Moscow) served as the host strain for phage propagation. To enrich for phages, a 3 mL water sample from Selenga River (51°50′ N, 107°34′ E) was supplemented with 1 mL of 4× Lysogeny broth (LB) (Becton-Dickinson, Franklin Lakes, NJ, USA), and 40 µL of an overnight culture of *P. aeruginosa* PAO1. After an 18 h incubation at 37 °C, chloroform (Aldosa, Moscow, Russia) (0.5% *v*/*v*) was added and incubated for 4 h at 4 °C, followed by centrifugation. The presence of phages was confirmed using a double-agar layer technique [41], with a soft agar overlay containing 0.7% agar. Serial dilutions of the phage-containing supernatant were plated on a *P. aeruginosa* lawn, and plaques were counted after incubation for 18–24 h. To propagate the phage from a single plaque, we infected *P. aeruginosa* PAO1 in a liquid culture at 37 °C, and once lysis was complete, the phage lysate was precipitated using polyethylene glycol (10%) (NeoFroxx, Einhausen, Germany)/NaCl (1M) followed by centrifugation at 8000× *g* (Eppendorf centrifuge 5430R, Eppendorf AG, Hamburg, Germany), resuspension in SM buffer (50 mM Tris-HCl, 100 mM NaCl, 8 mM MgSO_4_, pH 7.5), incubation with KCl, and another centrifugation step at 20,000× *g*. The resulting phage stock had a titer of ~10^11^ PFU/mL and stored at 4 °C until used.

### 2.2. Phage Stability Under Different Conditions

Phage stability under various conditions was assessed following a modified protocol based on [42]. To determine temperature stability, a phage suspension was prepared in the SM buffer to a final concentration of 4 × 10^7^ PFU/mL. This suspension was then incubated at −20 °C (freezer), +4 °C, +28 °C, +37 °C, +50 °C, and +60 °C for 1 h using a Thermomixer F 2.0 (Eppendorf, Hamburg, Germany). To assess pH stability, a 0.9% NaCl solution was adjusted with NaOH and HCl to pH values ranging from 3 to 11. These solutions were added to the samples, achieving a final phage titer of 4 × 10^7^ PFU/mL, and incubated at 25 °C for 1 h. To determine UV resistance, high-titer phage samples (3 × 10^8^ PFU/mL) were exposed to a PL-S 9W/12/2p UV lamp (Philips, Amsterdam, The Netherlands) (280–315 nm) for 50 min. Aliquots of 50 µL were collected every 10 min into separate tubes. Chloroform sensitivity was evaluated by mixing phage solutions with chloroform to achieve final concentrations of 0%, 5%, 25%, 50%, and 75% (*v*/*v*), resulting in a final titer of 3 × 10^8^ PFU/mL. These mixtures were incubated at 37 °C for 30 min with shaking (ES-20 orbital shaker, Biosan, Hangzhou, China), according to [43]. Subsequently, the solutions were centrifuged at 10,000× *g* for 10 min, and the supernatant was collected. All solutions obtained were titrated using the LB top agar method. Following 24 h of cultivation, the titer in each sample was determined. All experiments were performed with three parallel repetitions.

### 2.3. Determination of Phage Host Range

The phage lytic activity and host range were evaluated against 30 clinical and environmental *P. aeruginosa* isolates using the double-layer agar assay. Clinical isolates, described in [44], were obtained from Russian Children’s Clinical Hospital (Moscow). Overnight cultures of *P. aeruginosa* (200 μL) were mixed with 3 mL of 0.75% soft agar and plated. Phage suspensions (~10^9^ PFU/mL) were then spotted onto the bacterial lawns and incubated at 37 °C for 18–24 h. The plates were subsequently examined to assess phage lytic activity and host specificity.

### 2.4. Phage Adsorption and One-Step Growth Experiments

The phage adsorption curve was constructed according to a standard method with some modifications [45]. To determine the adsorption rate, exponentially growing bacterial cells were mixed with the bacteriophage at a multiplicity of infection (MOI) of 0.001 and incubated at 37 °C. Samples (100 µL) were taken at 2, 4, 6, 8, 10, and 15 min, mixed with 850 µL of SM buffer and 50 µL of chloroform. Following centrifugation at 10,000× *g*, the supernatant was titrated to determine the number of unadsorbed or reversibly adsorbed phages at each time point. Phage adsorption was quantified using the adsorption rate constant (k), measured in mL/min [41], as defined by the following formula: k = 2.3/(B × t) × log (P_0_/P), where P_0_ is the phage titer determined at time 0, P is the phage titer remaining unadsorbed at time t, B is the initial bacterial titer per mL, and t is the time in min. The adsorption curve was constructed by plotting the ratio of unadsorbed phage to the initial phage titer. The experiment was performed in triplicate.

To determine the single-cycle burst size, 20 mL cultures of *P. aeruginosa* PAO1 at OD_600_ ~0.4 were centrifuged at 8000× *g* (Eppendorf 5430R, Eppendorf AG, Hamburg, Germany), resuspended in 0.5 mL of LB, and infected with 100 µL of phage (~3 × 10^8^ PFU/mL) at an MOI of ~0.01, and incubated for 7 min. Unadsorbed phages were removed by centrifugation for 2 min at 13,000× *g*, and the pellet was resuspended in 10 mL of LB. The suspension was incubated in 50 mL tubes at +37 °C on a shaker for 120 min, with 100 µL samples taken at the start time and at 10 min intervals up to 120 min from the start of the experiment. The experiment was performed in triplicate. For PFU/mL counts, the samples were titrated and 10 µL aliquots were spotted onto double-layer agar, and plaques were counted after 24 h. Phage burst size was calculated as the ratio of average titer at the plateau after the burst to the total number of phages used to infect the cells at t0 [46].

### 2.5. Transmission Electron Microscopy

The morphology of phage Lydia was visualized using transmission electron microscopy. Concentrated and purified phage samples were prepared by adsorption onto grids followed by negative staining with 1% uranyl acetate (pH 4.0). Images were acquired using a Hitachi H-300TM electron microscope (Hitachi, Tokyo, Japan).

### 2.6. Genome Sequencing and Annotation

Phage DNA was isolated via phenol–chloroform extraction and subsequently fragmented using a Bioruptor sonicator (Diagenode, Liège, Belgium). Paired-end libraries were constructed using a Nebnext Ultra DNA library prep kit (New England Biolabs, Ipswich, MA, USA), and sequenced on the Illumina MiSeq platform (paired 150 bp reads). Data were filtered using CLC Genomics Workbench v8.5 (QIAGEN, Aarhus, Denmark), after which overlapping paired-end reads were merged using SeqPrep v1.2. Finally, the reads were assembled into contigs using CLC Genomic Workbench v8.5.

Open reading frames (ORFs) were identified using Glimmer v3.02b [47] and Prodigal v2.6.3 [48]. Gene boundaries were manually curated. Functional annotation of the predicted genes was performed using a combination of sequence similarity and structure-based approaches. First, BLAST searches [49] were conducted against the NCBI nr/nt and GenBank PHG databases (https://www.ncbi.nlm.nih.gov, accessed on 10 December 2024). Then, HHpred [50] searches were performed against the PDB70_mmCIF70_16_Aug, PfamA-v37, UniProt-SwissProt-viral70_3_Nov_2021, and NCBI_Conserved_Domains(CD)_v3.19 databases (https://toolkit.tuebingen.mpg.de, accessed on 10 December 2024). Finally, DALI searches [51] were executed using predicted protein structures and the PDB search implemented in the DALI server (http://ekhidna2.biocenter.helsinki.fi/dali, accessed on 10 December 2024). Default settings were used for all search engines. Putative functions were assigned based on a BLAST E-value threshold of 1 × 10^−5^, an HHpred probability of 95%, and a DALI Z-score of 10. The annotated genome was deposited in the NCBI GenBank database under accession number PV031332.

### 2.7. Bioinformatic Analyses

Multiple sequence alignments of nucleotide and amino acid sequences were generated using MAFFT version 7.48 [52] with default settings and the L-INS-i algorithm. Phylogenetic analysis of the aligned viral sequences was performed using IQ-TREE version 2.2.5 [53] with the command-line parameters “-m TEST-ninit 1000-bb 1000”. These parameters employed ModelFinder [54] to determine the optimal substitution model. Intergenomic comparisons of phages were conducted using VIRIDIC v1.1 [55] (https://rhea.icbm.uni-oldenburg.de/viridic, accessed 10 December 2024), applying default settings. Bacterial average nucleotide identity (ANI) calculations were performed using FastANI [56]. The ANI heatmap was constructed using ANIclustermap (https://github.com/moshi4/ANIclustermap, accessed 10 December 2024). A phylogenetic tree based on predicted proteome similarity (a “proteomic tree”) was constructed using the ViPTree server [57] (https://www.genome.jp/viptree/, accessed 10 December 2024) with default settings. All phylogenetic trees were visualized using iTOL v7 [58]. Protein structures were predicted using AlphaFold 3 (AF3) [17] and visualized with PyMOL v2.5.4 (Schrödinger Inc., New York, NY, USA). The highest-ranked AF3 models were used for structural analyses and comparisons. Protein structural similarity was assessed using the DALI Z-score [51]. Pangenomic analysis was performed using anvi’o v8 [59] and the anvi’o pangenomic pipeline [60], with a 30% amino acid identity threshold for single-copy core genes (SCGs), and all other parameters set to their defaults. The presence of genes encoding antibiotic resistance was determined through BLAST search using the Comprehensive Antibiotic Resistance Database (CARD) v3.2.7. Proteins potentially associated with lysogeny were identified using HHblits v3.3.0 [61] with default settings and the databases pdb70_from_mmcif_2023-06-18, pfama-v35, and uniprot_sprot_vir70_Nov_2021. This was followed by a keyword search for “excisionase”, “integrase”, “recombinase”, and “repressor” using the custom script, and then verified by manual inspection.

## 3. Results

### 3.1. General Biological Features of Phage Lydia

#### 3.1.1. Latency Period and Burst Size Determination

**Figure 1:**
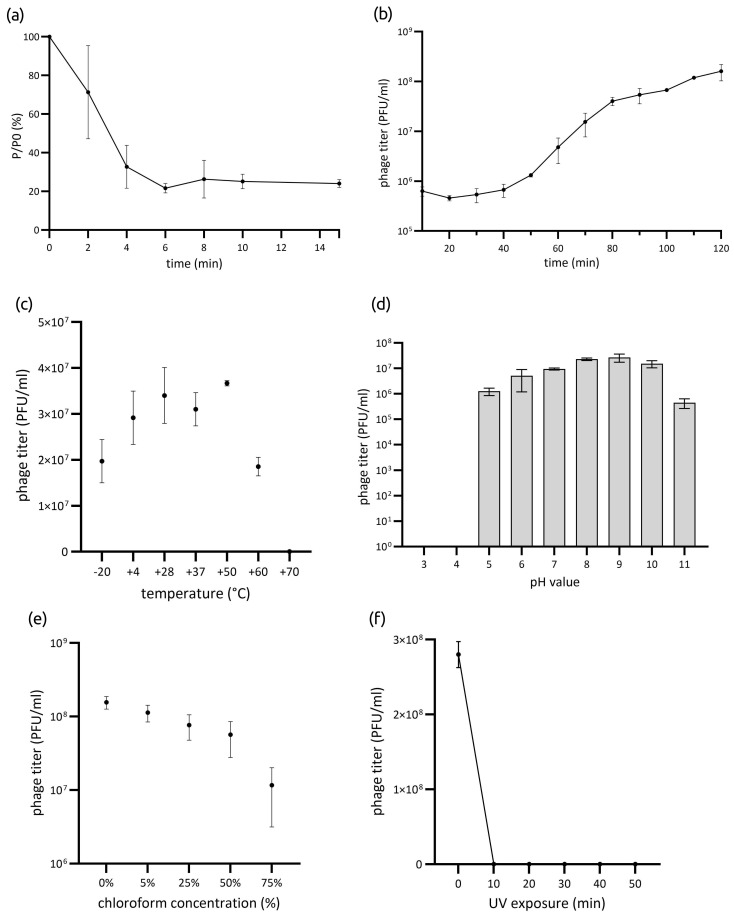
(**a**) Adsorption of phage Lydia at the surface of *P. aeruginosa* PAO1 in MOI = 0.001. (**b**) One-step growth curve of phage Lydia using *P. aeruginosa* PAO1 as the host strain in MOI = 0.01. Data points indicate the PFU/mL at different time points. (**c**) Temperature stress (−20 °C to 70 °C for 1 h); (**d**) pH stress (pH 3–11 for 1 h); (**e**) chloroform treatment (5–75% *v*/*v*); and (**f**) UV irradiation (10–50 min). Data represent the mean of three independent replicates; error bars indicate standard deviation (SD).

Phage Lydia exhibited rapid adsorption to its host bacterium, with approximately 78% of phage particles adsorbed within 6 min of introduction at +37 °C (Figure 1a). One-step growth experiments revealed a latent period of 40 min, followed by a 40 min rise period, reaching a plateau by 80 min. The burst size was determined to be 147 ± 6 phage particles per infected bacterial cell (Figure 1b).

#### 3.1.2. Phage Host Range

The host range of bacteriophage Lydia was characterized by testing its lytic activity against 30 *Pseudomonas aeruginosa* isolates. This panel included 28 clinical isolates, the *P. aeruginosa* PAO1 type strain, and a laboratory-adapted PAO1 strain from the collection of Professor V.N. Krylov (Supplementary Table S1). The phage exhibited a restricted host range, with lytic activity observed against only 7 of the 30 isolates. These susceptible isolates were found to represent sequence types ST200, ST235, ST245, ST274, ST549, and ST942.

#### 3.1.3. Phage Stability

The infectivity of phage Lydia after exposure to various temperatures for 1 h remained relatively stable between −20 °C and +60 °C. Specifically, the phage titer decreased by 50.8% at −20 °C and by 53.7% at +60 °C. However, exposure to +70 °C resulted in a complete loss of phage viability after 1 h of incubation (Figure 1c). Optimal pH values for phage Lydia ranged from 7 to 10. Exposure to pH values between 3 and 6, or to pH 11, resulted in a partial or complete loss of viability (Figure 1d). The phage titer decreased in correlation with increasing chloroform concentration (Figure 1e). After incubation in 75% chloroform solution, Lydia retained approximately 4% of its initial activity. Exposure to UV irradiation resulted in the complete inactivation of phage virions after 10 min (Figure 1f).

#### 3.1.4. Phage Morphology

**Figure 2:**
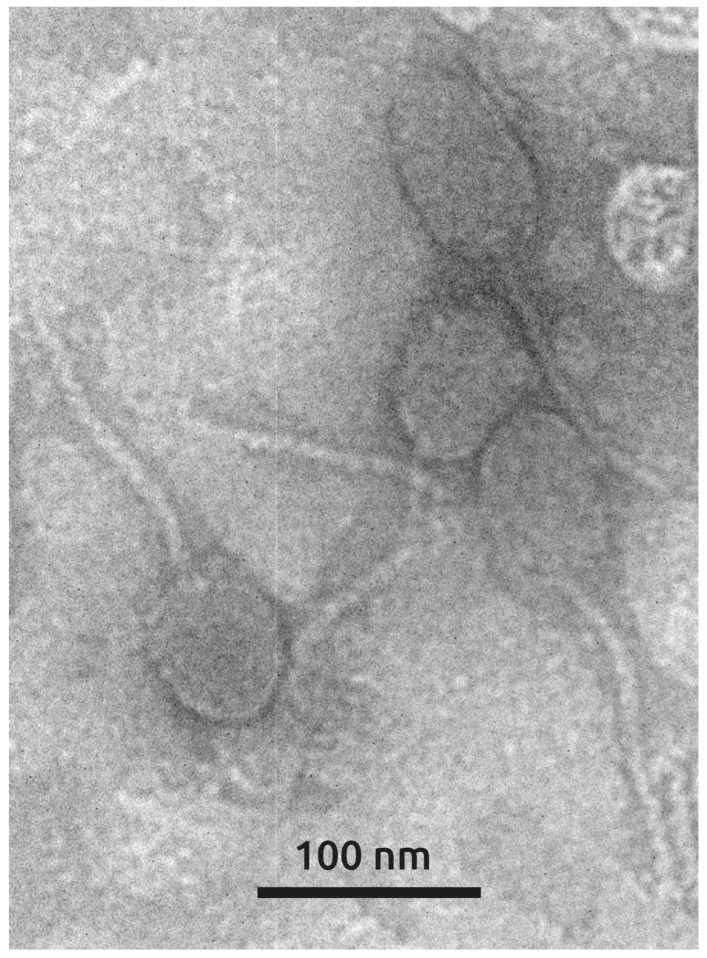
Electron microscopic image of phage Lydia particles. Scale bar is 100 nm.

Transmission electron microscopy revealed that phage Lydia exhibits a morphology characteristic of siphoviruses, with an oblate head elongated along the tail axis (approximately 70 × 50 nm) and a flexible tail of ~150 nm in length. This morphology places phage Lydia into morphotype B2. These features are reminiscent of phages YuA [28] and AN14 [36] and other related *Pseudomonas aeruginosa*-infecting phages classified within the *Yuavirus* genus of the *Rabinowitzvirinae* subfamily of the *Mesyanzhinovviridae* family (Figure 2).

### 3.2. Genome and Proteome Characterization of Phage Lydia

#### 3.2.1. General Features

The isolation of *Pseudomonas* phage Lydia was performed as part of this study and is described in detail in Section 2. Briefly, phage Lydia was isolated from Selenga River using *Pseudomonas aeruginosa* PAO1 from the collection of Professor V.N. Krylov as the host bacterium. The isolation process resulted in clear plaques, as shown in Supplementary Figure S1.

**Figure 3:**
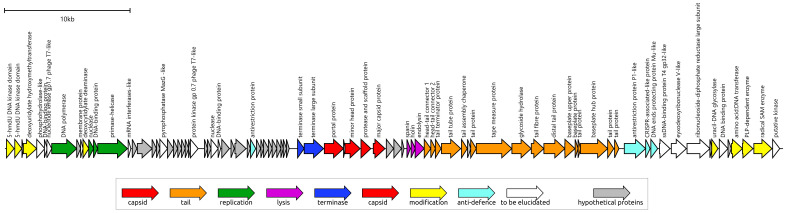
Genetic map of phage Lydia. Gene annotations and predicted functions are indicated by labels and a legend. Arrows show the direction of transcription for each gene. The scale bar represents the length of the nucleotide sequence.

The *Pseudomonas* phage Lydia genome comprises a double-stranded DNA molecule of 61,986 base pairs (NCBI Accession # PV031332) and contains 89 predicted open reading frames (ORFs) (Supplementary File S1). Analysis revealed the absence of any tRNA genes. All predicted protein products (gp) encoded by these ORFs were modelled using AlphaFold 3 (AF3) (Supplementary File S2). A combination of BLAST and HHpred searches, along with structural comparisons to PDB entries using the DALI search, allowed for the functional annotation of 56 protein products (Figure 3). The remaining 33 predicted ORFs were annotated as encoding hypothetical proteins. No antibiotic resistance genes were found in the genome.

BLAST analysis of the *Pseudomonas* phage Lydia genome sequence indicated a similarity to *Pseudomonas* phage M6, a phage classified by the ICTV within the *Mesyanzhinovviridae* family (*Pseudomonas* virus M6, genus *Yuavirus*, subfamily *Rabinowitzvirinae*). Comparative genomics of *Mesyanzhinovviridae* phages has revealed that they lack a specific genome start site, suggesting a circularly permuted genome and a headful packaging mechanism [62]. In accordance with this, the initial assembly of the phage Lydia genome contained direct terminal repeats of 127 bp, the right repeat subsequently removed from the final submitted sequence.

The genome of phage Lydia displays a modular architecture common to many phages. Specifically, the genome contains a replication module (including genes for DNA polymerase and a gene encoding a two-domain protein with similarity to primase and helicase, hereafter referred to as ‘primase-helicase’), a packaging module (terminase large and small subunits, TLS and TSS), structural genes for the capsid, lysis genes, a tail module, genes predicted to be involved in counteracting bacterial defence systems, and a cluster of genes potentially involved in DNA modification. Notably, the genome of phage Lydia contains no genes predicted to facilitate a lysogenic lifestyle. Some gene products previously assigned as putative integrases [22,28] were, probably, misannotated. Gene product 14 (gp14) of phage Lydia may serve as an example. Sequence analysis of the gene revealed significant similarity to other genes of phage origin; some of them were marked as integrases, while others were identified as primase-helicases. HMM-based and structural analyses of gp14 conclusively demonstrate that it encodes a protein with distinct primase (N-terminal domain) and helicase (C-terminal domain) activities. This finding is consistent with experimental studies conducted on the *Yuavirus* phage YuA and its *Pseudomonas aeruginosa* PAO1 host, which failed to produce a stable, lysogenic strain [28]. Moreover, the *Yuavirus*-related phage *Pseudomonas* phage AN14 also possesses a primase-helicase gene homologous to phage Lydia gp14 and has not demonstrated a temperate lifestyle in experimental studies [36], further reinforcing the possibly obligate lytic lifestyle of this group of phages.

The *Pseudomonas* phage Lydia genome exhibits a striking enrichment of genes encoding DNA modification enzymes, including a putative deoxyuridylate hydroxymethyltransferase (DUHMT) and 5-hydroxymethyluracil (5hmdU) kinase, both of which are also characteristic of *Yuavirus* phages. These genes are hypothesized to participate in the recently discovered thymidine hypermodification, which leads to the substitution of roughly 30% of thymidine bases with 5-(2-aminoethyl)uridine (5-NedU) [63]. Such modifications are considered to be essential for circumventing bacterial restriction–modification (R-M) systems [64]. In addition to these modification enzymes, the genome also contains genes encoding several antirestriction proteins. One such protein, Lydia gp36, displays high sequence similarity to the KlcA protein from the *Bordetella pertussis* plasmid pBP136 (HHpred probability 100%, PDB structure 2KMG [22]). KlcA, unlike many antirestriction proteins, does not mimic DNA but rather inhibits all four main families of Type I R-M systems [65]. Another predicted antirestriction protein, Lydia gp76, is homologous to the “defence against restriction protein B” (DarB) from *Escherichia* phage P1 (HHpred probability 100%, UniProt #Q71TF8). DarB is believed to be incorporated into the phage capsid and subsequently injected into the host cell alongside the phage DNA [66].

#### 3.2.2. Major Capsid Protein and Terminase Large Subunit

**Figure 4:**
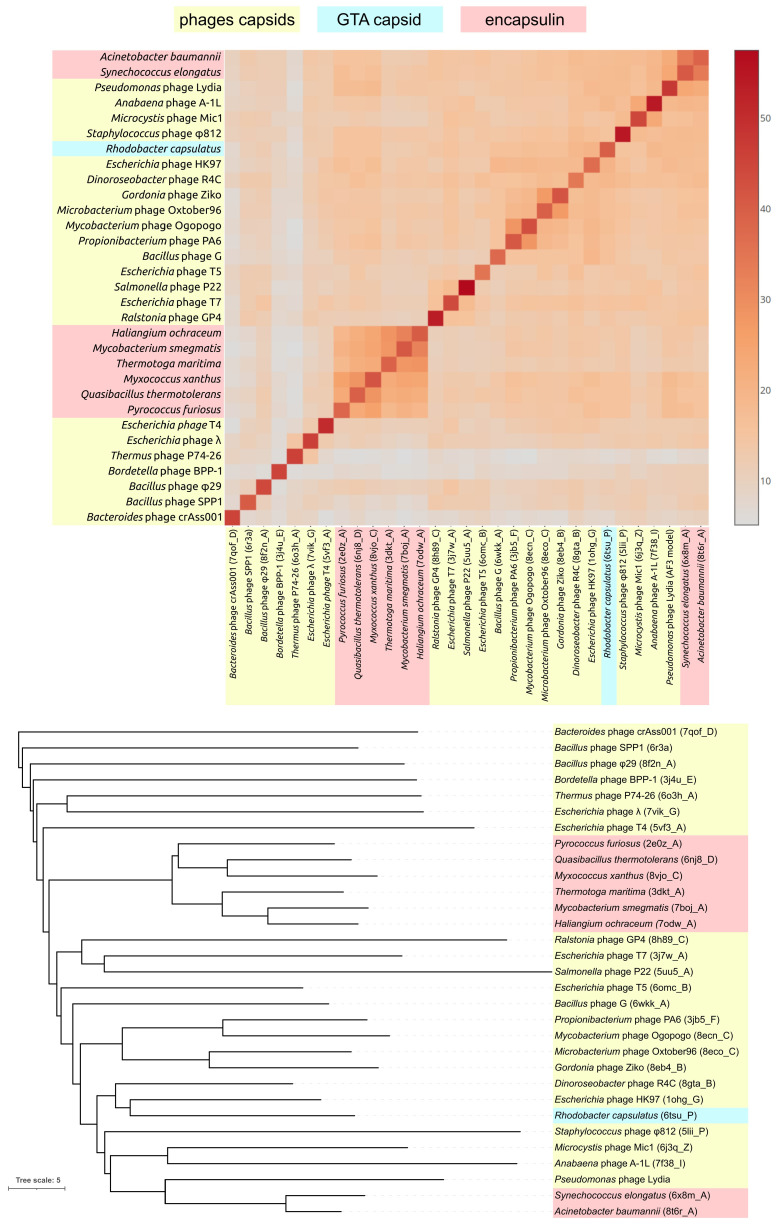
Heatmap and tree constructed based on the pairwise structural similarity of phage and GTA capsid proteins and encapsulin major shell proteins, as measured by DALI Z-scores. Tree branch lengths are measured with DALI Z-scores.

A DALI search unexpectedly revealed that gp52 of phage Lydia, a major capsid protein (MCP), shares remarkable structural similarity with encapsulin shell proteins from *Synechococcus elongatus* PCC 7942 [67], specifically the holo conformation (PDB code 6 × 8m_A, DALI Z-score 23.7, DALI RMSD 2.3 Å, Pymol superposition RMSD 2.3 Å) and the apo form (PDB #6 × 8t_A, Z-score 22.8). This encapsulin, a member of the Family 2A system involved in sulfur metabolism, sequesters a cysteine desulfurase during sulfur limitation [67]. Encapsulin shell proteins (ESPs) from *Acinetobacter baumannii* 118362, also belonging to Family 2A, yielded the third highest similarity score (PDB #8t6r_A, Z-score 22.3 [68]). Subsequent hits included MCPs from the siphovirus *Escherichia* phage HK97 (PDB #2ft1_A, Z-score 18.7 and PDB #2gp1_A, Z-score 18.6) [69], followed by myovirus *Anabaena* cyanophage A-1(L) [70] and *Escherichia* phage HK97 MCPs (DALI Z-scores 18.2–18.4). In contrast to the DALI results, an HHpred search showed that the highest similarity to phage Lydia MCP was with counterparts from actinophages (HHpred probability 99.96%, *Gordonia* phage Ziko [71], and other actinophages). A GTA (gene transfer agent) particle from *Rhodobacter capsulatus* [72] and an encapsulin from *Acinetobacter baumannii* 118362 [68] also showed sequence homology (HHpred probability is also 99.96% in both cases). Structurally, the modelled phage Lydia MCP clustered with encapsulin shell proteins from *Acinetobacter baumannii* and *Synechococcus elongatus*, positioned adjacent to a branch containing cyanophage MCPs (Figure 4). An analysis of the major capsid proteins (MCPs) from other *Mesyanzhinovviridae* phages yielded comparable results. However, the MCP of the Alphaproteobacteria phage PhiJL001 did not conform to this pattern. Specifically, an HHpred search failed to identify remote homology (sequence similarity detectable by sensitive methods such as HHpred, which can identify relationships between distantly related proteins that are not apparent with standard tools like BLAST) between the PhiJL001 MCP and encapsulin shell proteins.

**Figure 5:**
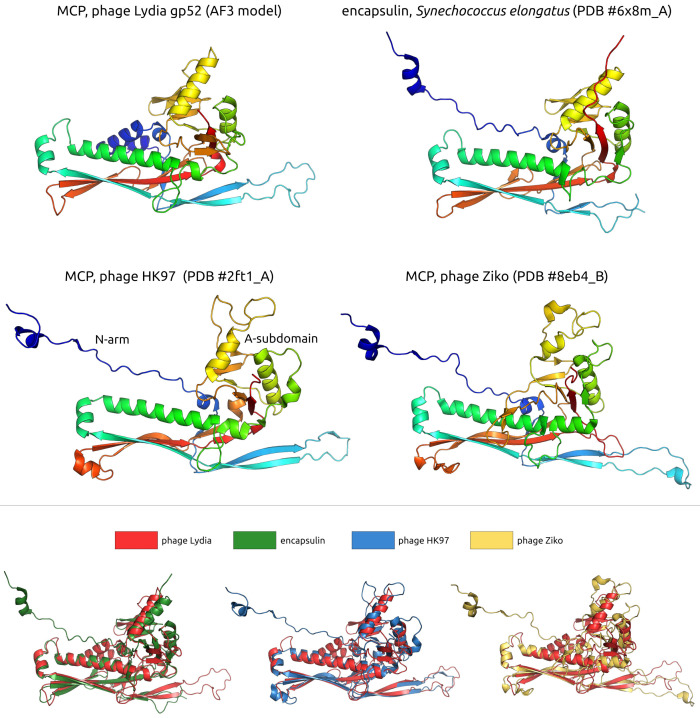
(**Top panel**): A ribbon representation of predicted and experimentally determined structures of major capsid and encapsulin shell proteins. Rainbow colouring uses a colour gradient where the N-terminal end is blue and the C-terminus is red. (**Bottom panel**): The protein structures shown in the top panel are superimposed in PyMOL and coloured according to the legend.

In addition, AlphaFold 3 (AF3) modelling of the phage Lydia major capsid protein revealed subtle structural variations when compared to its closest homologues identified by DALI and HHpred, including the well-characterized MCP of phage HK97. Specifically, the Lydia MCP AF-model possesses a short α-helical N-terminus (approximately residues 1–30), which was predicted with reduced confidence, and corresponds to the N-terminus and N-arm of HK97 MCP. Additionally, the Lydia MCP features a short β-sheet (residues 234–240) within its A-domain (Figure 5).

Phage Lydia, like most tailed bacteriophages excluding φ29-like phages, encodes a terminase with large (TLS, gp46) and small (TSS, gp47) subunits. Phylogenetic analysis of the phage Lydia TLS showed the expected high homology to *Mesyanzhinovviridae* phages as well as some other phages in the *Steinhofvirus*, *Queuovirinae*, *Beaumontvirinae*, and *Septimatrevirus* genera. Notably, both HHpred and DALI structural analyses identified the TLS of the thermophilic *Geobacillus stearothermophilus* phage D6E [73] as the closest structural homologue (PDB: 5oe8_B; HHpred probability: 100%, E-value: 3.5 × 10^−38^; DALI Z-score: 20.8). Structural alignment of the ATPase domains from the AlphaFold 3 (AF3)-predicted model of the phage Lydia TLS and the D6E TLS yielded a root-mean-square deviation (RMSD) of 2.4 Å. Furthermore, the conserved arginine residues R188 (Lydia) and R158 (D6E) occupy similar positions within the structures. The TLS of phage D6E, and those of other thermophilic phages, exhibit structural differences compared to some other terminases; notably, like the φ29 packaging ATPase, they possess a conserved trans-arginine finger (R158) that is positioned similarly to that of the HerA/FtsK superfamily and is required for ATP hydrolysis coupling between subunits within the active ring-like assembly [73].

#### 3.2.3. DNA Polymerase

BLAST analysis of the phage Lydia DNA polymerase I (DNAP) identified the highest sequence similarity to its counterparts from other *Mesyanzhinovviridae* phages. Other phages with lower, but notable, sequence similarity included various unclassified phages and those belonging to the *Nanhaivirus*, *Rosemountvirus*, and *Loughboroughvirus* genera. Genomic analysis indicates that *Nanhaivirus* phages are siphoviruses that, like phage Lydia, contain nucleotide-modifying enzymes. In contrast, *Rosemountvirus* and *Loughboroughvirus* phages appear to be myoviruses and lack the extensive repertoire of nucleotide-modifying enzymes typically found in *Mesyanzhinovviridae* phages. This incongruence in the taxonomic origins of the closest homologues for the MCP, TLS, and DNAP of phage Lydia points to a complex evolutionary history for this phage genome. Further HHpred analysis supported the relationship of phage Lydia DNAP to other phage polymerases and revealed a connection to eukaryotic DNA polymerase θ (human DNAP θ, PDB #7ZUS_BBB, HHpred probability 100% [74]). DALI analysis of the predicted structure of phage Lydia DNAP revealed a strong structural similarity to the DpoZ polymerase of vibriophage φVC8 (PDB code 7pbk_B, DALI Z-score 31.4 [75]), a PolA family member known for its 2-aminoadenine vs. adenine specificity.

#### 3.2.4. Tail and Adsorption Apparatus

The tail module of the phage Lydia genome encodes proteins characteristic of a siphoviral tail. The gene order and protein homology within this module are largely conserved within the *Mesyanzhinovviridae* family, with notable homology also to members of the *Queuovirinae* family infecting both *Pseudomonas* and other bacteria. The tail tube protein (TTP) of phage Lydia, encoded by gene 62, is a 511-residue multidomain protein predicted to be primarily composed of β-sheets (Supplementary File S2). Structurally, it exhibits significant similarity to the TTP of coliphage T5 (PDB #5ngj_A, DALI Z-score 18.5 [76]). A DALI search also highlighted structural similarities between Lydia’s TTP and the tube protein of the anti-feeding prophage Afp1 (PDB #6rbn_B, DALI Z-score 10.1 [76]). HHpred analysis revealed relatedness between the TTP of phage Lydia and the TTP of the hyperthermophilic *Thermus* phage P74–26 (PDB #8ed0_A, HHpred probability: 99.96%) [77], as well as several TTPs from phage T5 (HHpred probability: 99.67–99.94%). Interestingly, this search also indicated similarity between the C-terminal domain of Lydia’s TTP and the R-type bacteriocin tube protein CD1364 from *Clostridium difficile* (PDB #8ED0_A, HHpred probability: 98.27%), which is part of a contractile injecting system (CIS) [78].

**Figure 6:**
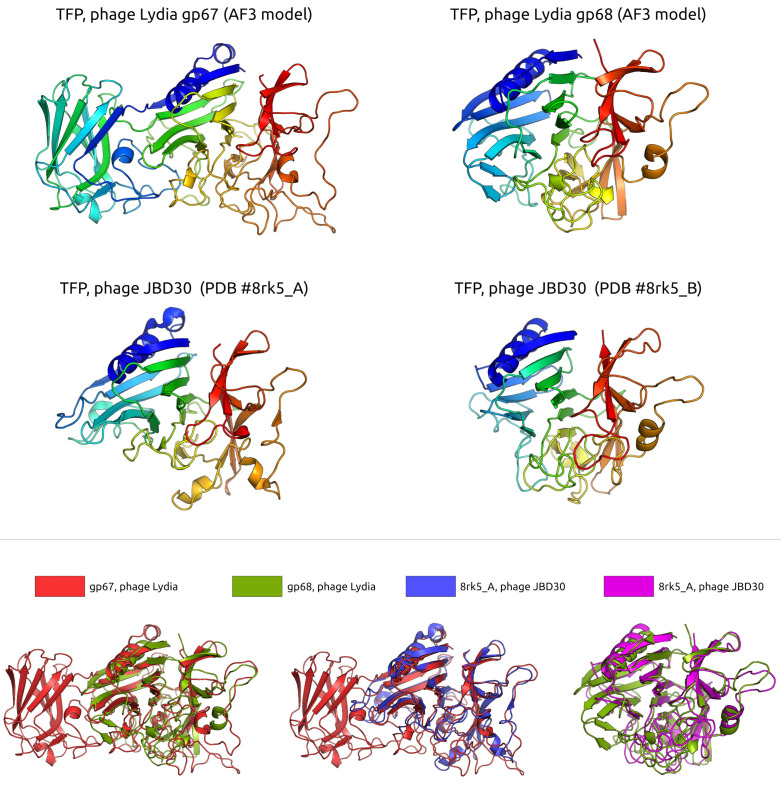
(**Top panel**): A ribbon representation of predicted and experimentally determined structures of tail fibre proteins (TFPs). Rainbow colouring uses a gradient where the N-terminal end is blue and the C-terminus is red. (**Bottom panel**): The protein structures shown in the top panel are superimposed in PyMOL and coloured according to the legend. The superimposition was performed by selecting pairs of tail fibre proteins from phage JBD30 and phage Lydia that yielded the lowest RMSD values, indicating structural similarity.

The tail module of phage Lydia contains several proteins, including gp67–gp69, which are predicted to play roles in the adsorption process (Figure 6). The structural predictions for gene products 67 and 68 suggest that they may form tail fibres, sharing similarities with the tail fibre proteins of *Pseudomonas* phage JBD30 (PDB #8rk5), a *Casadabanvirus* siphovirus known to attach to *P. aeruginosa* via type IV pili [79]. Gene 69 is predicted to encode a distal tail protein, also potentially involved in adsorption. The central and C-terminal regions of the gp69 sequence exhibit homology to the corresponding regions of tail proteins from a diverse range of phages, such as marine roseophage vB_DshS-R4C [80], the aforementioned *Escherichia* phage T5 [81], *Escherichia* phage λ (tail tip protein M), and, interestingly, *Rhodobacter capsulatus* GTA particle [72] (HHpred probability ~97–98%). Gp69 is a predicted multidomain protein (Supplementary File S2). Although DALI searches revealed some structural similarity between gp69 and several proteases, such as *Vibrio cholerae* serine protease VesB (PDB #4lk4, DALI Z-score ~11.0) [82] and *Bacillus amyloliquefaciens* S8 family peptidase Vpr (PDB #8jmw, DALI Z-score ~10) [83], the latter one forms enzymatically active fibres outside the bacteria; further analysis suggests these similarities are restricted to the Ig-like domains, which can be involved in assembly and cell surface interaction [82,83], rather than conferring protease activity. HHpred analysis confirmed the relatedness of gp69 to various phage distal tail proteins and a GTA particle. An analysis of gp70-gp75 indicated their possible role in baseplate structure. However, neither BLAST nor structural searches revealed a peptidoglycan-degrading activity. Thus, based on the structural and sequence homology of these proteins, we hypothesize that gp67–gp69 proteins directly participate in both adsorption and penetration of the host cell. Like in the case of phage JBD30, tail fibres, attached to the upper part of the baseplate, can be composed of three gp67 and gp68 heterodimers [79]. Initial recognition can be performed by baseplate or by gp69.

#### 3.2.5. Lysis Module and Endolysin

The predicted lysis module of phage Lydia includes genes encoding a spanin (gene 56), a holin (gene 57), and an endolysin (gene 58). The translated polypeptide, gp58, the product of gene 58, is a 241-residue protein whose length is typical for many phage lysozymes. Its N-terminal region, which includes the catalytic domain, exhibits significant sequence similarity to a lytic murein transglycosylase from *P. aeruginosa* (PDB code 5ohu_A, HHpred probability 99.78% [84]). This enzyme cleaves the β-1→4 glycosidic bond between N-acetylmuramic acid and N-acetylglucosamine. DALI analysis showed that the most structurally similar protein is another *P. aeruginosa* transglycosylase (PDB #5aa2_D, Z-score 16.4 [85]). A BLAST search revealed homology between the phage Lydia endolysin and endolysins of *Mesyanzhinovviridae* phages belonging to the *Rabinowitzvirinae* subfamily, as well as with endolysins from phages in the *Beetrevirus* and *Phabquatrovirus* genera. However, no significant sequence similarity was detected between the identified endolysin and those from *Mesyanzhinovviridae* phages in the *Bradleyvirinae* subfamily or the genus *Keylargovirus*.

### 3.3. Taxonomic Analysis of Phage Lydia and Phylogenomics of Mesyanzhinovviridae Family

#### 3.3.1. Intergenomic Similarity and Proteome Phylogeny

Calculations of intergenomic similarity using VIRIDIC revealed a high level of sequence identity (>70%) between the genome of phage Lydia and 31 *Mesyanzhinovviridae* phages that infect *P. aeruginosa*, identified through BLAST searches using the Lydia MCP and TLS protein sequences, and intergenomic comparisons (Supplementary Figure S2). Twenty-six of these phages are not yet classified by the ICTV. The remaining phages are officially classified within the *Yuavirus* genus of the *Rabinowitzvirinae* subfamily of the *Mesyanzhinovviridae* family.

According to the International Committee on Taxonomy of Viruses (ICTV) classification as of December 2024, the *Mesyanzhinovviridae* family consists of 33 species (Table 1). These are organized into two subfamilies: 23 species in 11 genera within the *Bradleyvirinae* subfamily, and 9 species in 2 genera (*Vojvodinavirus* and *Yuavirus*) within the *Rabinowitzvirinae* subfamily, in addition to a single species (*Keylargovirus* JL001 infecting an alpha-proteobacteria bacterium) as a separate genus. Notably, the phages that infect *P. aeruginosa* are found in both subfamilies.

**Figure 7:**
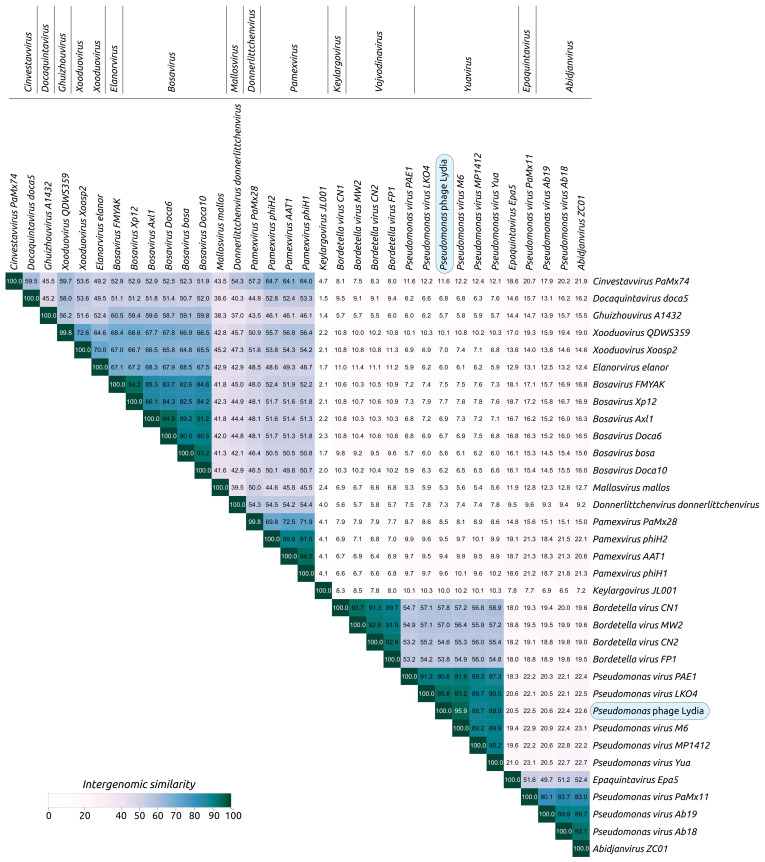
VIRIDIC heatmap generated using genomic sequences of *Mesyanzhinovviridae* phages, classified by the ICTV.

A phylogenetic analysis based on the predicted proteome placed phage Lydia within the *Mesyanzhinovviridae* clade, specifically showing proximity to phages belonging to the *Yuavirus* genus (Supplementary Figure S3). Taken together, the results of the genomic similarity calculations and proteomic phylogenetic analysis allow us to confidently classify phage Lydia within the *Yuavirus* genus of the *Rabinowitzvirinae* subfamily of the *Mesyanzhinovviridae* family. Of the species currently recognized by the ICTV, phage Lydia is most similar to *Pseudomonas* virus M6 (intergenomic similarity 95.9%, Figure 7), but it was difficult to confidently define its status at the species level, since the genome sequence of *Pseudomonas* virus M6 available in the NCBI database (NCBI Accessions NC_007809 and DQ163916) is incomplete, being ~2.5 kb shorter than that of phage Lydia with an interruption within a region predicted to encode an exonuclease.

#### 3.3.2. Phylogeny and Pangenome

**Figure 8:**
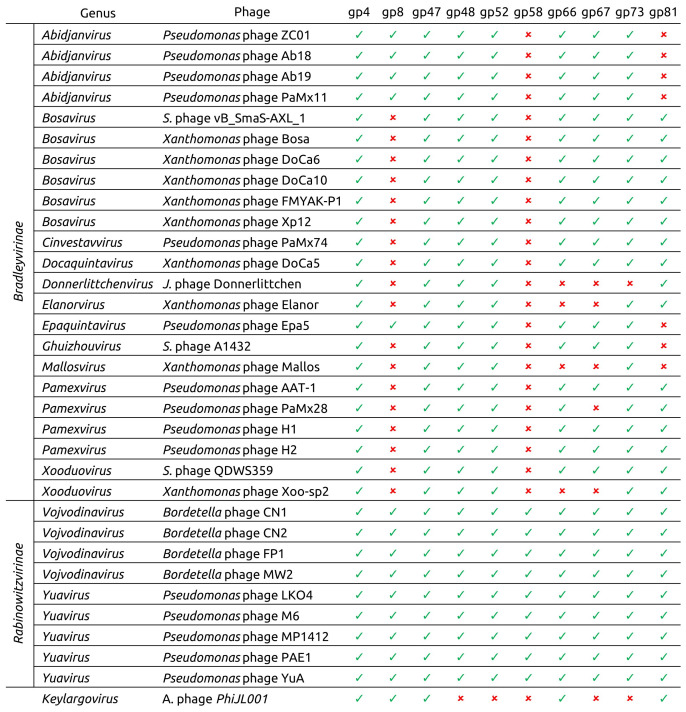
Presence of phage Lydia protein homologs in *Mesyanzhinovviridae* phages, classified by the ICTV. Gp4—DUHMT, gp8—DNAP, gp47—TLS, gp48—PP, gp52—MCP, gp58—EL, gp67—TFP, gp66 –TMP, gp73—BHP, gp81—RNRLS. Abbreviations are as follows: *A.*—*Alphaproteobacteria*, *J.*—*Janthinobacterium*, *S.*—*Stenotrophomonas*.

**Figure 9:**
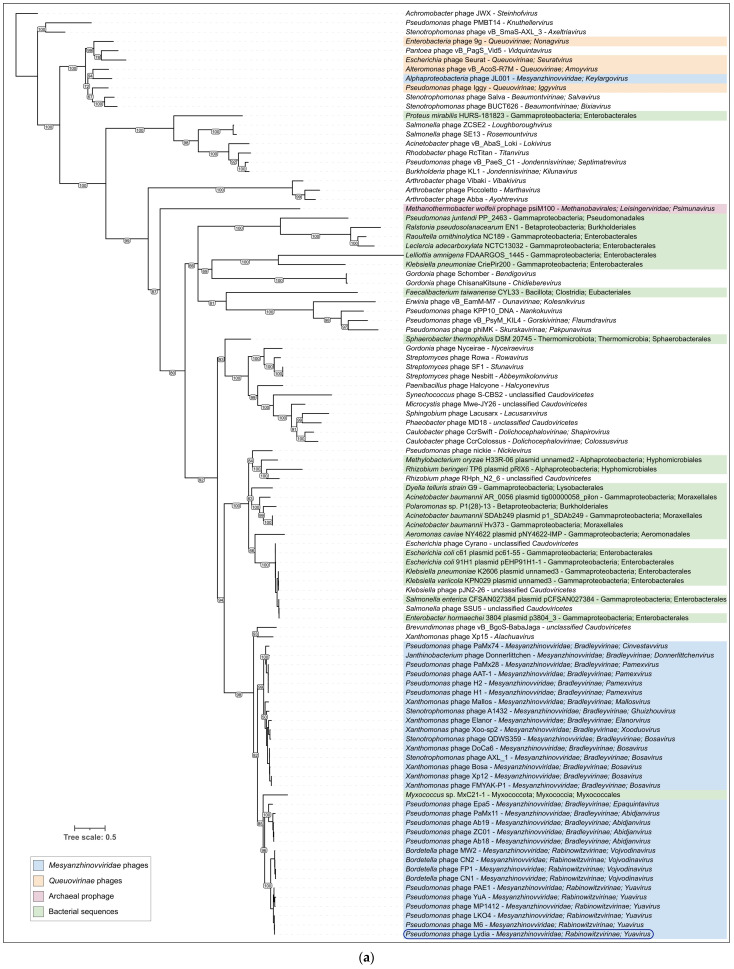

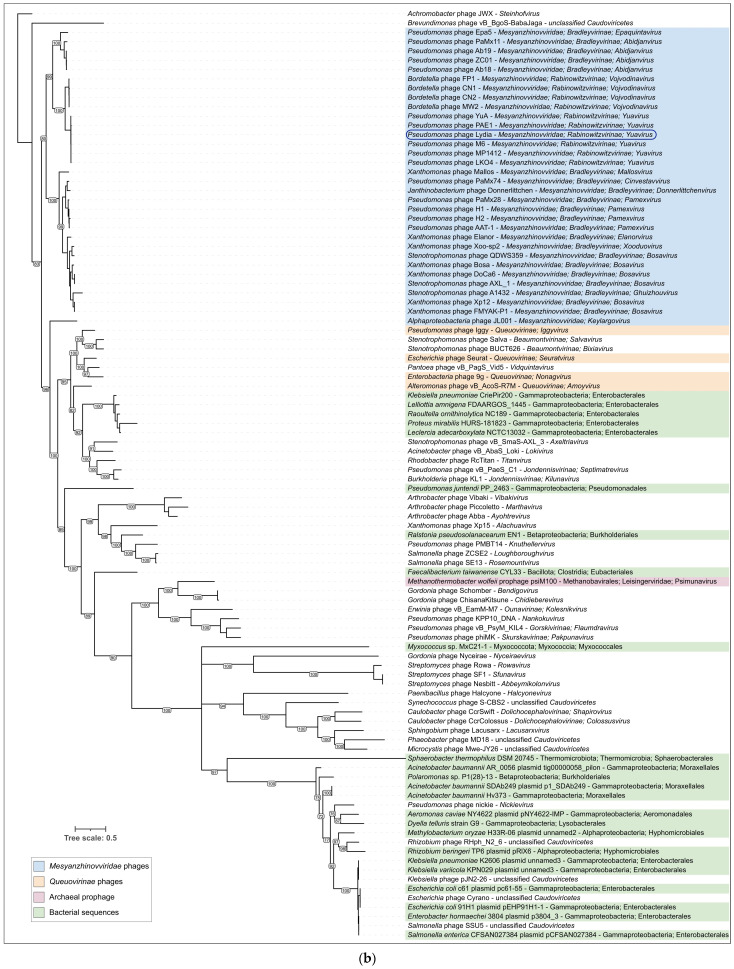
(**a**) Maximum likelihood phylogenetic trees based on amino acid sequences of proteins representing different phages and prokaryotic prophage regions. (**a**) MCP. (**b**) TLS. Taxonomy is indicated in labels and legends. Branches with a bootstrap support lower than 50% have been deleted. Bootstrap values are shown near their branches. The scale bar shows 0.5 estimated substitutions per site and the trees were rooted to *Achromobacter* phage JWX.

To explore the evolutionary relationships of phage Lydia, a TBLASTN search (E-value < 10^−5^) was conducted using the amino acid sequences of ten phage Lydia proteins as queries against a database of 33 officially classified *Mesyanzhinovviridae* phage genomes. These proteins included deoxyuridylate hydroxymethyltransferase (DUHMT, gp4), DNA polymerase (DNAP, gp8), terminase large subunit (TLS, gp47), portal protein (PP, gp48), major capsid protein (MCP, gp52), endolysin (EL, gp58), tape measure protein (TMP, gp66), tail fibre protein (TFP, gp67), baseplate hub protein (BHP, gp73), and ribonucleoside-diphosphate reductase large subunit (RNRLS, gp81). The search revealed that close homologs of some Lydia proteins are absent in phages outside the *Rabinowitzvirinae* subfamily (Figure 8). It is hypothesized that these proteins have been replaced by more distantly related counterparts via recombination and horizontal gene transfer. In some cases, this horizontal gene transfer was associated with a change in the gene’s genomic location (e.g., the DNA polymerase gene), while in other cases, the genes generally maintained their relative position within the genome (e.g., the endolysin gene). Alphaproteobacteria phage PhiJL001, which is placed as the earliest diverging member of the family based on proteomic phylogeny, exhibited the lowest similarity to the analyzed classified phages, lacking five of the ten Lydia homologs (Supplementary Figures S3 and S4, Figure 8 and Figure 9).

Phylogenetic analyses were conducted using amino acid sequences of the aforementioned proteins and the corresponding nucleotide sequences of their genes. For the protein-based phylogenetic trees (Supplementary Figure S5), sequences were obtained using phage Lydia protein sequences and BLAST searches against a custom database composed of all *Mesyanzhinovviridae* phage genomes and the GenBank PHG database (E-value < 0.05). The protein sequences from the custom database search and 20 arbitrarily chosen sequences from the PHG database were used for the phylogenetic reconstructions. The searches in the GenBank PHG database identified phages from a wide range of taxonomic groups, often distantly related, for the majority of proteins. In contrast, a search for the putative tail fibre protein (TFP, gp67) returned a significant proportion of sequences (296 out of 408) from *Pseudomonas* phages, unlike from the search results for the terminase large subunit (115 out of 505 sequences). In six out of ten cases, the protein-based phylogenetic trees demonstrated a non-monophyletic nature of *Mesyanzhinovviridae* phage proteins. Monophyly was observed only in the trees constructed for the DNA polymerase, major capsid protein, portal protein, and endolysin, but in the latter case it could be due to the exclusion of more divergent homologues of endolysin. Notably, the phylogenetic tree based on DUHMT protein sequences divided *Mesyanzhinovviridae* phages into two clades, with one being more closely related to the jumbo phage *Ralstonia* phage RSL1 (*Mieseafarmvirus*) and another one with T4-like phages (*Enterobacteria* phage vB_EcoM_VR26). Homologs of the phage Lydia DUHMT could be easily found in other large phages, including *Bacillus* phage G (*Donellivirus*) and many phages from *Ackermannviridae*, *Herrelviridae*, and *Straboviridae* families. This may suggest multiple independent origins of DUHMT or horizontal transfers between distantly related phages. Interestingly, analysis of the RNRLS and TLS revealed the presence of homologous genes in archaeal tailed viruses within the *Thumleimavirales* and *Methanobavirales* orders, respectively.

Phylogenetic analysis based on the nucleotide sequences of the genes encoding these proteins (Supplementary Figure S6) was performed using all retrieved sequences from the custom and GenBank PHG databases. These analyses of the nucleotide sequences returned a narrower range of homologous sequences, presumably due to the higher divergence rates of nucleotide sequences compared to protein sequences, but revealed the finer evolutionary patterns of recently diverged *Mesyanzhinovviridae* phages. Comparative analysis of the tree topologies revealed some differences in the *Yuavirus* genus, with the internal structure and composition of the subbranches differing. Interestingly, the *Yuavirus* branch (including phages YuA and Lydia) lacks the endolysin similar to one from *Pseudomonas* phage vB_Pa-PAC4, which was found to be more closely related to the endolysins from *Bradleyvirinae* and *Epaquintavirus* phages.

To gain a broader perspective on the evolution of the major capsid protein and terminase large subunit, an additional phylogenetic analysis was conducted using a more comprehensive set of phage sequences. This analysis involved a BLAST search against the NCBI nr database, followed by retrieval and translation of MCP and TLS gene sequences from 100 selected genomes representing various phage and bacterial groups, including officially classified phage taxa. This analysis revealed distinct evolutionary trajectories for these two proteins (Figure 9). Homologs of both MCP and TLS were found in a wide range of genomic contexts, including bacterial prophage regions, phage plasmids, archaeal viruses, and proviruses. Moreover, in contrast to the TLS tree and ViP-based proteomic tree, the *Mesyanzhinovviridae* group was not monophyletic in the MCP tree. Specifically, the MCP from the *Alphaproteobacteria* phage PhiJL001, which appears to have diverged early within the *Mesyanzhinovviridae* family, was found to be closely related to those of the *Queuovirinae* phages, whereas MCPs from the other *Mesyanzhinovviridae* phages did not show such a relationship (Figure 9a). In contrast, the TLS tree revealed a monophyletic grouping of all analyzed *Mesyanzhinovviridae* phages (Figure 9b). The presence of a *Myxococcus* sequence in the MCP tree, specifically within a clade containing *Mesyanzhinovviridae* phages infecting *Pseudomonas* (and further nested within a clade restricted to Proteobacteria-infecting phages), is highly unexpected and may be indicative of a sequencing artifact.

**Figure 10:**
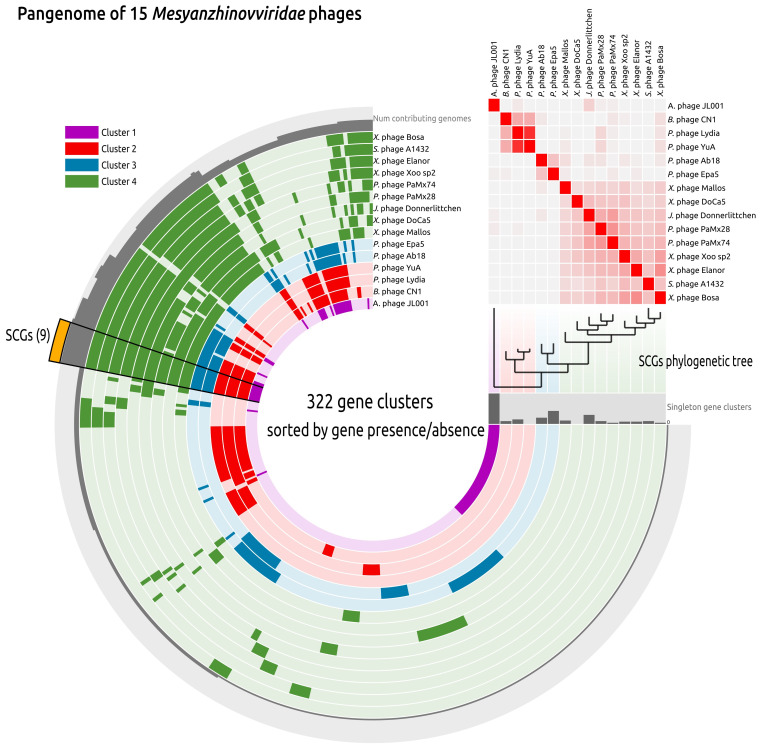
Circular visualization of the *Mesyanzhinovviridae* pangenome, including 15 genomes representing all officially classified genera and phage Lydia. Genomes are ordered based on their positions in the phylogenetic tree inferred from nine single-copy genes (SCGs). Gene clusters are shown as segments of radial layers, where the gene clusters present in genomes are coloured in intense colours corresponding to colours of phage clusters in legend, and the clusters absent in genomes are coloured in pale shades. The more intense colours on the ANI heatmap indicate higher ANI values. The radial layer “Num contributing genomes” shows the number of genomes contributing in the corresponding gene cluster. The bar chart “Singleton gene clusters” indicates the number of singleton gene clusters in the corresponding genome.

Pangenome analysis based on the genomic sequences of representative members of all *Mesyanzhinovviridae* genera, as classified by the ICTV, reinforced the divergent position of the *Alphaproteobacteria* phage PhiJL001 relative to the other members of the group (Figure 10). A phylogeny derived from concatenated alignments of nine single-copy core genes (SCGs) (similarity threshold 30%) and average nucleotide identity (ANI) calculations placed the *Keylargovirus* phage PhiJL001 outside of other groups. These analyses resolved the phage PhiJL001 and other *Mesyanzhinovviridae* phages into four distinct clusters. Cluster 1 consists only of *Alphaproteobacteria* phage PhiJL001 (*Keylargovirus* genus); cluster 2 comprises *Bordetella* phage CN1 (*Vojvodinavirus* genus) and *Pseudomonas* phages YuA and Lydia (*Yuavirus* genus); cluster 3 includes *Pseudomonas* phage Ab18 (*Abidjanvirus* genus) and *Pseudomonas* phage Epa5 (*Epaquintavirus* genus); and cluster 4 includes various phages infecting *Janthinobacterium*, *Pseudomonas*, *Stenotrophomonas*, and *Xanthomonas*, all belonging to the genera *Bosavirus*, *Cinvestavvirus*, *Docaquintavirus*, *Donnerlittchenvirus*, *Elanorvirus*, *Ghuizhouvirus*, *Mallosvirus*, *Pamexvirus*, and *Xooduovirus* (Figure 10). Phages within cluster 2 are all classified within the *Rabinowitzvirinae* subfamily, while phages from clusters 3 and 4 belong to the *Bradleyvirinae* subfamily. Unlike the topologies of the trees from MCP and TLS (Figure 10), the SCG phylogenetic tree showed the *Bradleyvirinae* subfamily as monophyletic. These clusters also differed by their characteristic patterns of gene cluster distribution and the presence of specific groups of genes, with the patterns for clusters 2 and 3 being largely similar, and distinct from those observed in cluster 4. In this analysis, phage PhiJL001 exhibits the highest proportion of unique genes relative to the other phages.

### 3.4. Search for Proteins Associated with the Temperate Lifestyle

**Figure 11:**
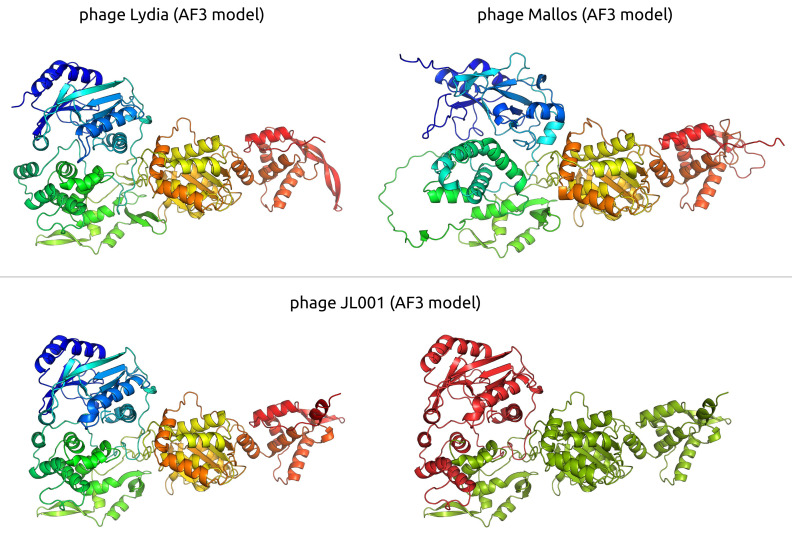
Ribbon diagrams illustrating predicted structures of putative DNA primase-helicases. **Top panel**: *Pseudomonas* phage Lydia and *Xanthomonas* phage Mallos, with structures coloured using a rainbow gradient from N-terminus (blue) to C-terminus (red). **Bottom panel**: Comparative view of the Alphaproteobacteria phage PhiJL001 protein, where the full-length AF3-modelled structure is shown with the rainbow gradient (**left**), and the *Mesyanzhinovviridae*-conserved region is highlighted in green, with the remaining portion in red (**right**).

To address conflicting reports regarding the lysogenic potential of *Mesyanzhinovviridae* phages, a sensitive HMM-based search was employed to identify putative lysogeny-associated proteins. A total of 6372 predicted proteins from 77 phage genomes attributed by NCBI to *Mesyanzhinovviridae* were analyzed using HHblits, applying an 80% probability threshold. This analysis identified two phages previously misclassified as *Mesyanzhinovviridae* possessing integrases and other lysogeny-related proteins: *Pseudomonas* phage phi_D3 (NCBI accession PP944329) and *Salmonella* phage vB_Sen_SG_WM_SEW_9 (PQ002172). No integrase, excisionase, or recombinase genes were detected in the correctly classified *Mesyanzhinovviridae* genomes. Ninety-seven proteins showed similarity to repressors and antirepressors, although this similarity extended to various transcriptional factors and DNA-binding proteins, thus lacking strong specificity for repressors. HHpred analysis across all annotated databases revealed annotation errors in previously deposited *Mesyanzhinovviridae* genomes, including the misannotation of putative DNA primase-helicase as integrase in approximately half of these genomes (e.g., Bordetella phage LK3 and *Pseudomonas* phage MP1412). This highlights the importance of the careful re-evaluation of genomic annotations, especially in the context of phage classification and infection cycle predictions. Sequence comparison, remote homology searches, and structural modelling demonstrated significant similarity between the *Mesyanzhinovviridae* primase-helicases (Figure 11). However, the N-terminal part showed greater variability than the C-terminal part, which exhibited homology across all phages listed in Table 1. One type of N-terminal part was shared among the Alphaproteobacteria phage PhiJL001 and the phages of all *Rabinowitzvirinae* and two *Bradleyvirinae* genera; a second type was found in the remaining *Bradleyvirinae* genera.

## 4. Discussion

### 4.1. Phylogenomic Relationships Within the Mesyanzhinovviridae Family

Bacteriophage evolution is a complex process driven by various factors, with horizontal gene transfer (HGT) playing a prominent role [91,92,93]. The intensity of HGT varies across viral groups, influenced by host range, lifestyle, and genome architecture; temperate phages often, but not always, exhibit higher rates of gene flux [92,94,95]. Bioinformatic analyses of *Mesyanzhinovviridae* phages reveal extensive horizontal gene transfer throughout their evolutionary history. These events frequently involved the acquisition of functionally analogous genes and gene modules while maintaining overall genome organization. Pangenomic analysis demonstrates a strong correlation between gene cluster composition, single-copy genes (SCGs) phylogeny, and the family’s taxonomy, suggesting that the emergence of *Mesyanzhinovviridae* subgroups was accompanied by genetic exchange. The divergence of Alphaproteobacteria phage PhiJL001, which possesses a major capsid protein distinct from other *Mesyanzhinovviridae* members, predates many of these HGT events, including the replacement of its endolysin gene. Topological incongruence between individual gene phylogenies suggests recombination between related phage genomes, a common feature among viruses [96] previously reported for the *Mesyanzhinovviridae Pseudomonas* phage PAE1 based on DNA sequence comparisons [33]. Furthermore, phylogenetic analysis of SCGs and conserved genes suggests the recurrent emergence of *P. aeruginosa*-infecting phage groups, likely attributable to host switching.

However, the observed incongruence in the phylogenies of separate genes within *Mesyanzhinovviridae* phages does not necessarily indicate a temperate lifestyle. A sensitive search of integrase and excisionase genes in 75 *Mesyanzhinovviridae* genomes available in GenBank’s PHG database failed to identify these genes, which are typically associated with lysogeny. The frequent misannotation of primase-helicase genes as integrases appears to stem from errors predating the sequencing of most *Mesyanzhinovviridae* phages. Often, phage annotation relies on BLAST searches and transferring annotations from homologous sequences, perpetuating earlier mistakes. While the absence of an integrase gene does not unequivocally preclude a temperate lifestyle, as some temperate phages (including tailed phages) lack integrase [97], homology searches and phylogenetic analysis using a terminase large subunit indicated a distinct, monophyletic grouping of *Mesyanzhinovviridae* with no evidence of bacterial sequences, arguing against chromosomal integration. This conclusion contrasts with a previous study reporting PCR-based confirmation of the *Bordetella* phage LK3 DNA in bacterial DNA following infection [22]. During the present study, BLAST searches of NCBI’s nt and Genome databases failed to identify homologs of LK3 major capsid protein and large terminase subunit in bacterial sequences. The LK3 gene putatively encoding integrase (gene53) was annotated based on similarity to the integrases of *Pseudomonas* phage MP1412 and *Pseudomonas* phage PAE1; however, similar to other analogous phages, HHpred analysis revealed greater similarity to primase-helicases than to bona fide integrases. Likewise, the putative repressor gene52 exhibited homology to nucleases and other viral proteins, but not to viral repressors or transcriptional regulators.

Ultimately, phage–host interactions and phage lifestyle may not be limited to a simple lytic–temperate dichotomy, as recombination and horizontal gene transfer can occur in diverse bacteria, including *B. bronchiseptica* [98] and *P. aeruginosa* [99]. Furthermore, phages can lose lysogeny-associated genes during evolution [100]. Reports of lysogeny in *Bordetella* phage LK3 [22] and the recent report of *Stenotrophomonas* phage DLP4’s ability to lysogenize *S. maltophilia* strain D1585 [18] warrant careful consideration and further investigation. Similar to previous studies of the phages YuA [28] and AN14 [36], we were unable to isolate stable, lysogenic *P. aeruginosa* PAO1 strains with the *Pseudomonas* phage Lydia.

### 4.2. Classification Issues of Phage Lydia and Related Phages

**Figure 12:**
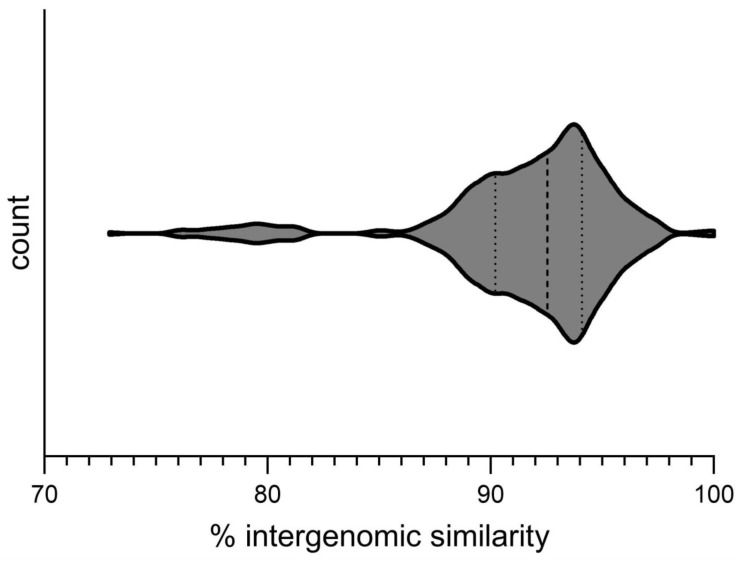
Violin diagram depicting the distribution of pairwise intergenomic similarity values among 32 phages belonging to the *Yuavirus* genus (heatmap provided in Supplementary Figure S2). The width of the violin plot is proportional to the density of data points, such that wider portions indicate higher densities and narrower portions indicate lower densities. The median and quartile values of the distribution are denoted by dotted lines.

Intergenomic comparisons and phylogenetic analyses place the *Pseudomonas* phage Lydia within the *Yuavirus* genus, closely related to *Pseudomonas* virus M6 (classified by the ICTV as *Pseudomonas* virus M6; [https://ictv.global/taxonomy, accessed 4 February 2025]). However, precise taxonomic assignment of phage Lydia (and closely related phages) requires careful consideration. Challenges arise from the incompletely sequenced genome of phage M6 and the abundance of closely related phage genomes, which exhibit a gradual rather than abrupt decline in intergenomic similarity near the ICTV’s 95% species demarcation threshold [101]. Applying this threshold to the observed distribution of intergenomic similarity values (Figure 12) results in numerous borderline cases and an excessive proliferation of new species for closely related phages. Previous work addressing taxonomic challenges in chimeric temperate phages proposed lowering the 95% species threshold [102]. However, this issue likely reflects the inherent propensity for recombination in many phages, potentially influenced by host factors. To facilitate a more practical classification scheme, a lower threshold of 85% is proposed. Further complicating consistent taxonomic classification are the incongruences observed in the evolutionary histories of hallmark proteins in tailed bacteriophages [102,103,104,105,106]. This challenge is likely intractable, and the ICTV’s approach of cautious creation of new families and higher-rank taxa appears justified.

### 4.3. Phage Lydia: Host Range, Receptors, and Therapeutic Potential

A host range analysis of phage Lydia against 30 *P. aeruginosa* strains revealed a relatively narrow lytic spectrum, despite exhibiting activity against several isolates from cystic fibrosis patients. This limited host range restricts its use in monotherapy but suggests potential applications in phage cocktails or personalized phage therapy. Bioinformatic analysis suggests that, similar to other phages infecting *Pseudomonas* spp. and other bacteria [18,28,29,35,38,79], including other *Mesyanzhinovviridae*, type IV pili serve as receptors for phage Lydia. Notably, phage Lydia’s tail fibres comprise two proteins with similar folds, a characteristic also observed in *Pseudomonas* siphovirus JBD30, not belonging to the *Mesyanzhinovviridae* family [79]. The JBD30 tail fibre protein, identified as a nitrilase superfamily hydrolase, binds to the β-sheet-rich region of the type IV major pilin protein PilA [79]. Phage Lydia’s host specificity may be further constrained by pilus glycosylation, a known *P. aeruginosa* defence mechanism [107]. Bioinformatic analyses suggest HGT events involving genes crucial for the initial stages of infection between evolutionarily and taxonomically distant *P. aeruginosa*-infecting phages, a pattern observed in other hosts [108,109].

### 4.4. Evolutionary Dynamics of Mesyanzhinovviridae Phages: Horizontal Gene Transfer and Genomic Architecture

The abundance of DNA modification proteins within the *Mesyanzhinovviridae* family likely influences infection efficiency by countering restriction–modification systems. Phylogenetic analysis of deoxyuridylate hydroxymethyltransferase, present in all analyzed *Mesyanzhinovviridae* phages, reveals a complex evolutionary history, suggesting independent acquisition in two distinct *Mesyanzhinovviridae* groups: one comprising Alphaproteobacteria phage PhiJL001, all *Rabinowitzvirinae* genera, and two *Bradleyvirinae* genera, and the other encompassing the remaining *Bradleyvirinae* genera. This non-monophyletic phylogeny further suggests possible DUHMT acquisition from two distinct lineages of large phages (including jumbo phages), which may serve as reservoirs of genes, including nucleotide-modifying enzymes, for other phages. The observed relatedness of *Mesyanzhinovviridae* capsid and tail proteins to those of the *Rhodobacter capsulatus* GTA particle further supports the hypothesis of a phage origin for these virus-like particles [110]. Moreover, the significant similarity between phage Lydia’s major capsid protein and Family 2A encapsulins involved in sulfur metabolism raises questions about the evolutionary relationship between encapsulins and phages. This similarity, absent in the Alphaproteobacteria phage PhiJL001, likely reflects genetic exchange involving the major capsid protein (MCP) gene during *Mesyanzhinovviridae* evolution. Overall, our findings highlight the versatility of phage genomes in acquiring genes while maintaining functional conservation and overall genomic architecture. As a fundamental form of organizational information—alongside gene sequences, protein structures, and regulatory elements—genomic architecture provides key insights into phage life cycle processes and may prove useful in characterizing higher-order taxa in cases of conflicting evolutionary histories of conserved proteins, as previously suggested [102].

## Figures and Tables

**Table 1:** Approved by the ICTV classification scheme of *Mesyanzhinovviridae* phages (according to the ICTV Master Species List 2023_MSL39.v4).

Subfamily	Genus	Species	Phage Name	NCBI Accession	Short Phage Name
*Bradleyvirinae*	*Abidjanvirus*	*Abidjanvirus ZC01*	*Pseudomonas* phage ZC01 [38,39]	KU356689	
*Bradleyvirinae*	*Abidjanvirus*	*Pseudomonas virus Ab18*	*Pseudomonas* phage vB_PaeS_PAO1_Ab18	LN610577	*Pseudomonas* phage *Ab18*
*Bradleyvirinae*	*Abidjanvirus*	*Pseudomonas virus Ab19*	*Pseudomonas* phage vB_PaeS_PAO1_Ab19	LN610584	*Pseudomonas* phage *Ab19*
*Bradleyvirinae*	*Abidjanvirus*	*Pseudomonas virus PaMx11*	*Pseudomonas* phage PaMx11 [27]	JQ067087	
*Bradleyvirinae*	*Bosavirus*	*Bosavirus Axl1*	*Stenotrophomonas* phage vB_SmaS-AXL_1 [35]	OL674541	
*Bradleyvirinae*	*Bosavirus*	*Bosavirus bosa*	*Xanthomonas* phage Bosa	LR743532	
*Bradleyvirinae*	*Bosavirus*	*Bosavirus Doca6*	*Xanthomonas* phage vB_Xar_IVIA-DoCa6 [20]	ON932080	*Xanthomonas* phage DoCa6
*Bradleyvirinae*	*Bosavirus*	*Bosavirus Doca10*	*Xanthomonas* phage vB_Xar_IVIA-DoCa10 [20]	ON932084	*Xanthomonas* phage DoCa10
*Bradleyvirinae*	*Bosavirus*	*Bosavirus FMYAK*	*Xanthomonas* phage FMYAK-P1	OK275492	
*Bradleyvirinae*	*Bosavirus*	*Bosavirus Xp12*	*Xanthomonas* phage Xp12 [26,32,86,87]	MT664984	
*Bradleyvirinae*	*Cinvestavvirus*	*Cinvestavvirus PaMx74*	*Pseudomonas* phage PaMx74 [88]	JQ067093	
*Bradleyvirinae*	*Docaquintavirus*	*Docaquintavirus doca5*	*Xanthomonas* phage vB_Xar_IVIA-DoCa5 [20]	ON932079	*Xanthomonas* phage DoCa5
*Bradleyvirinae*	*Donnerlittchenvirus*	*Donnerlittchenvirus donnerlittchenvirus*	*Janthinobacterium* phagevB_JliM-Donnerlittchen [89]	ON529854	*Janthinobacterium* phage Donnerlittchen
*Bradleyvirinae*	*Elanorvirus*	*Elanorvirus elanor*	*Xanthomonas* phage Elanor [19]	ON189045	
*Bradleyvirinae*	*Epaquintavirus*	*Epaquintavirus Epa5*	*Pseudomonas* phage Epa5 [40]	MT108725	
*Bradleyvirinae*	*Ghuizhouvirus*	*Ghuizhouvirus A1432*	*Stenotrophomonas* phage A1432 [30]	ON005621	
*Bradleyvirinae*	*Mallosvirus*	*Mallosvirus mallos*	*Xanthomonas* phage Mallos [19]	ON189047	
*Bradleyvirinae*	*Pamexvirus*	*Pamexvirus AAT1*	*Pseudomonas* phage AAT-1 [37]	KU204984	
*Bradleyvirinae*	*Pamexvirus*	*Pamexvirus PaMx28*	*Pseudomonas* phage PaMx28	JQ067089	
*Bradleyvirinae*	*Pamexvirus*	*Pamexvirus phiH1*	*Pseudomonas* phage phiH1	OP380269	*Pseudomonas* phage H1
*Bradleyvirinae*	*Pamexvirus*	*Pamexvirus phiH2*	*Pseudomonas* phage phiH2	OP361299	*Pseudomonas* phage H2
*Bradleyvirinae*	*Xooduovirus*	*Xooduovirus QDWS359*	*Stenotrophomonas* phage vB_Sm_QDWS359	ON331942	*Stenotrophomonas* phage QDWS359
*Bradleyvirinae*	*Xooduovirus*	*Xooduovirus Xoosp2*	*Xanthomonas* phage Xoo-sp2 [21]	KX241618	
*Rabinowitzvirinae*	*Vojvodinavirus*	*Bordetella virus CN1*	*Bordetella* phage CN1 [22]	KY000221	
*Rabinowitzvirinae*	*Vojvodinavirus*	*Bordetella virus CN2*	*Bordetella* phage CN2 [22]	KY000219	
*Rabinowitzvirinae*	*Vojvodinavirus*	*Bordetella virus FP1*	*Bordetella* phage FP1 [22]	KY000220	
*Rabinowitzvirinae*	*Vojvodinavirus*	*Bordetella virus MW2*	*Bordetella* phage MW2 [22]	KY000218	
*Rabinowitzvirinae*	*Yuavirus*	*Pseudomonas virus LKO4*	*Pseudomonas* phage LKO4	KC758116	
*Rabinowitzvirinae*	*Yuavirus*	*Pseudomonas virus M6*	*Pseudomonas* phage M6	DQ163916	
*Rabinowitzvirinae*	*Yuavirus*	*Pseudomonas virus MP1412*	*Pseudomonas* phage MP1412 [90]	JX131330	
*Rabinowitzvirinae*	*Yuavirus*	*Pseudomonas virus PAE1*	*Pseudomonas* phage PAE1 [33]	KT734862	
*Rabinowitzvirinae*	*Yuavirus*	*Pseudomonas virus Yua*	*Pseudomonas* phage YuA [28]	AM749441	
	*Keylargovirus*	*Keylargovirus JL001*	Alphaproteobacteria phage PhiJL001 [17]	AY576273	Alphaproteobacteria phage JL001

## Data Availability

All relevant data are available within this article and its Supplementary Materials. Additional data, including those removed from the Supplementary Materials, can be obtained from the authors upon reasonable request. The *Pseudomonas* phage Lydia genome sequence was deposited in NCBI GenBank under accession number PV031332.

## Supplementary Materials

The following supporting information can be downloaded at: https://www.mdpi.com/article/10.3390/v17030369/s1, Figure S1: Clear plaque fashioned by *Pseudomonas* phage Lydia with *Pseudomonas aeruginosa* PAO1 host lawn on a double-layer agar plate. Figure S2: VIRIDIC heatmap generated using genomic sequences found with BLAST search using the MCP and TLS amino acid sequences of *Pseudomonas* phage Lydia. Abbreviations are as follows: Bp—*Bordetella* phage, Jp—*Janthinobacterium* phage, Pp—*Pseudomonas* phage, Pxp—*Pseudoxanthomonas* phage, Spp—*Sphaerotilus* phage, Sp—*Stenotrophomonas* phage. Figure S3: Fragment of ViPtree proteomic tree. Cluster of *Mesyanzhinovviridae* phages is outlined in red. Figure S4: Comparative genome alignment of *Mesyanzhinovviridae* phages (*Pseudomonas* phage Lydia, *Pseudomonas* phage M6, *Bordetella* phage CN1, *Pseudomonas* phage ZC01, Alphaproteobacteria phage PhiJL001, *Pseudomonas* phage PaMx28, *Janthinobacterium* phage vB_JliM-Donnerlittchen, *Xanthomonas* phage Bosa. Figure S5: Maximum likelihood phylogenetic trees based on amino acid sequences of phage proteins. The scale bar shows the number of estimated substitutions per site. Taxonomy is indicated in labels and legends. Branches with a bootstrap support lower than 50% have been deleted. Bootstrap values are shown near their branches. (**a**) DUHMT (gp4); the tree was rooted to *Bacillus* phage G. (**b**) DNAP (gp8); the tree was unrooted. (**c**) TLS (gp47); the tree was unrooted. (**d**) PP (gp48); the tree was unrooted. (**e**) MCP (gp52); the tree was rooted to *Bacillus* phage PK2. (**f**) EL (gp58); the tree was unrooted. (**g**) TMP (gp66); the tree was unrooted. (**h**) TFP (gp67); the tree was rooted to the midpoint. (**i**) BHP (gp73); the tree was rooted to the midpoint. (**j**) RNRLS (gp81); the tree was rooted to *Haloarcula californiae* tailed virus 1. Figure S6. Maximum likelihood phylogenetic trees based on nucleotide sequences of predicted phage genes. The scale bar shows the number of estimated substitutions per site. Taxonomy is indicated in labels and legends. Branches with a bootstrap support lower than 50% have been deleted. Bootstrap values are shown near their branches. The trees were unrooted. (**a**) DUHMT (gene4). (**b**) TLS (gene47). (**c**) MCP (gene52). (**d**) EL (gene58). Table S1: Sensitivity of *Pseudomonas aeruginosa* clinical isolates to *Pseudomonas* phage Lydia (ST—seqence type determined using PubMLST). File S1: Annotated sequence of *Pseudomonas* phage Lydia. File S2: Best-ranking AF3 models of phage Lydia proteins.
