# Supplementary material for: Pseudomonas Phage Lydia and the Evolution of the Mesyanzhinovviridae Family
Source: Viruses. 2025 Mar 4;17(3):369. doi: 10.3390/v17030369 (PMC11946847; doi:10.3390/v17030369)
Supplement: Supplementary file 1 [file viruses-17-00369-s001.zip › Supplementary_Materials.pdf]

viruses

MDPI

Article

Supplementary Materials

Table S1. Sensitivity of *Pseudomonas aeruginosa* clinical isolates to *Pseudomonas* phage Lydia (ST – sequence type determined using PubMLST).

P. aeruginosa Strain and Clinical Isolate	ST	Sensitivity to Bacteriophage (++ Pronounced Ly-sis, + Ly-sis and – No Lysis Ob-served)
PAO1 laboratory Krylov	549	++
PAO1	549	-
97-1	358	-
99-1	942	++
158-4	2465	-
189-1	274	++
220/2	274	++
233-2	245	+
294	16	-
105/4	200	++
17902-1	635	-
17905-1	2592	-
17911-0	1205	-
17(2)	231	-
62(1)	3496	-
80/2	233	-
82/2	254	-
82/2_1		-
98/3	235	-
128/2	235	-
215/4	308	-
239/1	242	-
294 I		-
294 I_1		-
294 II	1203	-
316 I	235	-
316 II	235	-
317	235	+
325 I	12	-
17903	395	-
41748/2	235	-

[1] Tables may have a footer.

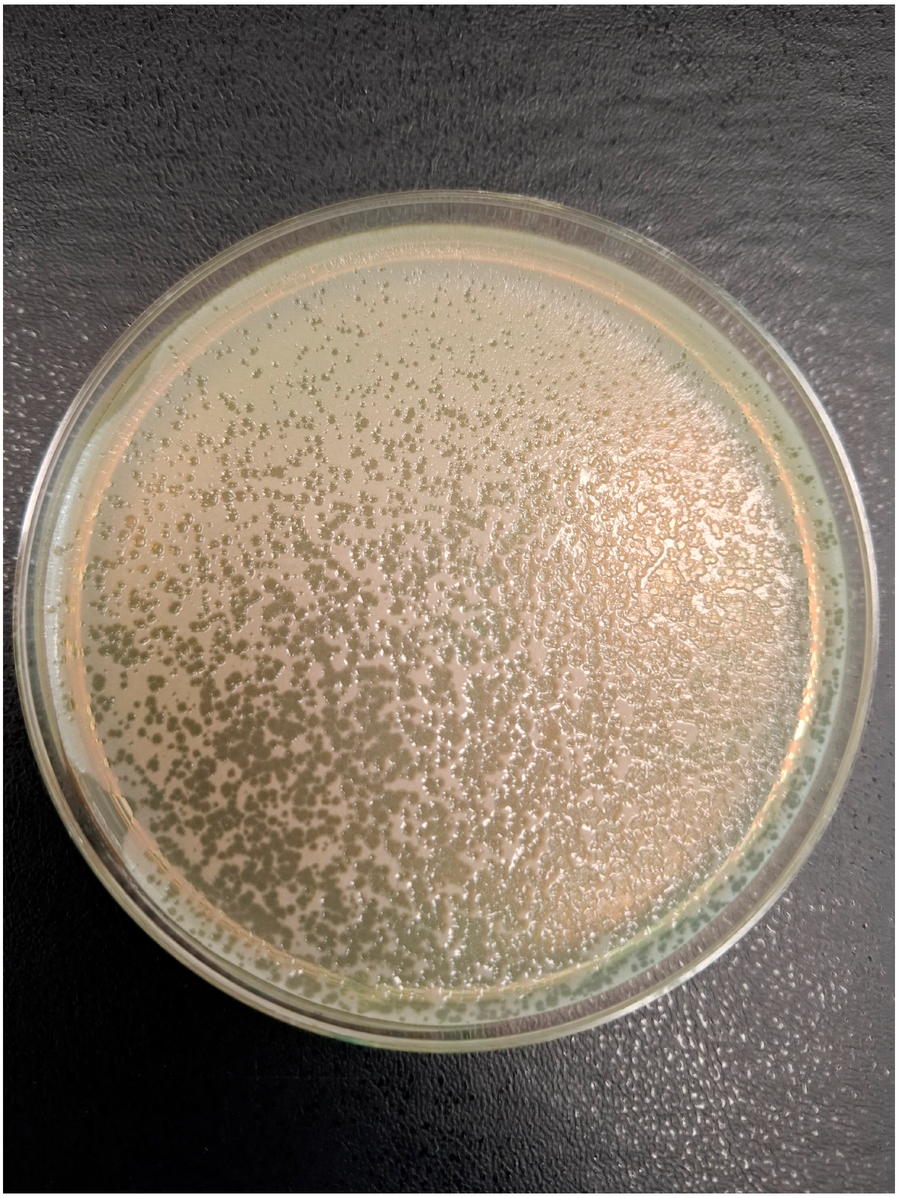

Supplementary Figure S1. Clear plaque fashioned by *Pseudomonas* phage Lydia with *Pseudomonas aeruginosa* PAO1 host lawn on a double-layer agar plate.

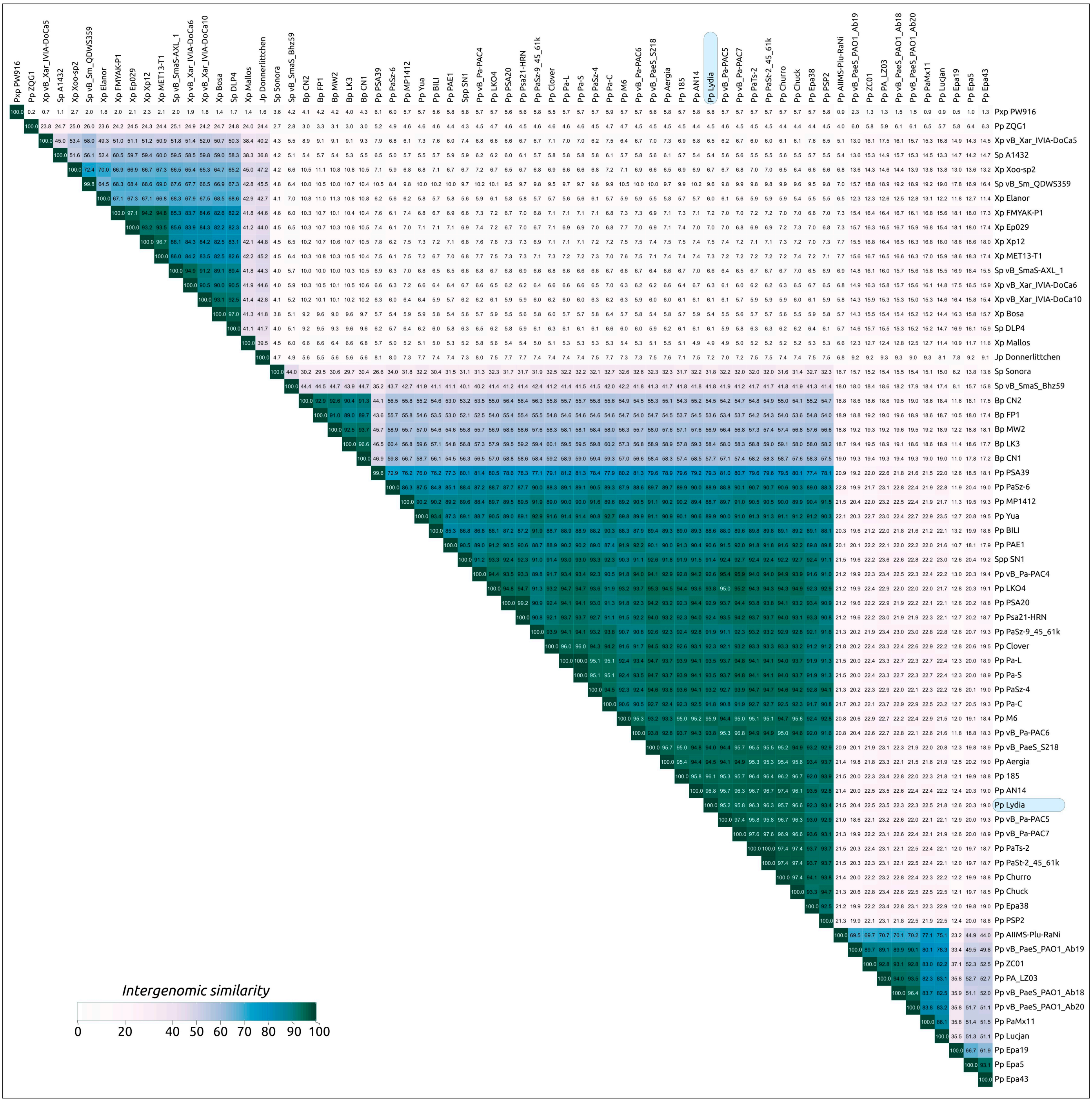

Supplementary Figure S2. VIRIDIC heatmap generated using genomic sequences found with BLAST search using the MCP and TLS amino acid sequences of *Pseudomonas* phage Lydia. Abbreviations are as follows: Bp – *Bordetella* phage, Jp – *Janthinobacterium* phage, Pp – *Pseudomonas* phage, Pxp – *Pseudoxanthomonas* phage, Spp – *Sphaerotilus* phage, Sp – *Stenotrophomonas* phage.

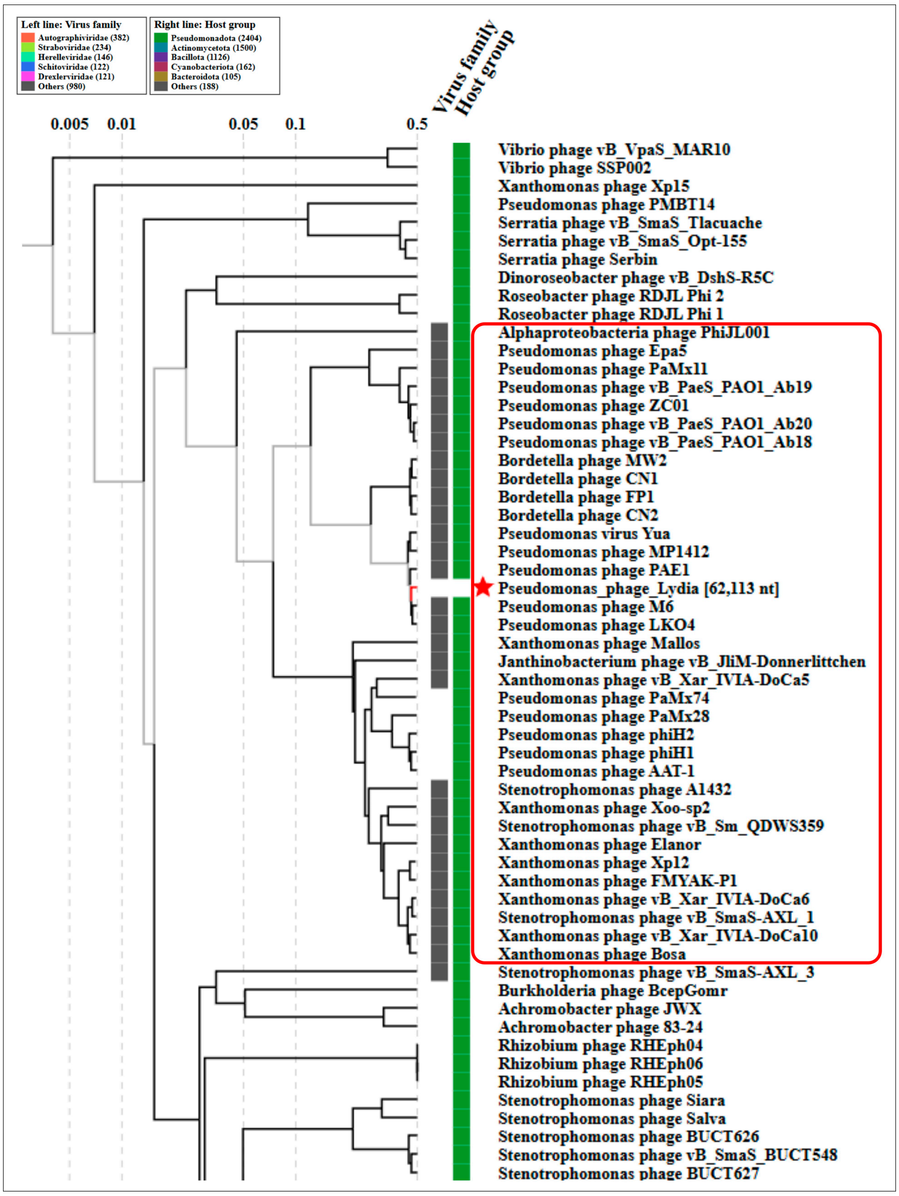

Supplementary Figure S3. Fragment of ViPtree proteomic tree. Cluster of *Mesyanzhinovviridae* phages is outlined red.

viruses
MDPI

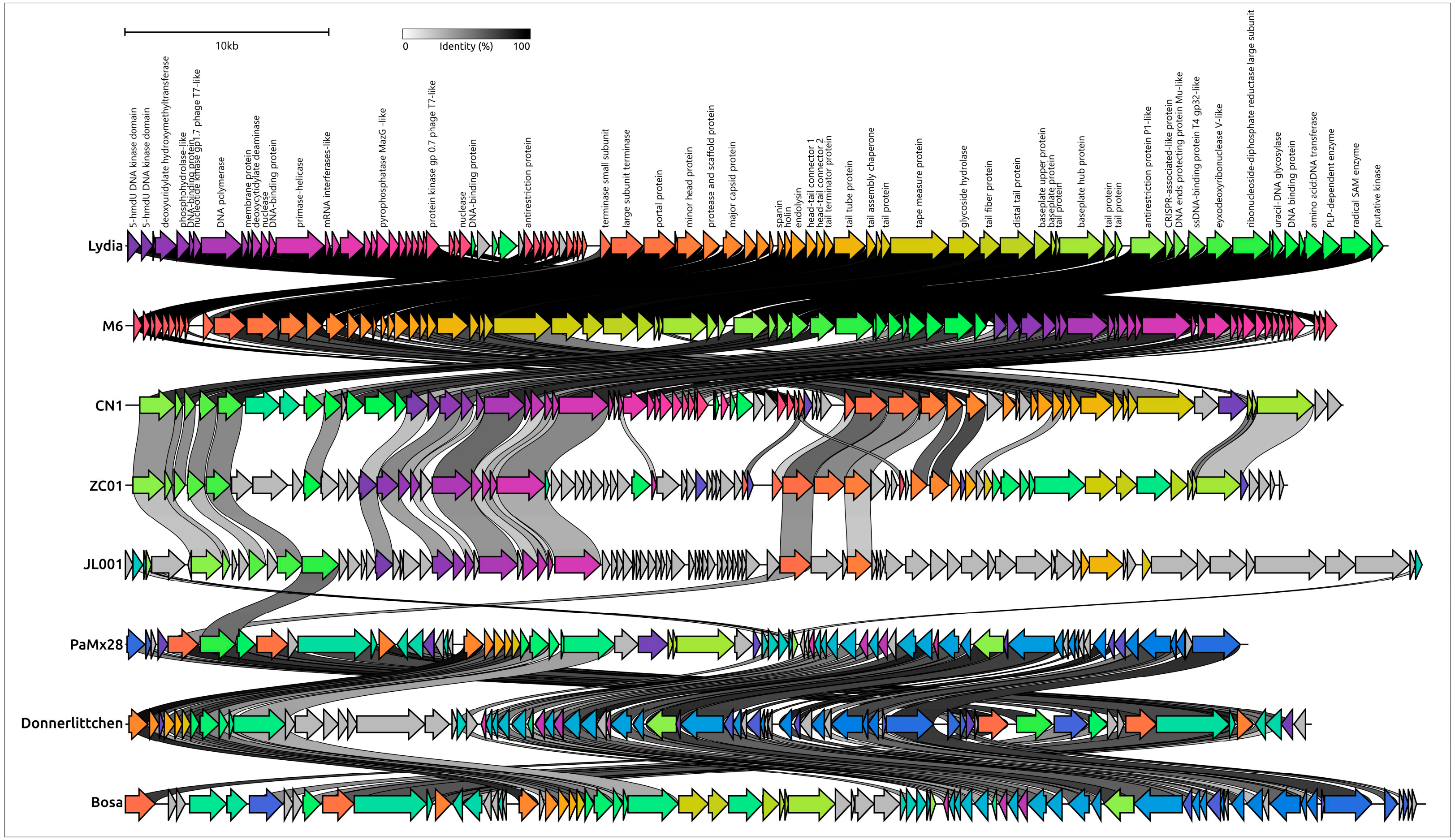

Supplementary Figure S4. Comparative genome alignment of *Mesyanzhinovviridae* phages (*Pseudomonas* phage Lydia, *Pseudomonas* phage M6, *Bordetella* phage CN1, *Pseudomonas* phage ZC01, Alphaproteobacteria phage PhiJL001, *Pseudomonas* phage PaMx28, *Janthinobacterium* phage vB_JliM-Donnerlittchen, *Xanthomonas* phage Bosa.

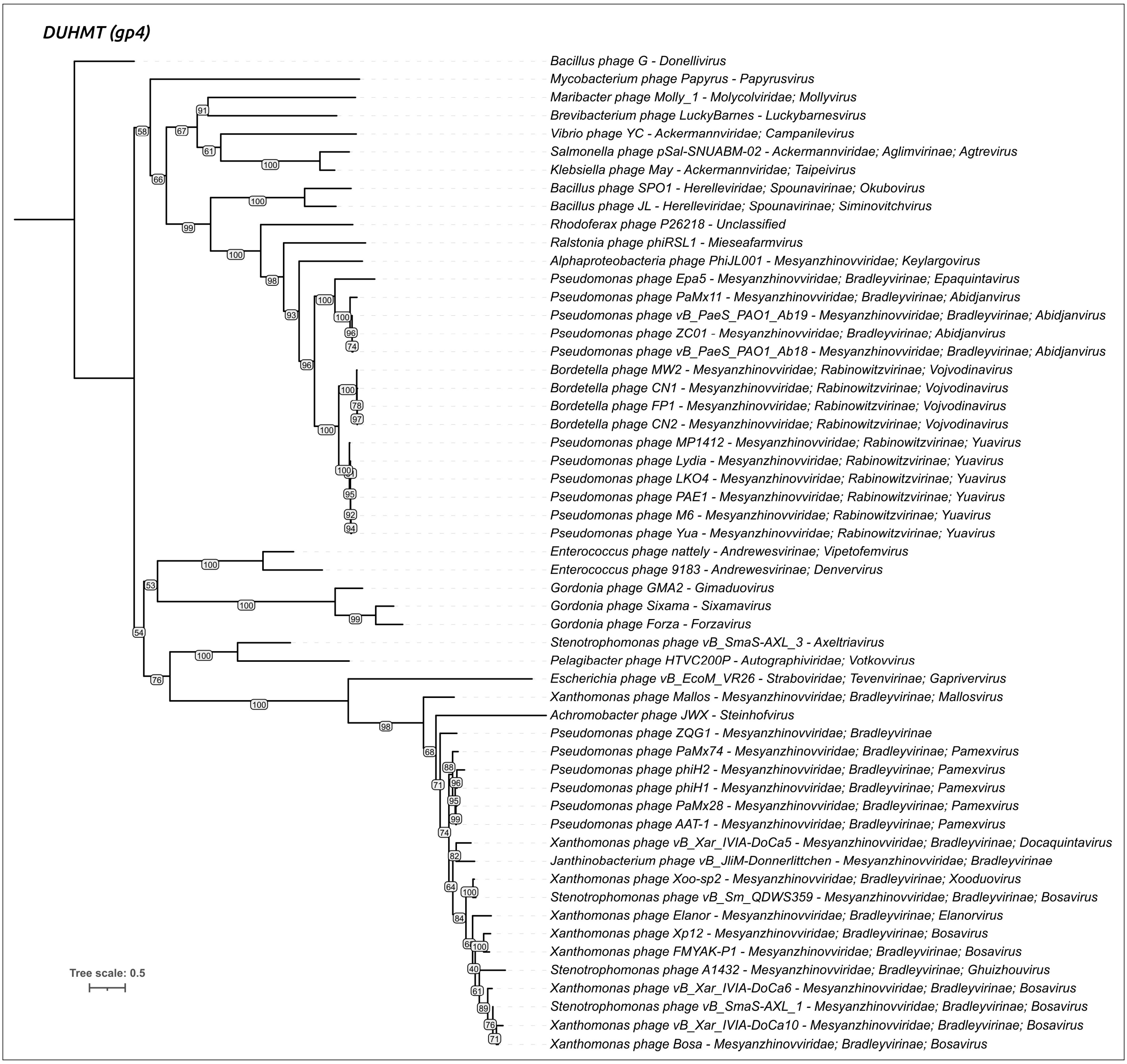
DUHMT (gp4)
Bacillus phage G - Donellivirus
Mycobacterium phage Papyrus - Papyrusvirus
Maribacter phage Molly_1 - Molycolviridae; Mollyvirus
Brevibacterium phage LuckyBarnes - Luckybarnesvirus
Vibrio phage YC - Ackermannviridae; Campanilevirus
Salmonella phage pSal-SNUABM-02 - Ackermannviridae; Aglimvirinae; Agtrevirus
Klebsiella phage May - Ackermannviridae; Taipeivirus
Bacillus phage SPO1 - Herelleviridae; Spounavirinae; Okubovirus
Bacillus phage JL - Herelleviridae; Spounavirinae; Siminovitchvirus
Rhodoferax phage P26218 - Unclassified
Ralstonia phage phiRSL1 - Mieseafarmvirus
Alphaproteobacteria phage PhiJL001 - Mesyanzhinovviridae; Keylargovirus
Pseudomonas phage Epa5 - Mesyanzhinovviridae; Bradleyvirinae; Epaquintavirus
Pseudomonas phage PaMx11 - Mesyanzhinovviridae; Bradleyvirinae; Abidjanvirus
Pseudomonas phage vB_PaeS_PAO1_Ab19 - Mesyanzhinovviridae; Bradleyvirinae; Abidjanvirus
Pseudomonas phage ZC01 - Mesyanzhinovviridae; Bradleyvirinae; Abidjanvirus
Pseudomonas phage vB_PaeS_PAO1_Ab18 - Mesyanzhinovviridae; Bradleyvirinae; Abidjanvirus
Bordetella phage MW2 - Mesyanzhinovviridae; Rabinowitzvirinae; Vojvodinavirus
Bordetella phage CN1 - Mesyanzhinovviridae; Rabinowitzvirinae; Vojvodinavirus
Bordetella phage FP1 - Mesyanzhinovviridae; Rabinowitzvirinae; Vojvodinavirus
Bordetella phage CN2 - Mesyanzhinovviridae; Rabinowitzvirinae; Vojvodinavirus
Pseudomonas phage MP1412 - Mesyanzhinovviridae; Rabinowitzvirinae; Yuavirus
Pseudomonas phage Lydia - Mesyanzhinovviridae; Rabinowitzvirinae; Yuavirus
Pseudomonas phage LKO4 - Mesyanzhinovviridae; Rabinowitzvirinae; Yuavirus
Pseudomonas phage PAE1 - Mesyanzhinovviridae; Rabinowitzvirinae; Yuavirus
Pseudomonas phage M6 - Mesyanzhinovviridae; Rabinowitzvirinae; Yuavirus
Pseudomonas phage Yua - Mesyanzhinovviridae; Rabinowitzvirinae; Yuavirus
Enterococcus phage nattely - Andrewesvirinae; Vipetofemvirus
Enterococcus phage 9183 - Andrewesvirinae; Denvervirus
Gordonia phage GMA2 - Gimaduovirus
Gordonia phage Sixama - Sixamavirus
Gordonia phage Forza - Forzavirus
Stenotrophomonas phage vB_SmaS-AXL_3 - Axeltriavirus
Pelagibacter phage HTVC200P - Autographiviridae; Votkovvirus
Escherichia phage vB_EcoM_VR26 - Straboviridae; Tevenvirinae; Gaprivervirus
Xanthomonas phage Mallos - Mesyanzhinovviridae; Bradleyvirinae; Mallosvirus
Achromobacter phage JWX - Steinhofvirus
Pseudomonas phage ZQG1 - Mesyanzhinovviridae; Bradleyvirinae
Pseudomonas phage PaMx74 - Mesyanzhinovviridae; Bradleyvirinae; Pamexvirus
Pseudomonas phage phiH2 - Mesyanzhinovviridae; Bradleyvirinae; Pamexvirus
Pseudomonas phage phiH1 - Mesyanzhinovviridae; Bradleyvirinae; Pamexvirus
Pseudomonas phage PaMx28 - Mesyanzhinovviridae; Bradleyvirinae; Pamexvirus
Pseudomonas phage AAT-1 - Mesyanzhinovviridae; Bradleyvirinae; Pamexvirus
Xanthomonas phage vB_Xar_IVIA-DoCa5 - Mesyanzhinovviridae; Bradleyvirinae; Docaquintavirus
Janthinobacterium phage vB_JliM-Donnerlittchen - Mesyanzhinovviridae; Bradleyvirinae
Xanthomonas phage Xoo-sp2 - Mesyanzhinovviridae; Bradleyvirinae; Xooduovirus
Stenotrophomonas phage vB_Sm_QDWS359 - Mesyanzhinovviridae; Bradleyvirinae; Bosavirus
Xanthomonas phage Elanor - Mesyanzhinovviridae; Bradleyvirinae; Elanorvirus
Xanthomonas phage Xp12 - Mesyanzhinovviridae; Bradleyvirinae; Bosavirus
Xanthomonas phage FMYAK-P1 - Mesyanzhinovviridae; Bradleyvirinae; Bosavirus
Stenotrophomonas phage A1432 - Mesyanzhinovviridae; Bradleyvirinae; Ghuizhouvirus
Xanthomonas phage vB_Xar_IVIA-DoCa6 - Mesyanzhinovviridae; Bradleyvirinae; Bosavirus
Stenotrophomonas phage vB_SmaS-AXL_1 - Mesyanzhinovviridae; Bradleyvirinae; Bosavirus
Xanthomonas phage vB_Xar_IVIA-DoCa10 - Mesyanzhinovviridae; Bradleyvirinae; Bosavirus
Xanthomonas phage Bosa - Mesyanzhinovviridae; Bradleyvirinae; Bosavirus
Tree scale: 0.5

(a)

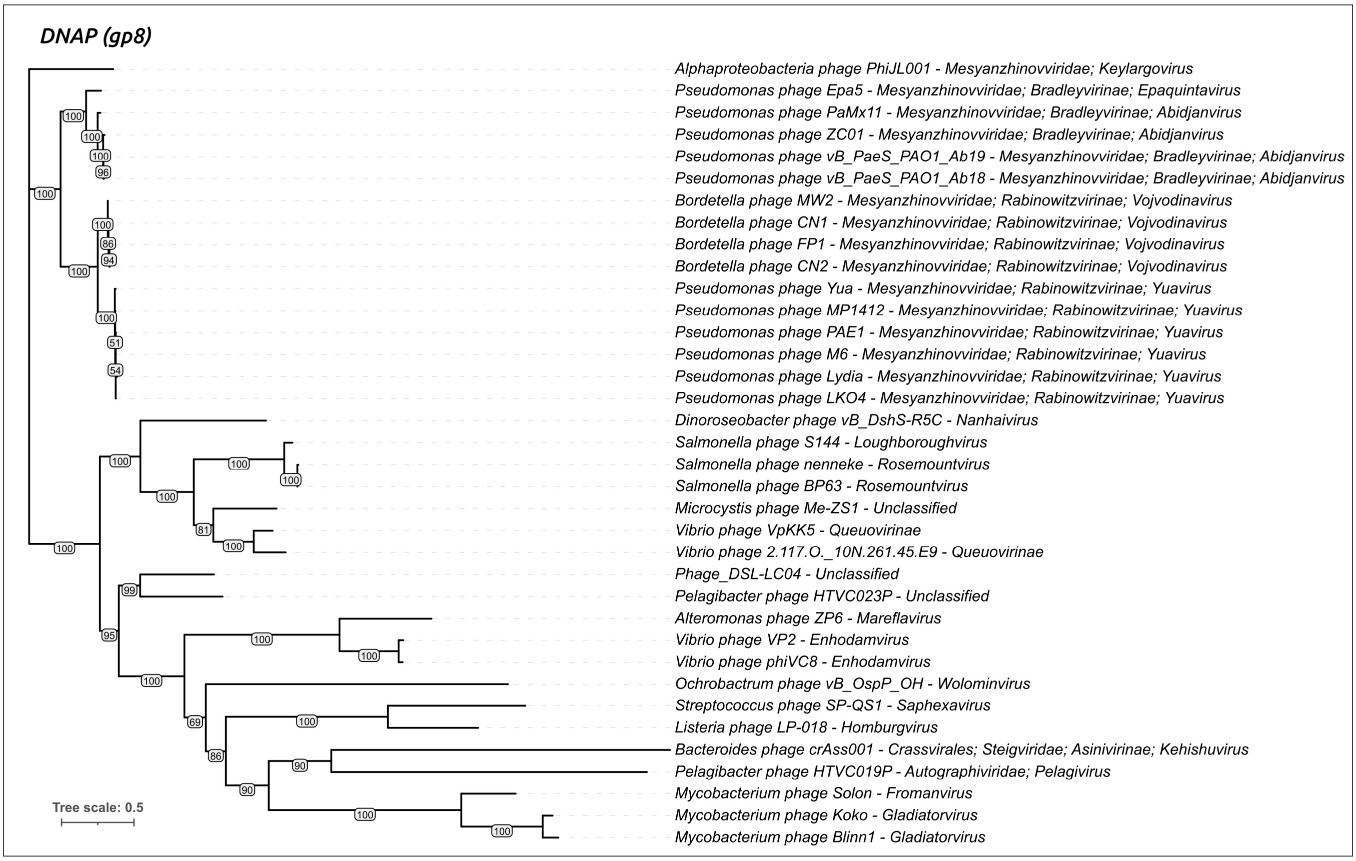
DNAP (gp8)
Alphaproteobacteria phage PhiJL001 - Mesyanzhinovviridae; Keylargovirus
Pseudomonas phage Epa5 - Mesyanzhinovviridae; Bradleyvirinae; Epaquintavirus
Pseudomonas phage PaMx11 - Mesyanzhinovviridae; Bradleyvirinae; Abidjanvirus
Pseudomonas phage ZC01 - Mesyanzhinovviridae; Bradleyvirinae; Abidjanvirus
Pseudomonas phage vB_PaeS_PAO1_Ab19 - Mesyanzhinovviridae; Bradleyvirinae; Abidjanvirus
Pseudomonas phage vB_PaeS_PAO1_Ab18 - Mesyanzhinovviridae; Bradleyvirinae; Abidjanvirus
Bordetella phage MW2 - Mesyanzhinovviridae; Rabinowitzvirinae; Vojvodinavirus
Bordetella phage CN1 - Mesyanzhinovviridae; Rabinowitzvirinae; Vojvodinavirus
Bordetella phage FP1 - Mesyanzhinovviridae; Rabinowitzvirinae; Vojvodinavirus
Bordetella phage CN2 - Mesyanzhinovviridae; Rabinowitzvirinae; Vojvodinavirus
Pseudomonas phage Yua - Mesyanzhinovviridae; Rabinowitzvirinae; Yuavirus
Pseudomonas phage MP1412 - Mesyanzhinovviridae; Rabinowitzvirinae; Yuavirus
Pseudomonas phage PAE1 - Mesyanzhinovviridae; Rabinowitzvirinae; Yuavirus
Pseudomonas phage M6 - Mesyanzhinovviridae; Rabinowitzvirinae; Yuavirus
Pseudomonas phage Lydia - Mesyanzhinovviridae; Rabinowitzvirinae; Yuavirus
Pseudomonas phage LKO4 - Mesyanzhinovviridae; Rabinowitzvirinae; Yuavirus
Dinoroseobacter phage vB_DshS-R5C - Nanhaivirus
Salmonella phage S144 - Loughboroughvirus
Salmonella phage nenneke - Rosemountvirus
Salmonella phage BP63 - Rosemountvirus
Microcystis phage Me-ZS1 - Unclassified
Vibrio phage VpKK5 - Queuovirinae
Vibrio phage 2.117.O._10N.261.45.E9 - Queuovirinae
Phage_DSL-LC04 - Unclassified
Pelagibacter phage HTVC023P - Unclassified
Alteromonas phage ZP6 - Mareflavirus
Vibrio phage VP2 - Enhodamvirus
Vibrio phage phiVC8 - Enhodamvirus
Ochrobactrum phage vB_OspP_OH - Wolominvirus
Streptococcus phage SP-QS1 - Saphexavirus
Listeria phage LP-018 - Homburgvirus
Bacteroides phage crAss001 - Crassvirales; Steigviridae; Asinivirinae; Kehishuvirus
Pelagibacter phage HTVC019P - Autographiviridae; Pelagivirus
Mycobacterium phage Solon - Fromanvirus
Mycobacterium phage Koko - Gladiatorvirus
Mycobacterium phage Blinn1 - Gladiatorvirus
Tree scale: 0.5

(b)

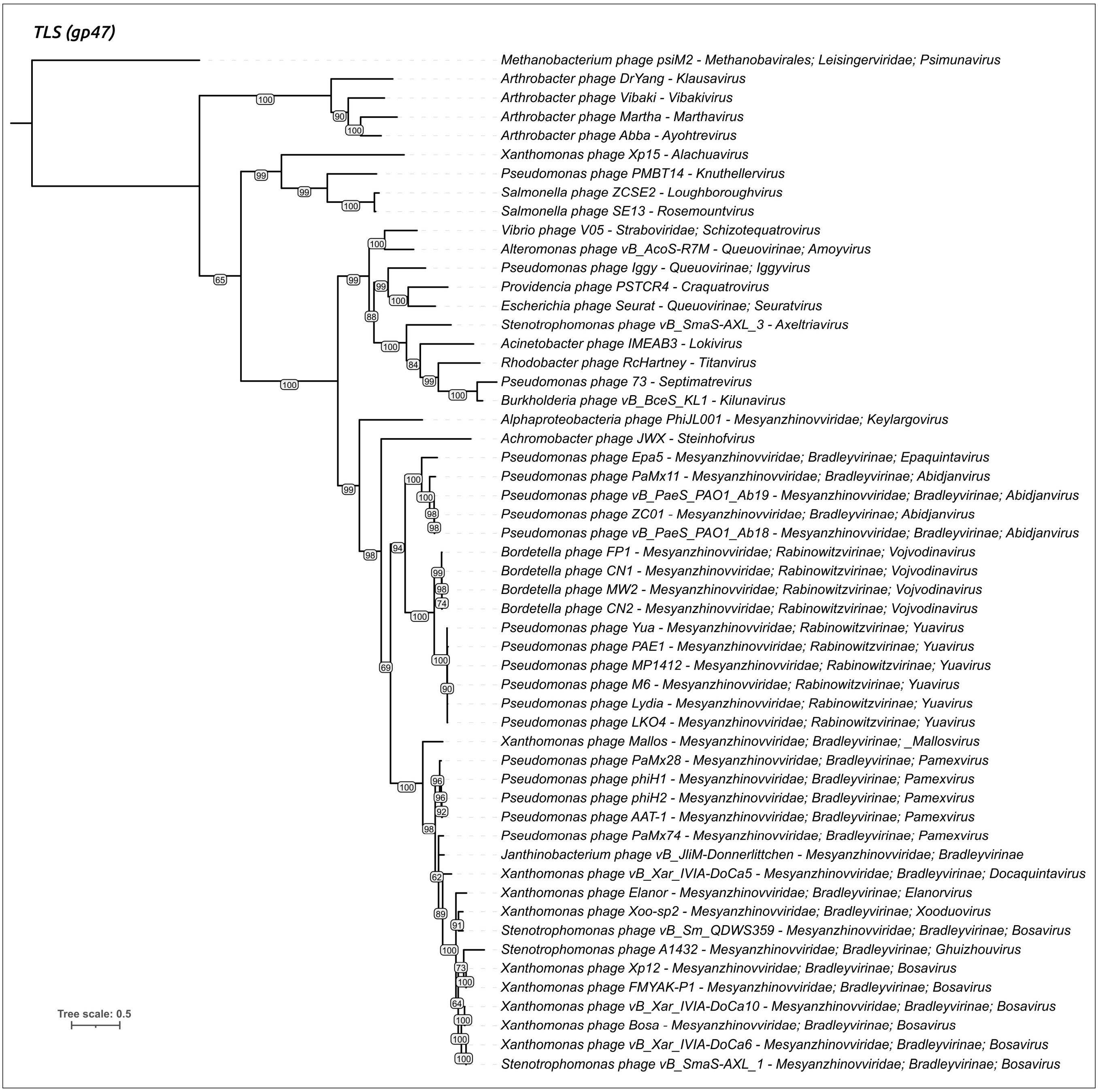
TLS (gp47)
Methanobacterium phage psiM2 - Methanobavirales; Leisingerviridae; Psimunavirus
Arthrobacter phage DrYang - Klausavirus
Arthrobacter phage Vibaki - Vibakivirus
Arthrobacter phage Martha - Marthavirus
Arthrobacter phage Abba - Ayohtrevirus
Xanthomonas phage Xp15 - Alachuavirus
Pseudomonas phage PMBT14 - Knuthellervirus
Salmonella phage ZCSE2 - Loughboroughvirus
Salmonella phage SE13 - Rosemountvirus
Vibrio phage V05 - Straboviridae; Schizotequatrovirus
Alteromonas phage vB_AcoS-R7M - Queuovirinae; Amoyvirus
Pseudomonas phage Iggy - Queuovirinae; Iggyvirus
Providencia phage PSTCR4 - Craquatrovirus
Escherichia phage Seurat - Queuovirinae; Seuratvirus
Stenotrophomonas phage vB_SmaS-AXL_3 - Axeltriavirus
Acinetobacter phage IMEAB3 - Lokivirus
Rhodobacter phage RcHartney - Titanvirus
Pseudomonas phage 73 - Septimatrevirus
Burkholderia phage vB_BceS_KL1 - Kilunavirus
Alphaproteobacteria phage PhiJL001 - Mesyanzhinovviridae; Keylargovirus
Achromobacter phage JWX - Steinhofvirus
Pseudomonas phage Epa5 - Mesyanzhinovviridae; Bradleyvirinae; Epaquintavirus
Pseudomonas phage PaMx11 - Mesyanzhinovviridae; Bradleyvirinae; Abidjanvirus
Pseudomonas phage vB_PaeS_PAO1_Ab19 - Mesyanzhinovviridae; Bradleyvirinae; Abidjanvirus
Pseudomonas phage ZC01 - Mesyanzhinovviridae; Bradleyvirinae; Abidjanvirus
Pseudomonas phage vB_PaeS_PAO1_Ab18 - Mesyanzhinovviridae; Bradleyvirinae; Abidjanvirus
Bordetella phage FP1 - Mesyanzhinovviridae; Rabinowitzvirinae; Vojvodinavirus
Bordetella phage CN1 - Mesyanzhinovviridae; Rabinowitzvirinae; Vojvodinavirus
Bordetella phage MW2 - Mesyanzhinovviridae; Rabinowitzvirinae; Vojvodinavirus
Bordetella phage CN2 - Mesyanzhinovviridae; Rabinowitzvirinae; Vojvodinavirus
Pseudomonas phage Yua - Mesyanzhinovviridae; Rabinowitzvirinae; Yuavirus
Pseudomonas phage PAE1 - Mesyanzhinovviridae; Rabinowitzvirinae; Yuavirus
Pseudomonas phage MP1412 - Mesyanzhinovviridae; Rabinowitzvirinae; Yuavirus
Pseudomonas phage M6 - Mesyanzhinovviridae; Rabinowitzvirinae; Yuavirus
Pseudomonas phage Lydia - Mesyanzhinovviridae; Rabinowitzvirinae; Yuavirus
Pseudomonas phage LKO4 - Mesyanzhinovviridae; Rabinowitzvirinae; Yuavirus
Xanthomonas phage Mallos - Mesyanzhinovviridae; Bradleyvirinae; _Mallosvirus
Pseudomonas phage PaMx28 - Mesyanzhinovviridae; Bradleyvirinae; Pamexvirus
Pseudomonas phage phiH1 - Mesyanzhinovviridae; Bradleyvirinae; Pamexvirus
Pseudomonas phage phiH2 - Mesyanzhinovviridae; Bradleyvirinae; Pamexvirus
Pseudomonas phage AAT-1 - Mesyanzhinovviridae; Bradleyvirinae; Pamexvirus
Pseudomonas phage PaMx74 - Mesyanzhinovviridae; Bradleyvirinae; Pamexvirus
Janthinobacterium phage vB_JliM-Donnerlittchen - Mesyanzhinovviridae; Bradleyvirinae
Xanthomonas phage vB_Xar_IVIA-DoCa5 - Mesyanzhinovviridae; Bradleyvirinae; Docaquintavirus
Xanthomonas phage Elanor - Mesyanzhinovviridae; Bradleyvirinae; Elanorvirus
Xanthomonas phage Xoo-sp2 - Mesyanzhinovviridae; Bradleyvirinae; Xooduovirus
Stenotrophomonas phage vB_Sm_QDWS359 - Mesyanzhinovviridae; Bradleyvirinae; Bosavirus
Stenotrophomonas phage A1432 - Mesyanzhinovviridae; Bradleyvirinae; Ghuizhouvirus
Xanthomonas phage Xp12 - Mesyanzhinovviridae; Bradleyvirinae; Bosavirus
Xanthomonas phage FMYAK-P1 - Mesyanzhinovviridae; Bradleyvirinae; Bosavirus
Xanthomonas phage vB_Xar_IVIA-DoCa10 - Mesyanzhinovviridae; Bradleyvirinae; Bosavirus
Xanthomonas phage Bosa - Mesyanzhinovviridae; Bradleyvirinae; Bosavirus
Xanthomonas phage vB_Xar_IVIA-DoCa6 - Mesyanzhinovviridae; Bradleyvirinae; Bosavirus
Stenotrophomonas phage vB_SmaS-AXL_1 - Mesyanzhinovviridae; Bradleyvirinae; Bosavirus
Tree scale: 0.5

(c)

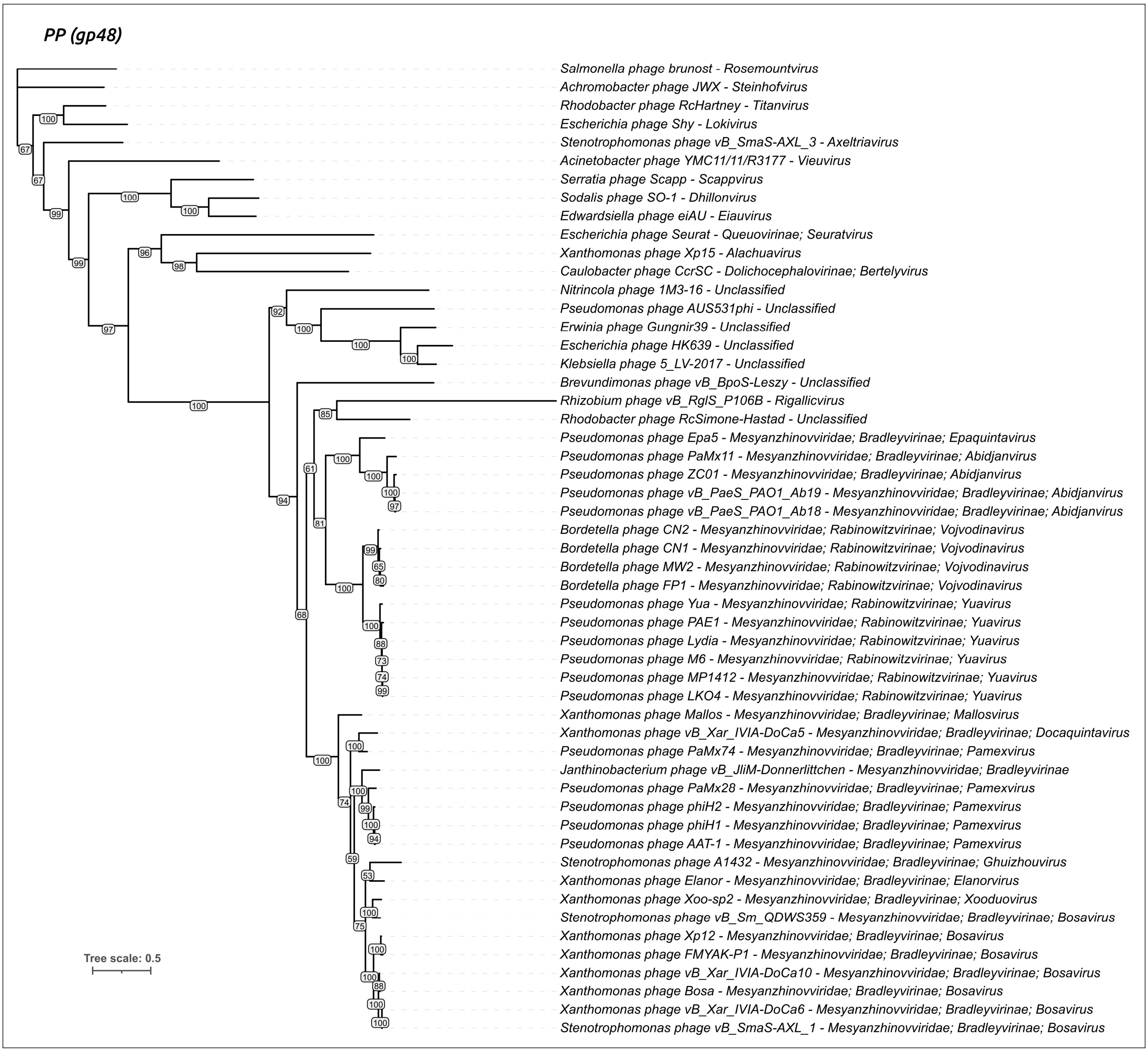
PP (gp48)
Salmonella phage brunost - Rosemountvirus
Achromobacter phage JWX - Steinhofvirus
Rhodobacter phage RcHartney - Titanvirus
Escherichia phage Shy - Lokivirus
Stenotrophomonas phage vB_SmaS-AXL_3 - Axeltriavirus
Acinetobacter phage YMC11/11/R3177 - Vieuvirus
Serratia phage Scapp - Scappvirus
Sodalis phage SO-1 - Dhillonvirus
Edwardsiella phage eiAU - Eiauvirus
Escherichia phage Seurat - Queuovirinae; Seuratvirus
Xanthomonas phage Xp15 - Alachuavirus
Caulobacter phage CcrSC - Dolichocephalovirinae; Bertelyvirus
Nitrincola phage 1M3-16 - Unclassified
Pseudomonas phage AUS531phi - Unclassified
Erwinia phage Gungnir39 - Unclassified
Escherichia phage HK639 - Unclassified
Klebsiella phage 5_LV-2017 - Unclassified
Brevundimonas phage vB_BpoS-Leszy - Unclassified
Rhizobium phage vB_RglS_P106B - Rigallicvirus
Rhodobacter phage RcSimone-Hastad - Unclassified
Pseudomonas phage Epa5 - Mesyanzhinovviridae; Bradleyvirinae; Epaquintavirus
Pseudomonas phage PaMx11 - Mesyanzhinovviridae; Bradleyvirinae; Abidjanvirus
Pseudomonas phage ZC01 - Mesyanzhinovviridae; Bradleyvirinae; Abidjanvirus
Pseudomonas phage vB_PaeS_PAO1_Ab19 - Mesyanzhinovviridae; Bradleyvirinae; Abidjanvirus
Pseudomonas phage vB_PaeS_PAO1_Ab18 - Mesyanzhinovviridae; Bradleyvirinae; Abidjanvirus
Bordetella phage CN2 - Mesyanzhinovviridae; Rabinowitzvirinae; Vojvodinavirus
Bordetella phage CN1 - Mesyanzhinovviridae; Rabinowitzvirinae; Vojvodinavirus
Bordetella phage MW2 - Mesyanzhinovviridae; Rabinowitzvirinae; Vojvodinavirus
Bordetella phage FP1 - Mesyanzhinovviridae; Rabinowitzvirinae; Vojvodinavirus
Pseudomonas phage Yua - Mesyanzhinovviridae; Rabinowitzvirinae; Yuavirus
Pseudomonas phage PAE1 - Mesyanzhinovviridae; Rabinowitzvirinae; Yuavirus
Pseudomonas phage Lydia - Mesyanzhinovviridae; Rabinowitzvirinae; Yuavirus
Pseudomonas phage M6 - Mesyanzhinovviridae; Rabinowitzvirinae; Yuavirus
Pseudomonas phage MP1412 - Mesyanzhinovviridae; Rabinowitzvirinae; Yuavirus
Pseudomonas phage LKO4 - Mesyanzhinovviridae; Rabinowitzvirinae; Yuavirus
Xanthomonas phage Mallos - Mesyanzhinovviridae; Bradleyvirinae; Mallosvirus
Xanthomonas phage vB_Xar_IVIA-DoCa5 - Mesyanzhinovviridae; Bradleyvirinae; Docaquintavirus
Pseudomonas phage PaMx74 - Mesyanzhinovviridae; Bradleyvirinae; Pamexvirus
Janthinobacterium phage vB_JliM-Donnerlittchen - Mesyanzhinovviridae; Bradleyvirinae
Pseudomonas phage PaMx28 - Mesyanzhinovviridae; Bradleyvirinae; Pamexvirus
Pseudomonas phage phiH2 - Mesyanzhinovviridae; Bradleyvirinae; Pamexvirus
Pseudomonas phage phiH1 - Mesyanzhinovviridae; Bradleyvirinae; Pamexvirus
Pseudomonas phage AAT-1 - Mesyanzhinovviridae; Bradleyvirinae; Pamexvirus
Stenotrophomonas phage A1432 - Mesyanzhinovviridae; Bradleyvirinae; Ghuizhouvirus
Xanthomonas phage Elanor - Mesyanzhinovviridae; Bradleyvirinae; Elanorvirus
Xanthomonas phage Xoo-sp2 - Mesyanzhinovviridae; Bradleyvirinae; Xooduovirus
Stenotrophomonas phage vB_Sm_QDWS359 - Mesyanzhinovviridae; Bradleyvirinae; Bosavirus
Xanthomonas phage Xp12 - Mesyanzhinovviridae; Bradleyvirinae; Bosavirus
Xanthomonas phage FMYAK-P1 - Mesyanzhinovviridae; Bradleyvirinae; Bosavirus
Xanthomonas phage vB_Xar_IVIA-DoCa10 - Mesyanzhinovviridae; Bradleyvirinae; Bosavirus
Xanthomonas phage Bosa - Mesyanzhinovviridae; Bradleyvirinae; Bosavirus
Xanthomonas phage vB_Xar_IVIA-DoCa6 - Mesyanzhinovviridae; Bradleyvirinae; Bosavirus
Stenotrophomonas phage vB_SmaS-AXL_1 - Mesyanzhinovviridae; Bradleyvirinae; Bosavirus
Tree scale: 0.5

(d)

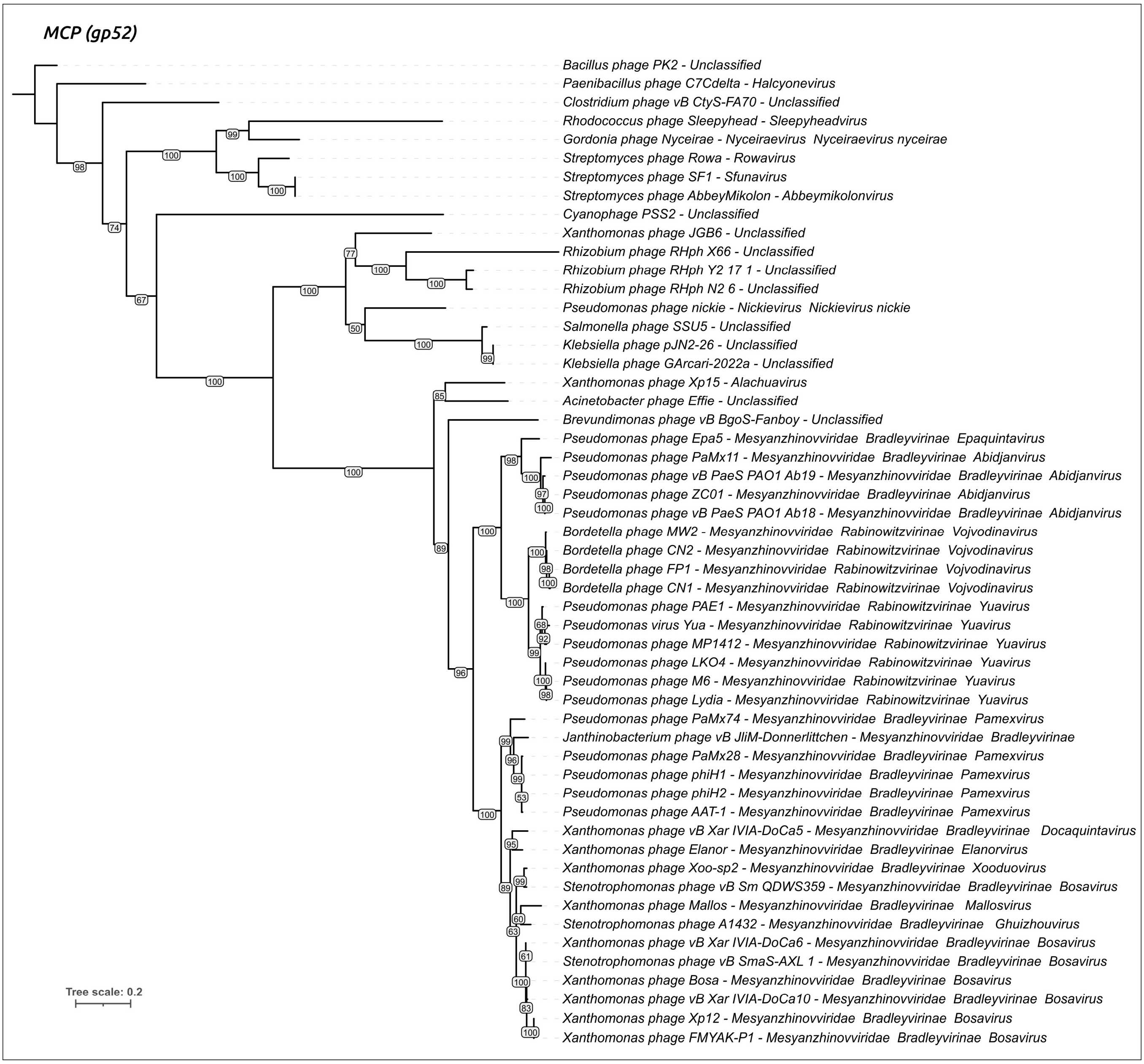

MCP (gp52)
Bacillus phage PK2 - Unclassified
Paenibacillus phage C7Cdelta - Halcyonevirus
Clostridium phage vB CtyS-FA70 - Unclassified
Rhodococcus phage Sleepyhead - Sleepyheadvirus
Gordonia phage Nyceirae - Nyceiraevirus Nyceiraevirus nyceirae
Streptomyces phage Rowa - Rowavirus
Streptomyces phage SF1 - Sfunavirus
Streptomyces phage AbbeyMikolon - Abbeymikolonvirus
Cyanophage PSS2 - Unclassified
Xanthomonas phage JGB6 - Unclassified
Rhizobium phage RHph X66 - Unclassified
Rhizobium phage RHph Y2 17 1 - Unclassified
Rhizobium phage RHph N2 6 - Unclassified
Pseudomonas phage nickie - Nickievirus Nickievirus nickie
Salmonella phage SSU5 - Unclassified
Klebsiella phage pJN2-26 - Unclassified
Klebsiella phage GArcari-2022a - Unclassified
Xanthomonas phage Xp15 - Alachuavirus
Acinetobacter phage Effie - Unclassified
Brevundimonas phage vB BgoS-Fanboy - Unclassified
Pseudomonas phage Epa5 - Mesyanzhinovviridae Bradleyvirinae Epaquintavirus
Pseudomonas phage PaMx11 - Mesyanzhinovviridae Bradleyvirinae Abidjanvirus
Pseudomonas phage vB PaeS PAO1 Ab19 - Mesyanzhinovviridae Bradleyvirinae Abidjanvirus
Pseudomonas phage ZC01 - Mesyanzhinovviridae Bradleyvirinae Abidjanvirus
Pseudomonas phage vB PaeS PAO1 Ab18 - Mesyanzhinovviridae Bradleyvirinae Abidjanvirus
Bordetella phage MW2 - Mesyanzhinovviridae Rabinowitzvirinae Vojvodinavirus
Bordetella phage CN2 - Mesyanzhinovviridae Rabinowitzvirinae Vojvodinavirus
Bordetella phage FP1 - Mesyanzhinovviridae Rabinowitzvirinae Vojvodinavirus
Bordetella phage CN1 - Mesyanzhinovviridae Rabinowitzvirinae Vojvodinavirus
Pseudomonas phage PAE1 - Mesyanzhinovviridae Rabinowitzvirinae Yuavirus
Pseudomonas virus Yua - Mesyanzhinovviridae Rabinowitzvirinae Yuavirus
Pseudomonas phage MP1412 - Mesyanzhinovviridae Rabinowitzvirinae Yuavirus
Pseudomonas phage LKO4 - Mesyanzhinovviridae Rabinowitzvirinae Yuavirus
Pseudomonas phage M6 - Mesyanzhinovviridae Rabinowitzvirinae Yuavirus
Pseudomonas phage Lydia - Mesyanzhinovviridae Rabinowitzvirinae Yuavirus
Pseudomonas phage PaMx74 - Mesyanzhinovviridae Bradleyvirinae Pamexvirus
Janthinobacterium phage vB JliM-Donnerlittchen - Mesyanzhinovviridae Bradleyvirinae
Pseudomonas phage PaMx28 - Mesyanzhinovviridae Bradleyvirinae Pamexvirus
Pseudomonas phage phiH1 - Mesyanzhinovviridae Bradleyvirinae Pamexvirus
Pseudomonas phage phiH2 - Mesyanzhinovviridae Bradleyvirinae Pamexvirus
Pseudomonas phage AAT-1 - Mesyanzhinovviridae Bradleyvirinae Pamexvirus
Xanthomonas phage vB Xar IVIA-DoCa5 - Mesyanzhinovviridae Bradleyvirinae Docaquintavirus
Xanthomonas phage Elanor - Mesyanzhinovviridae Bradleyvirinae Elanorvirus
Xanthomonas phage Xoo-sp2 - Mesyanzhinovviridae Bradleyvirinae Xooduovirus
Stenotrophomonas phage vB Sm QDWS359 - Mesyanzhinovviridae Bradleyvirinae Bosavirus
Xanthomonas phage Mallos - Mesyanzhinovviridae Bradleyvirinae Mallosvirus
Stenotrophomonas phage A1432 - Mesyanzhinovviridae Bradleyvirinae Ghuizhouvirus
Xanthomonas phage vB Xar IVIA-DoCa6 - Mesyanzhinovviridae Bradleyvirinae Bosavirus
Stenotrophomonas phage vB SmaS-AXL 1 - Mesyanzhinovviridae Bradleyvirinae Bosavirus
Xanthomonas phage Bosa - Mesyanzhinovviridae Bradleyvirinae Bosavirus
Xanthomonas phage vB Xar IVIA-DoCa10 - Mesyanzhinovviridae Bradleyvirinae Bosavirus
Xanthomonas phage Xp12 - Mesyanzhinovviridae Bradleyvirinae Bosavirus
Xanthomonas phage FMYAK-P1 - Mesyanzhinovviridae Bradleyvirinae Bosavirus
Tree scale: 0.2

(e)

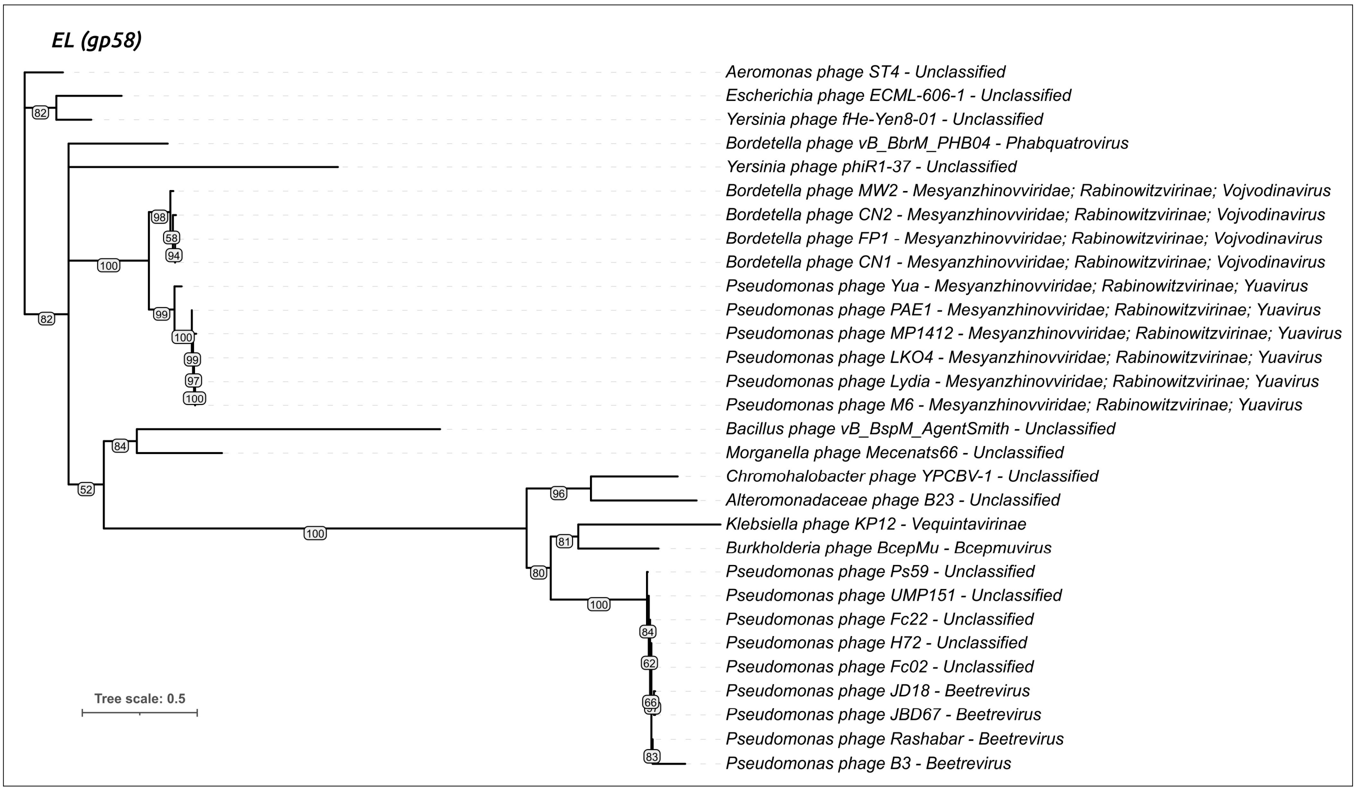

EL (gp58)
Aeromonas phage ST4 - Unclassified
Escherichia phage ECML-606-1 - Unclassified
Yersinia phage fHe-Yen8-01 - Unclassified
Bordetella phage vB_BbrM_PHB04 - Phabquatrovirus
Yersinia phage phiR1-37 - Unclassified
Bordetella phage MW2 - Mesyanzhinovviridae; Rabinowitzvirinae; Vojvodinavirus
Bordetella phage CN2 - Mesyanzhinovviridae; Rabinowitzvirinae; Vojvodinavirus
Bordetella phage FP1 - Mesyanzhinovviridae; Rabinowitzvirinae; Vojvodinavirus
Bordetella phage CN1 - Mesyanzhinovviridae; Rabinowitzvirinae; Vojvodinavirus
Pseudomonas phage Yua - Mesyanzhinovviridae; Rabinowitzvirinae; Yuavirus
Pseudomonas phage PAE1 - Mesyanzhinovviridae; Rabinowitzvirinae; Yuavirus
Pseudomonas phage MP1412 - Mesyanzhinovviridae; Rabinowitzvirinae; Yuavirus
Pseudomonas phage LKO4 - Mesyanzhinovviridae; Rabinowitzvirinae; Yuavirus
Pseudomonas phage Lydia - Mesyanzhinovviridae; Rabinowitzvirinae; Yuavirus
Pseudomonas phage M6 - Mesyanzhinovviridae; Rabinowitzvirinae; Yuavirus
Bacillus phage vB_BspM_AgentSmith - Unclassified
Morganella phage Mecenats66 - Unclassified
Chromohalobacter phage YPCBV-1 - Unclassified
Alteromonadaceae phage B23 - Unclassified
Klebsiella phage KP12 - Vequintavirinae
Burkholderia phage BcepMu - Bcepmuvirus
Pseudomonas phage Ps59 - Unclassified
Pseudomonas phage UMP151 - Unclassified
Pseudomonas phage Fc22 - Unclassified
Pseudomonas phage H72 - Unclassified
Pseudomonas phage Fc02 - Unclassified
Pseudomonas phage JD18 - Beetrevirus
Pseudomonas phage JBD67 - Beetrevirus
Pseudomonas phage Rashabar - Beetrevirus
Pseudomonas phage B3 - Beetrevirus
Tree scale: 0.5

(f)

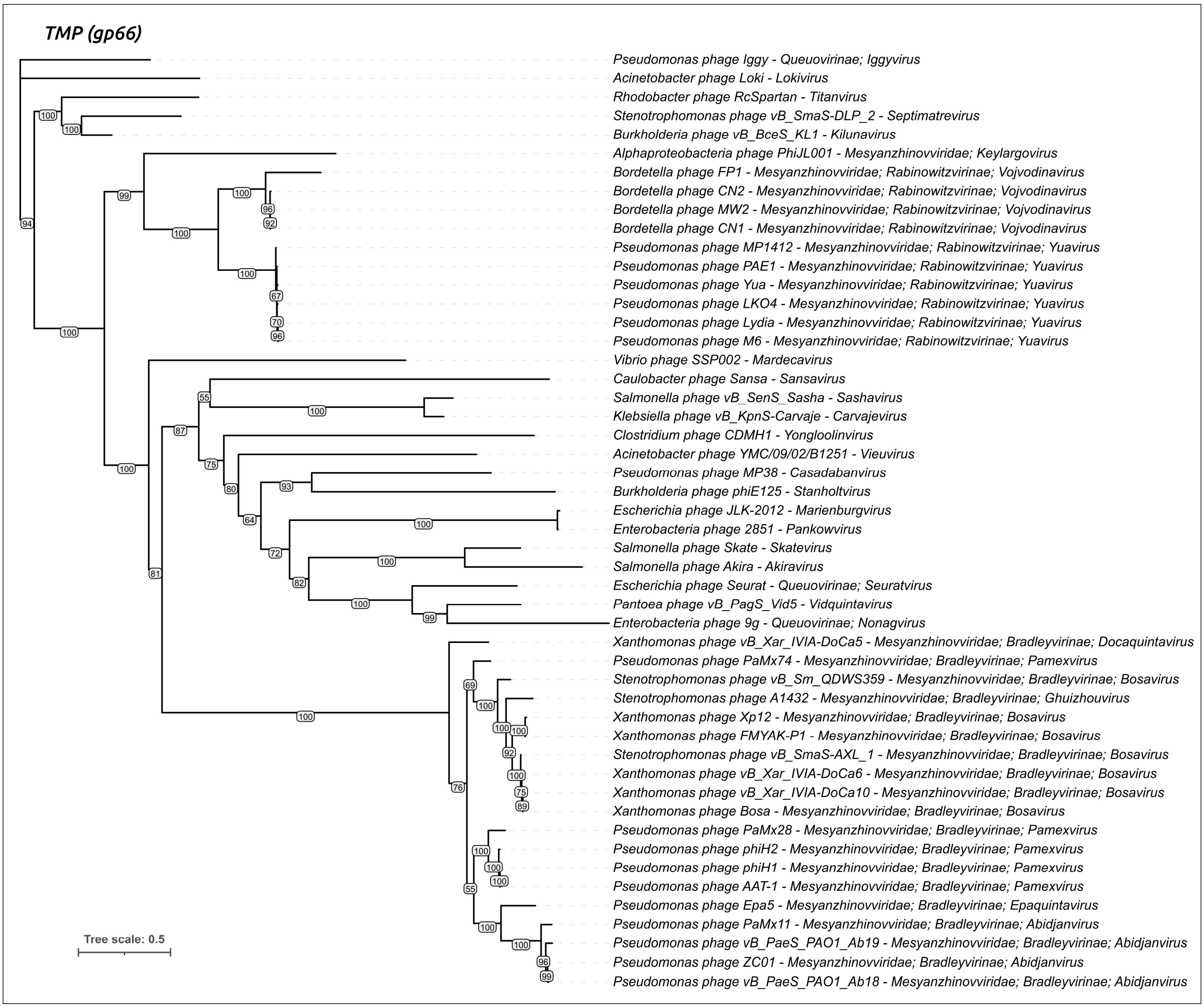

TMP (gp66)
Pseudomonas phage Iggy - Queuovirinae; Iggyvirus
Acinetobacter phage Loki - Lokivirus
Rhodobacter phage RcSpartan - Titanvirus
Stenotrophomonas phage vB_SmaS-DLP_2 - Septimatrevirus
Burkholderia phage vB_BceS_KL1 - Kilunavirus
Alphaproteobacteria phage PhiJL001 - Mesyanzhinovviridae; Keylargovirus
Bordetella phage FP1 - Mesyanzhinovviridae; Rabinowitzvirinae; Vojvodinavirus
Bordetella phage CN2 - Mesyanzhinovviridae; Rabinowitzvirinae; Vojvodinavirus
Bordetella phage MW2 - Mesyanzhinovviridae; Rabinowitzvirinae; Vojvodinavirus
Bordetella phage CN1 - Mesyanzhinovviridae; Rabinowitzvirinae; Vojvodinavirus
Pseudomonas phage MP1412 - Mesyanzhinovviridae; Rabinowitzvirinae; Yuavirus
Pseudomonas phage PAE1 - Mesyanzhinovviridae; Rabinowitzvirinae; Yuavirus
Pseudomonas phage Yua - Mesyanzhinovviridae; Rabinowitzvirinae; Yuavirus
Pseudomonas phage LKO4 - Mesyanzhinovviridae; Rabinowitzvirinae; Yuavirus
Pseudomonas phage Lydia - Mesyanzhinovviridae; Rabinowitzvirinae; Yuavirus
Pseudomonas phage M6 - Mesyanzhinovviridae; Rabinowitzvirinae; Yuavirus
Vibrio phage SSP002 - Mardecavirus
Caulobacter phage Sansa - Sansavirus
Salmonella phage vB_SenS_Sasha - Sashavirus
Klebsiella phage vB_KpnS-Carvaje - Carvajevirus
Clostridium phage CDMH1 - Yongloolinvirus
Acinetobacter phage YMC/09/02/B1251 - Vieuvirus
Pseudomonas phage MP38 - Casadabanvirus
Burkholderia phage phiE125 - Stanholtvirus
Escherichia phage JLK-2012 - Marienburgvirus
Enterobacteria phage 2851 - Pankowvirus
Salmonella phage Skate - Skatevirus
Salmonella phage Akira - Akiravirus
Escherichia phage Seurat - Queuovirinae; Seuratvirus
Pantoea phage vB_PagS_Vid5 - Vidquintavirus
Enterobacteria phage 9g - Queuovirinae; Nonagvirus
Xanthomonas phage vB_Xar_IVIA-DoCa5 - Mesyanzhinovviridae; Bradleyvirinae; Docaquintavirus
Pseudomonas phage PaMx74 - Mesyanzhinovviridae; Bradleyvirinae; Pamexvirus
Stenotrophomonas phage vB_Sm_QDWS359 - Mesyanzhinovviridae; Bradleyvirinae; Bosavirus
Stenotrophomonas phage A1432 - Mesyanzhinovviridae; Bradleyvirinae; Ghuizhouvirus
Xanthomonas phage Xp12 - Mesyanzhinovviridae; Bradleyvirinae; Bosavirus
Xanthomonas phage FMYAK-P1 - Mesyanzhinovviridae; Bradleyvirinae; Bosavirus
Stenotrophomonas phage vB_SmaS-AXL_1 - Mesyanzhinovviridae; Bradleyvirinae; Bosavirus
Xanthomonas phage vB_Xar_IVIA-DoCa6 - Mesyanzhinovviridae; Bradleyvirinae; Bosavirus
Xanthomonas phage vB_Xar_IVIA-DoCa10 - Mesyanzhinovviridae; Bradleyvirinae; Bosavirus
Xanthomonas phage Bosa - Mesyanzhinovviridae; Bradleyvirinae; Bosavirus
Pseudomonas phage PaMx28 - Mesyanzhinovviridae; Bradleyvirinae; Pamexvirus
Pseudomonas phage phiH2 - Mesyanzhinovviridae; Bradleyvirinae; Pamexvirus
Pseudomonas phage phiH1 - Mesyanzhinovviridae; Bradleyvirinae; Pamexvirus
Pseudomonas phage AAT-1 - Mesyanzhinovviridae; Bradleyvirinae; Pamexvirus
Pseudomonas phage Epa5 - Mesyanzhinovviridae; Bradleyvirinae; Epaquintavirus
Pseudomonas phage PaMx11 - Mesyanzhinovviridae; Bradleyvirinae; Abidjanvirus
Pseudomonas phage vB_PaeS_PAO1_Ab19 - Mesyanzhinovviridae; Bradleyvirinae; Abidjanvirus
Pseudomonas phage ZC01 - Mesyanzhinovviridae; Bradleyvirinae; Abidjanvirus
Pseudomonas phage vB_PaeS_PAO1_Ab18 - Mesyanzhinovviridae; Bradleyvirinae; Abidjanvirus
Tree scale: 0.5

(g)

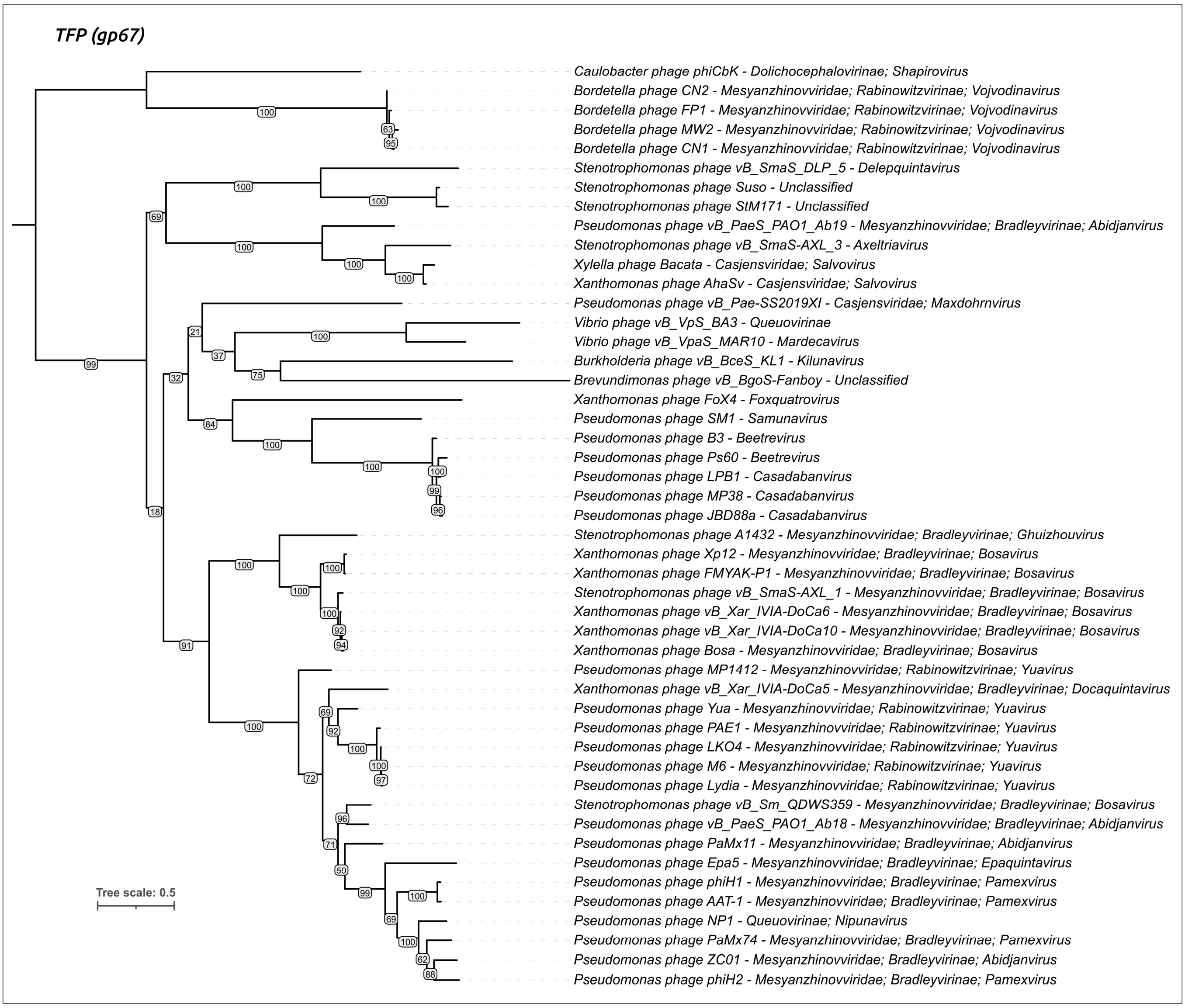

TFP (gp67)
Caulobacter phage phiCbK - Dolichocephalovirinae; Shapirovirus
Bordetella phage CN2 - Mesyanzhinovviridae; Rabinowitzvirinae; Vojvodinavirus
Bordetella phage FP1 - Mesyanzhinovviridae; Rabinowitzvirinae; Vojvodinavirus
Bordetella phage MW2 - Mesyanzhinovviridae; Rabinowitzvirinae; Vojvodinavirus
Bordetella phage CN1 - Mesyanzhinovviridae; Rabinowitzvirinae; Vojvodinavirus
Stenotrophomonas phage vB_SmaS_DLP_5 - Delepquintavirus
Stenotrophomonas phage Suso - Unclassified
Stenotrophomonas phage StM171 - Unclassified
Pseudomonas phage vB_PaeS_PAO1_Ab19 - Mesyanzhinovviridae; Bradleyvirinae; Abidjanvirus
Stenotrophomonas phage vB_SmaS-AXL_3 - Axeltriavirus
Xylella phage Bacata - Casjensviridae; Salvovirus
Xanthomonas phage AhaSv - Casjensviridae; Salvovirus
Pseudomonas phage vB_Pae-SS2019XI - Casjensviridae; Maxdohrnvirus
Vibrio phage vB_VpS_BA3 - Queuovirinae
Vibrio phage vB_VpaS_MAR10 - Mardecavirus
Burkholderia phage vB_BceS_KL1 - Kilunavirus
Brevundimonas phage vB_BgoS-Fanboy - Unclassified
Xanthomonas phage FoX4 - Foxquatrovirus
Pseudomonas phage SM1 - Samunavirus
Pseudomonas phage B3 - Beetrevirus
Pseudomonas phage Ps60 - Beetrevirus
Pseudomonas phage LPB1 - Casadabanvirus
Pseudomonas phage MP38 - Casadabanvirus
Pseudomonas phage JBD88a - Casadabanvirus
Stenotrophomonas phage A1432 - Mesyanzhinovviridae; Bradleyvirinae; Ghuizhouvirus
Xanthomonas phage Xp12 - Mesyanzhinovviridae; Bradleyvirinae; Bosavirus
Xanthomonas phage FMYAK-P1 - Mesyanzhinovviridae; Bradleyvirinae; Bosavirus
Stenotrophomonas phage vB_SmaS-AXL_1 - Mesyanzhinovviridae; Bradleyvirinae; Bosavirus
Xanthomonas phage vB_Xar_IVIA-DoCa6 - Mesyanzhinovviridae; Bradleyvirinae; Bosavirus
Xanthomonas phage vB_Xar_IVIA-DoCa10 - Mesyanzhinovviridae; Bradleyvirinae; Bosavirus
Xanthomonas phage Bosa - Mesyanzhinovviridae; Bradleyvirinae; Bosavirus
Pseudomonas phage MP1412 - Mesyanzhinovviridae; Rabinowitzvirinae; Yuavirus
Xanthomonas phage vB_Xar_IVIA-DoCa5 - Mesyanzhinovviridae; Bradleyvirinae; Docaquintavirus
Pseudomonas phage Yua - Mesyanzhinovviridae; Rabinowitzvirinae; Yuavirus
Pseudomonas phage PAE1 - Mesyanzhinovviridae; Rabinowitzvirinae; Yuavirus
Pseudomonas phage LKO4 - Mesyanzhinovviridae; Rabinowitzvirinae; Yuavirus
Pseudomonas phage M6 - Mesyanzhinovviridae; Rabinowitzvirinae; Yuavirus
Pseudomonas phage Lydia - Mesyanzhinovviridae; Rabinowitzvirinae; Yuavirus
Stenotrophomonas phage vB_Sm_QDWS359 - Mesyanzhinovviridae; Bradleyvirinae; Bosavirus
Pseudomonas phage vB_PaeS_PAO1_Ab18 - Mesyanzhinovviridae; Bradleyvirinae; Abidjanvirus
Pseudomonas phage PaMx11 - Mesyanzhinovviridae; Bradleyvirinae; Abidjanvirus
Pseudomonas phage Epa5 - Mesyanzhinovviridae; Bradleyvirinae; Epaquintavirus
Pseudomonas phage phiH1 - Mesyanzhinovviridae; Bradleyvirinae; Pamexvirus
Pseudomonas phage AAT-1 - Mesyanzhinovviridae; Bradleyvirinae; Pamexvirus
Pseudomonas phage NP1 - Queuovirinae; Nipunavirus
Pseudomonas phage PaMx74 - Mesyanzhinovviridae; Bradleyvirinae; Pamexvirus
Pseudomonas phage ZC01 - Mesyanzhinovviridae; Bradleyvirinae; Abidjanvirus
Pseudomonas phage phiH2 - Mesyanzhinovviridae; Bradleyvirinae; Pamexvirus
Tree scale: 0.5

(h)

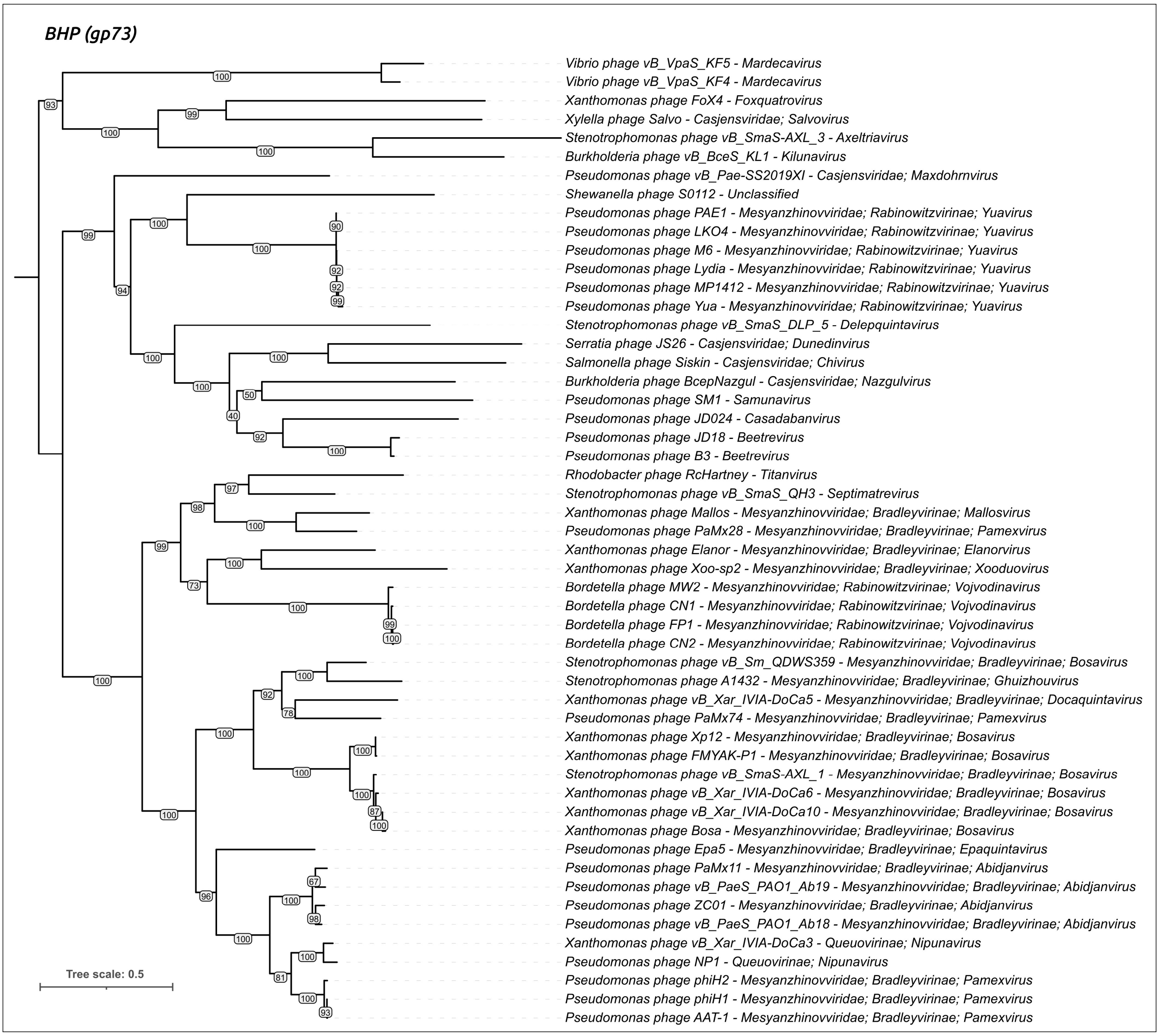
BHP (gp73)
Vibrio phage vB_VpaS_KF5 - Mardecavirus
Vibrio phage vB_VpaS_KF4 - Mardecavirus
Xanthomonas phage FoX4 - Foxquatrovirus
Xylella phage Salvo - Casjensviridae; Salvovirus
Stenotrophomonas phage vB_SmaS-AXL_3 - Axeltriavirus
Burkholderia phage vB_BceS_KL1 - Kilunavirus
Pseudomonas phage vB_Pae-SS2019XI - Casjensviridae; Maxdohrnvirus
Shewanella phage S0112 - Unclassified
Pseudomonas phage PAE1 - Mesyanzhinovviridae; Rabinowitzvirinae; Yuavirus
Pseudomonas phage LKO4 - Mesyanzhinovviridae; Rabinowitzvirinae; Yuavirus
Pseudomonas phage M6 - Mesyanzhinovviridae; Rabinowitzvirinae; Yuavirus
Pseudomonas phage Lydia - Mesyanzhinovviridae; Rabinowitzvirinae; Yuavirus
Pseudomonas phage MP1412 - Mesyanzhinovviridae; Rabinowitzvirinae; Yuavirus
Pseudomonas phage Yua - Mesyanzhinovviridae; Rabinowitzvirinae; Yuavirus
Stenotrophomonas phage vB_SmaS_DLP_5 - Delepquintavirus
Serratia phage JS26 - Casjensviridae; Dunedinvirus
Salmonella phage Siskin - Casjensviridae; Chivirus
Burkholderia phage BcepNazgul - Casjensviridae; Nazgulvirus
Pseudomonas phage SM1 - Samunavirus
Pseudomonas phage JD024 - Casadabanvirus
Pseudomonas phage JD18 - Beetrevirus
Pseudomonas phage B3 - Beetrevirus
Rhodobacter phage RcHartney - Titanvirus
Stenotrophomonas phage vB_SmaS_QH3 - Septimatrevirus
Xanthomonas phage Mallos - Mesyanzhinovviridae; Bradleyvirinae; Mallosvirus
Pseudomonas phage PaMx28 - Mesyanzhinovviridae; Bradleyvirinae; Pamexvirus
Xanthomonas phage Elanor - Mesyanzhinovviridae; Bradleyvirinae; Elanorvirus
Xanthomonas phage Xoo-sp2 - Mesyanzhinovviridae; Bradleyvirinae; Xooduovirus
Bordetella phage MW2 - Mesyanzhinovviridae; Rabinowitzvirinae; Vojvodinavirus
Bordetella phage CN1 - Mesyanzhinovviridae; Rabinowitzvirinae; Vojvodinavirus
Bordetella phage FP1 - Mesyanzhinovviridae; Rabinowitzvirinae; Vojvodinavirus
Bordetella phage CN2 - Mesyanzhinovviridae; Rabinowitzvirinae; Vojvodinavirus
Stenotrophomonas phage vB_Sm_QDWS359 - Mesyanzhinovviridae; Bradleyvirinae; Bosavirus
Stenotrophomonas phage A1432 - Mesyanzhinovviridae; Bradleyvirinae; Ghuizhouvirus
Xanthomonas phage vB_Xar_IVIA-DoCa5 - Mesyanzhinovviridae; Bradleyvirinae; Docaquintavirus
Pseudomonas phage PaMx74 - Mesyanzhinovviridae; Bradleyvirinae; Pamexvirus
Xanthomonas phage Xp12 - Mesyanzhinovviridae; Bradleyvirinae; Bosavirus
Xanthomonas phage FMYAK-P1 - Mesyanzhinovviridae; Bradleyvirinae; Bosavirus
Stenotrophomonas phage vB_SmaS-AXL_1 - Mesyanzhinovviridae; Bradleyvirinae; Bosavirus
Xanthomonas phage vB_Xar_IVIA-DoCa6 - Mesyanzhinovviridae; Bradleyvirinae; Bosavirus
Xanthomonas phage vB_Xar_IVIA-DoCa10 - Mesyanzhinovviridae; Bradleyvirinae; Bosavirus
Xanthomonas phage Bosa - Mesyanzhinovviridae; Bradleyvirinae; Bosavirus
Pseudomonas phage Epa5 - Mesyanzhinovviridae; Bradleyvirinae; Epaquintavirus
Pseudomonas phage PaMx11 - Mesyanzhinovviridae; Bradleyvirinae; Abidjanvirus
Pseudomonas phage vB_PaeS_PAO1_Ab19 - Mesyanzhinovviridae; Bradleyvirinae; Abidjanvirus
Pseudomonas phage ZC01 - Mesyanzhinovviridae; Bradleyvirinae; Abidjanvirus
Pseudomonas phage vB_PaeS_PAO1_Ab18 - Mesyanzhinovviridae; Bradleyvirinae; Abidjanvirus
Xanthomonas phage vB_Xar_IVIA-DoCa3 - Queuovirinae; Nipunavirus
Pseudomonas phage NP1 - Queuovirinae; Nipunavirus
Pseudomonas phage phiH2 - Mesyanzhinovviridae; Bradleyvirinae; Pamexvirus
Pseudomonas phage phiH1 - Mesyanzhinovviridae; Bradleyvirinae; Pamexvirus
Pseudomonas phage AAT-1 - Mesyanzhinovviridae; Bradleyvirinae; Pamexvirus
Tree scale: 0.5

(i)

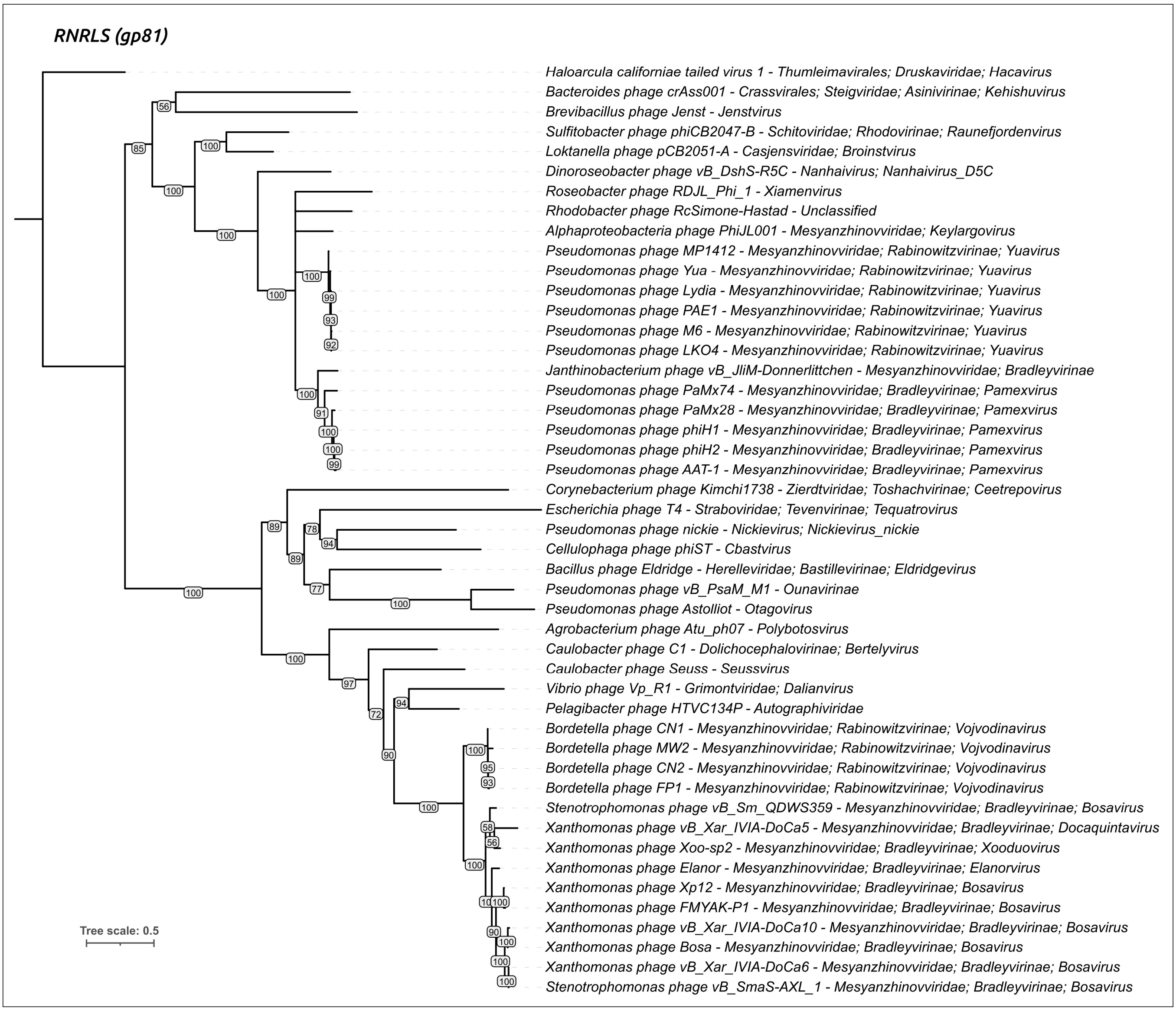

(j)

Supplementary Figure S5. Maximum likelihood phylogenetic trees based on amino acid sequences of phage proteins. The scale bar shows the number of estimated substitutions per site. Taxonomy is indicated in labels and legends. Branches with a bootstrap support lower than 50% have been deleted. Bootstrap values are shown near their branches. (**a**) DUHMT (gp4), the tree was rooted to *Bacillus* phage G. (**b**) DNAP (gp8), the tree was unrooted. (**c**) TLS (gp47), the tree was unrooted. (**d**) PP (gp48), the tree was unrooted. (**e**) MCP (gp52), the tree was rooted to *Bacillus* phage PK2. (**f**) EL (gp58), the tree was unrooted. (**g**) TMP (gp66), the tree was unrooted. (**h**) TFP (gp67), the tree was rooted to the midpoint. (**i**) BHP (gp73), the tree was rooted to the midpoint. (**j**) RNRLS (gp81), the tree was rooted to *Haloarcula californiae* tailed virus 1.

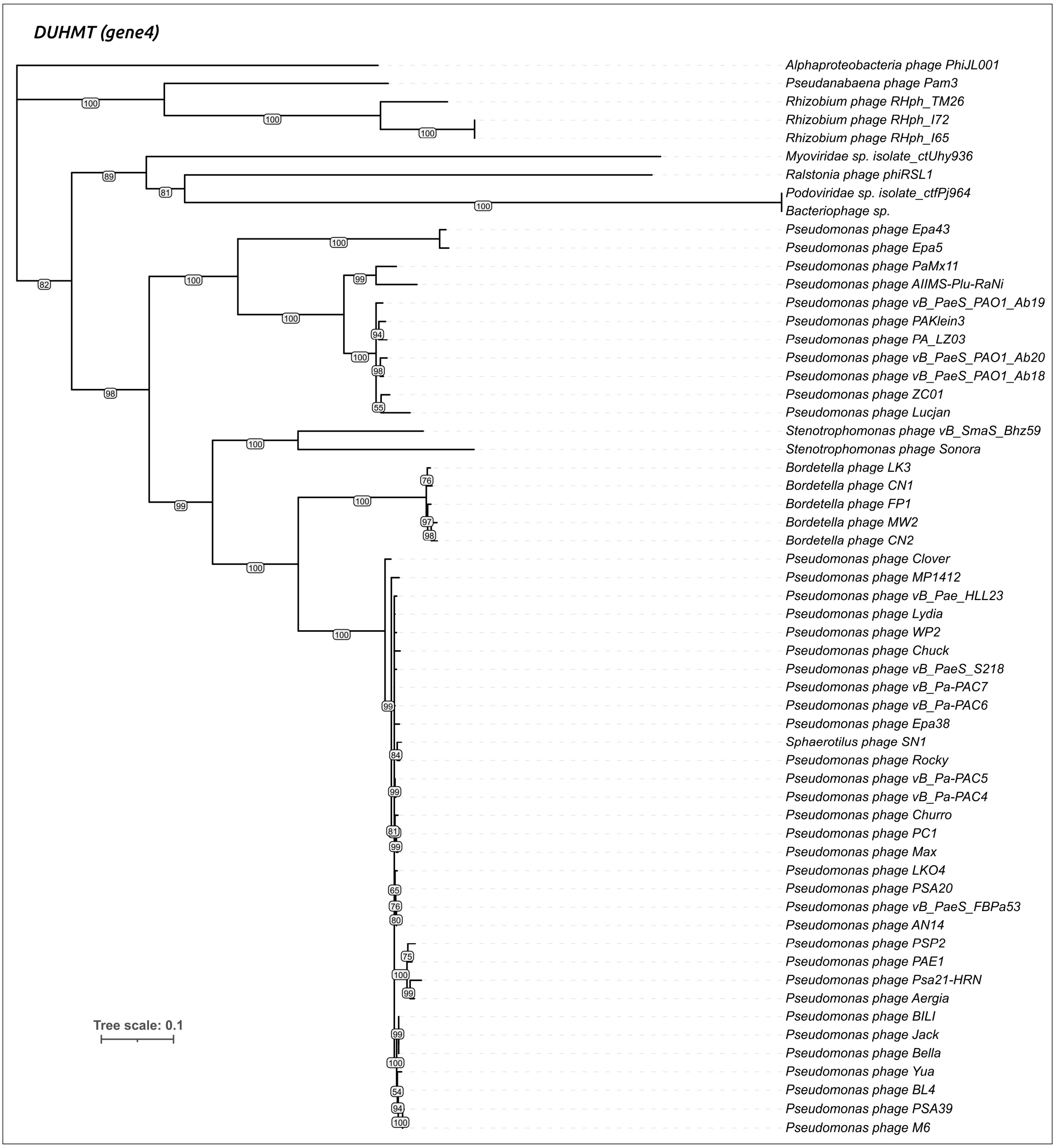
DUHMT (gene4)
Alphaproteobacteria phage PhiJL001
Pseudanabaena phage Pam3
Rhizobium phage RHph_TM26
Rhizobium phage RHph_I72
Rhizobium phage RHph_I65
Myoviridae sp. isolate_ctUhy936
Ralstonia phage phiRSL1
Podoviridae sp. isolate_ctfPj964
Bacteriophage sp.
Pseudomonas phage Epa43
Pseudomonas phage Epa5
Pseudomonas phage PaMx11
Pseudomonas phage AIIMS-Plu-RaNi
Pseudomonas phage vB_PaeS_PAO1_Ab19
Pseudomonas phage PAKlein3
Pseudomonas phage PA_LZ03
Pseudomonas phage vB_PaeS_PAO1_Ab20
Pseudomonas phage vB_PaeS_PAO1_Ab18
Pseudomonas phage ZC01
Pseudomonas phage Lucjan
Stenotrophomonas phage vB_SmaS_Bhz59
Stenotrophomonas phage Sonora
Bordetella phage LK3
Bordetella phage CN1
Bordetella phage FP1
Bordetella phage MW2
Bordetella phage CN2
Pseudomonas phage Clover
Pseudomonas phage MP1412
Pseudomonas phage vB_Pae_HLL23
Pseudomonas phage Lydia
Pseudomonas phage WP2
Pseudomonas phage Chuck
Pseudomonas phage vB_PaeS_S218
Pseudomonas phage vB_Pa-PAC7
Pseudomonas phage vB_Pa-PAC6
Pseudomonas phage Epa38
Sphaerotilus phage SN1
Pseudomonas phage Rocky
Pseudomonas phage vB_Pa-PAC5
Pseudomonas phage vB_Pa-PAC4
Pseudomonas phage Churro
Pseudomonas phage PC1
Pseudomonas phage Max
Pseudomonas phage LKO4
Pseudomonas phage PSA20
Pseudomonas phage vB_PaeS_FBPa53
Pseudomonas phage AN14
Pseudomonas phage PSP2
Pseudomonas phage PAE1
Pseudomonas phage Psa21-HRN
Pseudomonas phage Aergia
Pseudomonas phage BILI
Pseudomonas phage Jack
Pseudomonas phage Bella
Pseudomonas phage Yua
Pseudomonas phage BL4
Pseudomonas phage PSA39
Pseudomonas phage M6
Tree scale: 0.1

(a)

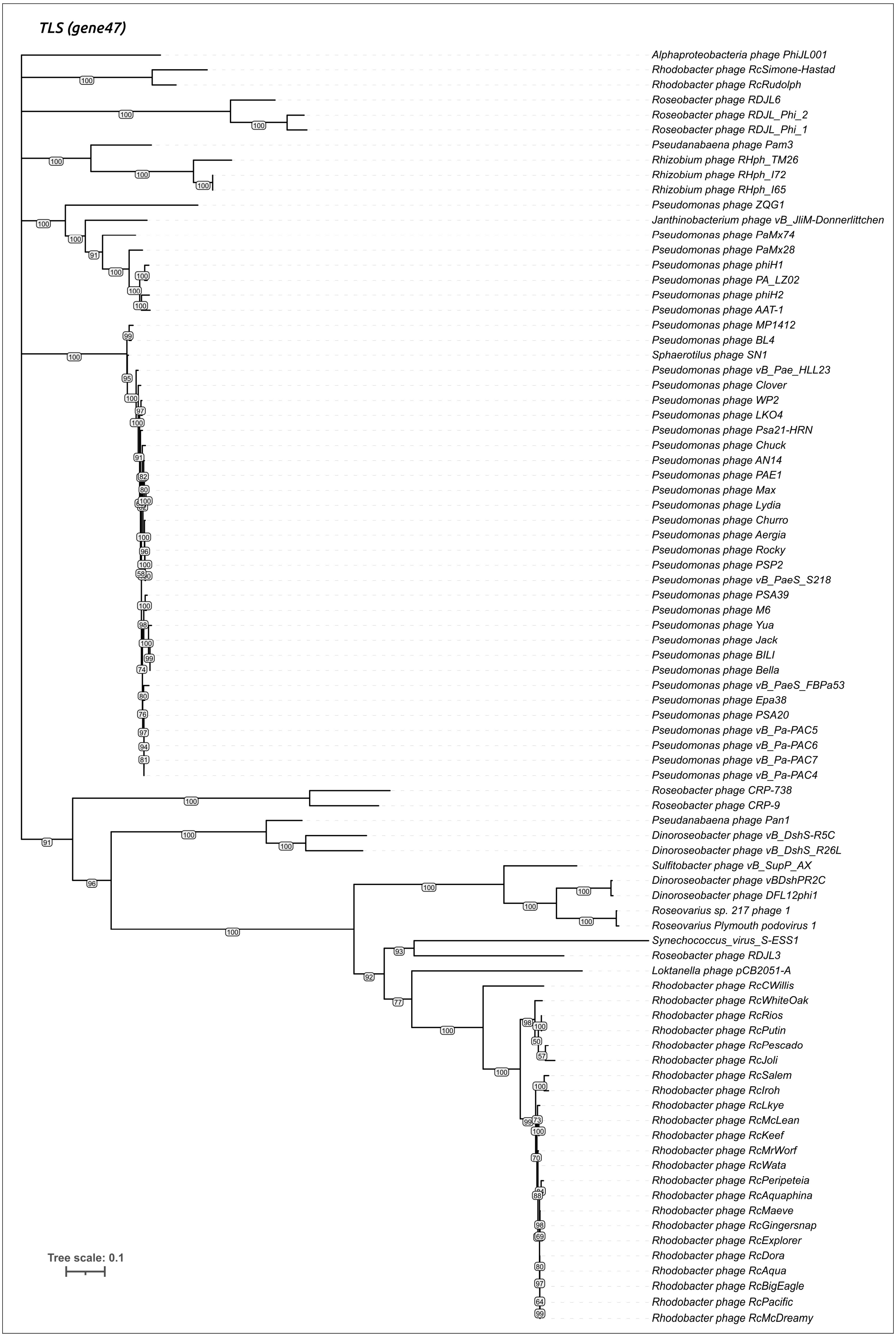
TLS (gene47)
Alphaproteobacteria phage PhiJL001
Rhodobacter phage RcSimone-Hastad
Rhodobacter phage RcRudolph
Roseobacter phage RDJL6
Roseobacter phage RDJL_Phi_2
Roseobacter phage RDJL_Phi_1
Pseudanabaena phage Pam3
Rhizobium phage RHph_TM26
Rhizobium phage RHph_I72
Rhizobium phage RHph_I65
Pseudomonas phage ZQG1
Janthinobacterium phage vB_JliM-Donnerlittchen
Pseudomonas phage PaMx74
Pseudomonas phage PaMx28
Pseudomonas phage phiH1
Pseudomonas phage PA_LZ02
Pseudomonas phage phiH2
Pseudomonas phage AAT-1
Pseudomonas phage MP1412
Pseudomonas phage BL4
Sphaerotilus phage SN1
Pseudomonas phage vB_Pae_HLL23
Pseudomonas phage Clover
Pseudomonas phage WP2
Pseudomonas phage LKO4
Pseudomonas phage Psa21-HRN
Pseudomonas phage Chuck
Pseudomonas phage AN14
Pseudomonas phage PAE1
Pseudomonas phage Max
Pseudomonas phage Lydia
Pseudomonas phage Churro
Pseudomonas phage Aergia
Pseudomonas phage Rocky
Pseudomonas phage PSP2
Pseudomonas phage vB_PaeS_S218
Pseudomonas phage PSA39
Pseudomonas phage M6
Pseudomonas phage Yua
Pseudomonas phage Jack
Pseudomonas phage BILI
Pseudomonas phage Bella
Pseudomonas phage vB_PaeS_FBPa53
Pseudomonas phage Epa38
Pseudomonas phage PSA20
Pseudomonas phage vB_Pa-PAC5
Pseudomonas phage vB_Pa-PAC6
Pseudomonas phage vB_Pa-PAC7
Pseudomonas phage vB_Pa-PAC4
Roseobacter phage CRP-738
Roseobacter phage CRP-9
Pseudanabaena phage Pan1
Dinoroseobacter phage vB_DshS-R5C
Dinoroseobacter phage vB_DshS_R26L
Sulfitobacter phage vB_SupP_AX
Dinoroseobacter phage vBDshPR2C
Dinoroseobacter phage DFL12phi1
Roseovarius sp. 217 phage 1
Roseovarius Plymouth podovirus 1
Synechococcus_virus_S-ESS1
Roseobacter phage RDJL3
Loktanella phage pCB2051-A
Rhodobacter phage RcCWillis
Rhodobacter phage RcWhiteOak
Rhodobacter phage RcRios
Rhodobacter phage RcPutin
Rhodobacter phage RcPescado
Rhodobacter phage RcJoli
Rhodobacter phage RcSalem
Rhodobacter phage RcIroh
Rhodobacter phage RcLkye
Rhodobacter phage RcMcLean
Rhodobacter phage RcKeef
Rhodobacter phage RcMrWorf
Rhodobacter phage RcWata
Rhodobacter phage RcPeripeteia
Rhodobacter phage RcAquaphina
Rhodobacter phage RcMaeve
Rhodobacter phage RcGingersnap
Rhodobacter phage RcExplorer
Rhodobacter phage RcDora
Rhodobacter phage RcAqua
Rhodobacter phage RcBigEagle
Rhodobacter phage RcPacific
Rhodobacter phage RcMcDreamy
Tree scale: 0.1

(b)

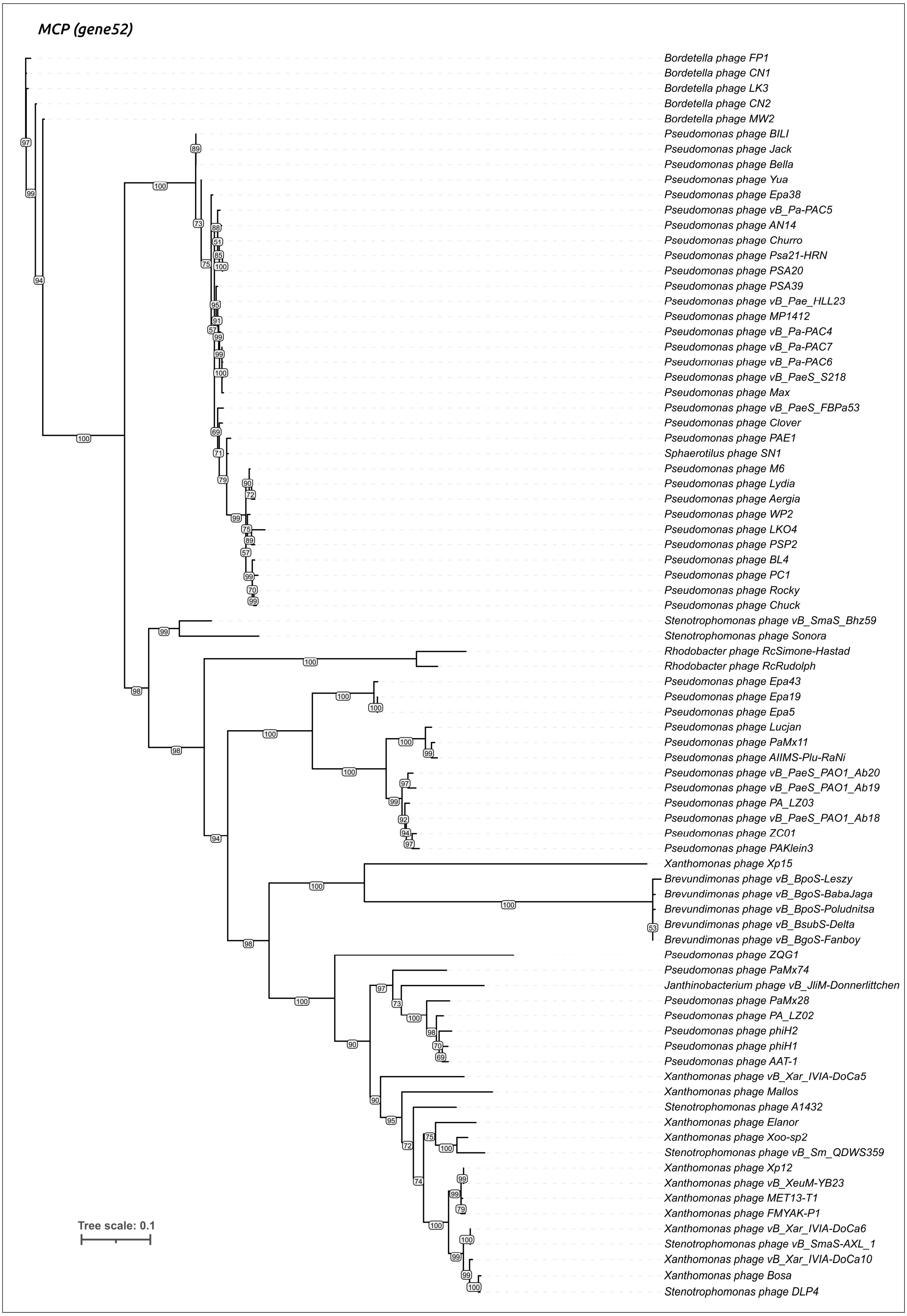

MCP (gene52)
Bordetella phage FP1
Bordetella phage CN1
Bordetella phage LK3
Bordetella phage CN2
Bordetella phage MW2
Pseudomonas phage BILI
Pseudomonas phage Jack
Pseudomonas phage Bella
Pseudomonas phage Yua
Pseudomonas phage Epa38
Pseudomonas phage vB_Pa-PAC5
Pseudomonas phage AN14
Pseudomonas phage Churro
Pseudomonas phage Psa21-HRN
Pseudomonas phage PSA20
Pseudomonas phage PSA39
Pseudomonas phage vB_Pae_HLL23
Pseudomonas phage MP1412
Pseudomonas phage vB_Pa-PAC4
Pseudomonas phage vB_Pa-PAC7
Pseudomonas phage vB_Pa-PAC6
Pseudomonas phage vB_PaeS_S218
Pseudomonas phage Max
Pseudomonas phage vB_PaeS_FBPa53
Pseudomonas phage Clover
Pseudomonas phage PAE1
Sphaerotilus phage SN1
Pseudomonas phage M6
Pseudomonas phage Lydia
Pseudomonas phage Aergia
Pseudomonas phage WP2
Pseudomonas phage LKO4
Pseudomonas phage PSP2
Pseudomonas phage BL4
Pseudomonas phage PC1
Pseudomonas phage Rocky
Pseudomonas phage Chuck
Stenotrophomonas phage vB_SmaS_Bhz59
Stenotrophomonas phage Sonora
Rhodobacter phage RcSimone-Hastad
Rhodobacter phage RcRudolph
Pseudomonas phage Epa43
Pseudomonas phage Epa19
Pseudomonas phage Epa5
Pseudomonas phage Lucjan
Pseudomonas phage PaMx11
Pseudomonas phage AIIMS-Plu-RaNi
Pseudomonas phage vB_PaeS_PAO1_Ab20
Pseudomonas phage vB_PaeS_PAO1_Ab19
Pseudomonas phage PA_LZ03
Pseudomonas phage vB_PaeS_PAO1_Ab18
Pseudomonas phage ZC01
Pseudomonas phage PAKlein3
Xanthomonas phage Xp15
Brevundimonas phage vB_BpoS-Leszy
Brevundimonas phage vB_BgoS-BabaJaga
Brevundimonas phage vB_BpoS-Poludnitsa
Brevundimonas phage vB_BsubS-Delta
Brevundimonas phage vB_BgoS-Fanboy
Pseudomonas phage ZQG1
Pseudomonas phage PaMx74
Janthinobacterium phage vB_JliM-Donnerlittchen
Pseudomonas phage PaMx28
Pseudomonas phage PA_LZ02
Pseudomonas phage phiH2
Pseudomonas phage phiH1
Pseudomonas phage AAT-1
Xanthomonas phage vB_Xar_IVIA-DoCa5
Xanthomonas phage Mallos
Stenotrophomonas phage A1432
Xanthomonas phage Elanor
Xanthomonas phage Xoo-sp2
Stenotrophomonas phage vB_Sm_QDWS359
Xanthomonas phage Xp12
Xanthomonas phage vB_XeuM-YB23
Xanthomonas phage MET13-T1
Xanthomonas phage FMYAK-P1
Xanthomonas phage vB_Xar_IVIA-DoCa6
Stenotrophomonas phage vB_SmaS-AXL_1
Xanthomonas phage vB_Xar_IVIA-DoCa10
Xanthomonas phage Bosa
Stenotrophomonas phage DLP4
Tree scale: 0.1

(c)

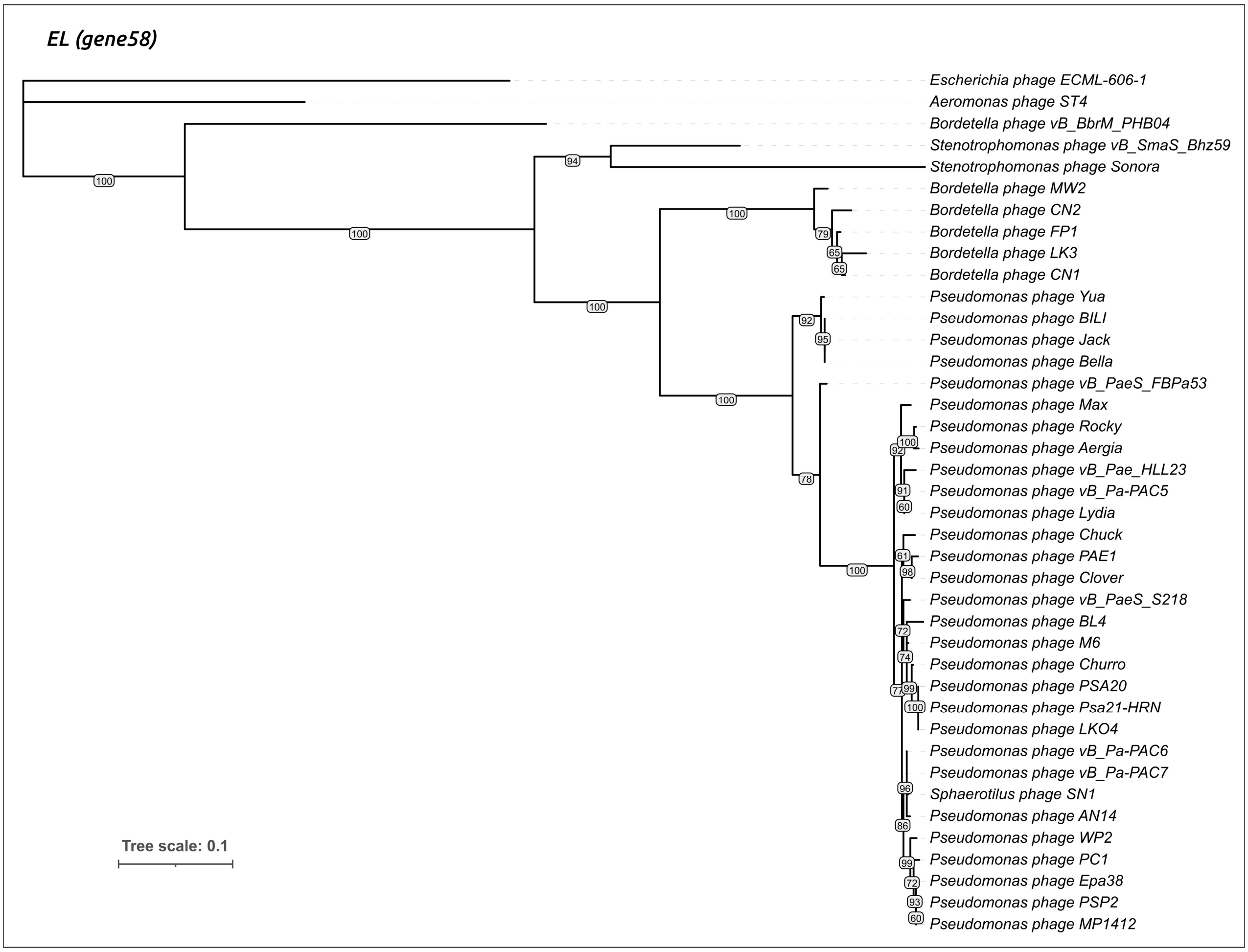

(d)

Supplementary Figure S6. Maximum likelihood phylogenetic trees based on nucleotide sequences of predicted phage genes. The scale bar shows the number of estimated substitutions per site. Taxonomy is indicated in labels and legends. Branches with a bootstrap support lower than 50% have been deleted. Bootstrap values are shown near their branches. The trees were unrooted. (a) DUHMT (gene4). (c) TLS (gene47). (e) MCP (gene52). (f) EL (gene58).
